# European Psychiatric Association guidance on assessment of cognitive impairment in schizophrenia

**DOI:** 10.1192/j.eurpsy.2022.2316

**Published:** 2022-09-05

**Authors:** Antonio Vita, Wolfgang Gaebel, Armida Mucci, Gabriele Sachs, Andreas Erfurth, Stefano Barlati, Federico Zanca, Giulia Maria Giordano, Louise Birkedal Glenthøj, Merete Nordentoft, Silvana Galderisi

**Affiliations:** 1 Department of Clinical and Experimental Sciences, University of Brescia, Brescia, Italy; 2 Department of Mental Health and Addiction Services, Spedali Civili Hospital, Brescia, Italy; 3 WHO Collaborating Centre on Quality Assurance and Empowerment in Mental Health DEU-131, LVR-Klinikum Düsseldorf, Düsseldorf, Germany; 4 Department of Psychiatry and Psychotherapy, Heinrich-Heine-University, Düsseldorf, Germany; 5 University of Campania “Luigi Vanvitelli”, Naples, Italy; 6 Medical University of Vienna, Wien, Austria; 7 CORE – Copenhagen Research Centre for Mental Health, Copenhagen University Hospital, Copenhagen, Denmark; 8 Department of Psychology, Copenhagen University, Copenhagen, Denmark; 9 Department of Clinical Medicine, University of Copenhagen, Copenhagen, Denmark

**Keywords:** Assessment instruments, cognitive functioning, evidence-based, psychosocial functioning, systematic review

## Abstract

**Background:**

Impairment in a wide range of cognitive abilities has been consistently reported in individuals with schizophrenia. Both neurocognitive and social cognitive deficits are thought to underlie severe functional disabilities associated with schizophrenia. Despite the key role in schizophrenia outcome, cognition is still poorly assessed in both research and clinical settings.

**Methods:**

In this guidance paper, we provide a systematic review of the scientific literature and elaborate several recommendations for the assessment of cognitive functions in schizophrenia both in research settings and in real-world clinical practice.

**Results:**

Expert consensus and systematic reviews provided guidance for the optimal assessment of cognitive functions in schizophrenia. Based on the reviewed evidence, we recommend a comprehensive and systematic assessment of neurocognitive and social cognitive domains in schizophrenia, in all phases of the disorder, as well as in subjects at risk to develop psychosis. This European Psychiatric Association guidance recommends not only the use of observer reports but also self-reports and interview-based cognitive assessment tools. The guidance also provides a systematic review of the state of the art of assessment in the first episode of psychosis patients and in individuals at risk for psychosis.

**Conclusion:**

The comprehensive review of the evidence and the recommendations might contribute to advance the field, allowing a better cognitive assessment, and avoiding overlaps with other psychopathological dimensions. The dissemination of this guidance paper may promote the development of shared guidelines concerning the assessment of cognitive functions in schizophrenia, with the purpose to improve the quality of care and to obtain recovery.

## Introduction

### Background

Cognitive impairment has been considered a core feature of schizophrenia since the first descriptions of the disorder [1,2]. Several meta-analyses and systematic reviews consistently demonstrated that subjects with schizophrenia, compared to healthy controls, present a mild to severe impairment in different domains of cognition [3–9]. The involved domains encompass a wide range of functions, including neurocognitive domains, such as attention, speed of processing, memory, working memory, reasoning and problem solving, as well as social cognition domains, such as emotion processing, and theory of mind (ToM). Cognitive impairment is present since the first manifestations of the disease and in subjects at clinical high risk (CHR) for psychosis [3,6,10,11], as well as, in an attenuated form, in non-affected relatives of subjects with schizophrenia [12,13]. The overall magnitude and pattern of cognitive impairment remain substantially stable over the course of schizophrenia, after the first episode of the illness, with the exception of working memory and social cognition, which are less impaired in the early stages of the illness than in the chronic phases [14].

The deficits in multiple neurocognitive domains seem to interfere with real-life functioning more than negative and positive symptoms [15–18]. The impact of neurocognitive deficits on real-life functioning is mediated at least in part by social cognition; however, neurocognitive and social cognitive impairments are associated with different functional outcomes [15,17–21]. While currently available pharmacological treatments appear to exert only limited improvements in cognitive performance, psychosocial interventions such as cognitive remediation appear to provide consistent benefits, especially when integrated with a structured psychiatric rehabilitation program, as attested by several meta-analytic studies [22–26]. Physical exercise also appears to have positive effects [27]. However, despite their important role in determining worse real-world outcomes and the existence of targeted and effective evidence-based treatment, cognitive deficits often remain an overlooked aspect in day-to-day clinical practice in mental health services.

In this perspective, the Schizophrenia Section of the European Psychiatric Association (EPA) proposed the development of a guidance paper aimed to provide recommendations for the assessment of cognitive impairment in people living with schizophrenia.

### Aims

The aim of this work is to present a comprehensive and detailed overview of cognitive impairment in people living with schizophrenia and provide evidence-based recommendations for its assessment both in research settings and in everyday clinical practice.

The guidance will be structured into four sections:

- Conceptualization of cognitive impairment: detailing the identification of distinct domains and the factor structure of cognitive deficits.
- Impact of cognitive impairment in schizophrenia: describing the negative role of cognitive deficits on psychosocial functioning, real-world outcomes, and quality of life (QoL).
- Recognition and assessment of cognitive impairment: providing a review of available validated assessment instruments as well as recommendations regarding the feasibility and applicability of cognitive assessment tools in real-world psychiatric settings.
- Assessment of cognitive impairment in early intervention settings: focusing on recognition and assessment of cognitive impairment in high-risk and early psychosis subjects.

## Methodology

### Systematic literature search

The development of EPA guidance on the assessment of cognitive impairment in schizophrenia followed the standardized methods defined by the European Guidance Project of the EPA, as described in previous publications [28–33], and is based on a systematic literature search performed according to the Preferred Reporting Items for Systematic reviews and Meta-Analyses (PRISMA) indications [34,35].

The literature search was conducted on three electronic databases, Medline/PubMed, Scopus, and PsychINFO, using the following research string: (Schizophrenia and (“cognitive impairment”, “cognitive function”, “cognitive symptoms”, “memory”, “attention”, “executive functions”, “processing speed”, “learning”, “reasoning”, “problem solving”, “social cognition”, “emotion processing”, “theory of mind”, “attributional style”, “social perception”, “metacognition”, “metacognitive”, “cognitive assessment”, or “neuropsychological assessment”)), considering results from January 01, 2010 to December 31, 2020 to avoid excessively outdated findings.

Studies were selected for inclusion in the EPA guidance according to pre-defined criteria.

### Selection procedure

To be considered for inclusion, studies had to be meta-analyses, randomized controlled trials (RCTs), reviews, cohort studies, open studies, descriptive studies, or expert opinions regarding the assessment of cognitive deficits in people living with schizophrenia, with CHR or early psychosis. Reports were considered for inclusion if they were published in English language.

Reports were excluded if they were duplicates, comments, editorials, case reports, case series, theses, proceedings, letters, short surveys, and notes, or if they were studies irrelevant to the topic.

Retracted and outdated studies, studies whose results were included or pooled in subsequent works, and studies with relevant methodological issues were also excluded.

All documents were independently inspected by at least two screeners and discrepancies in the selection process were discussed and resolved with the support of a third researcher.

**Figure 1.**
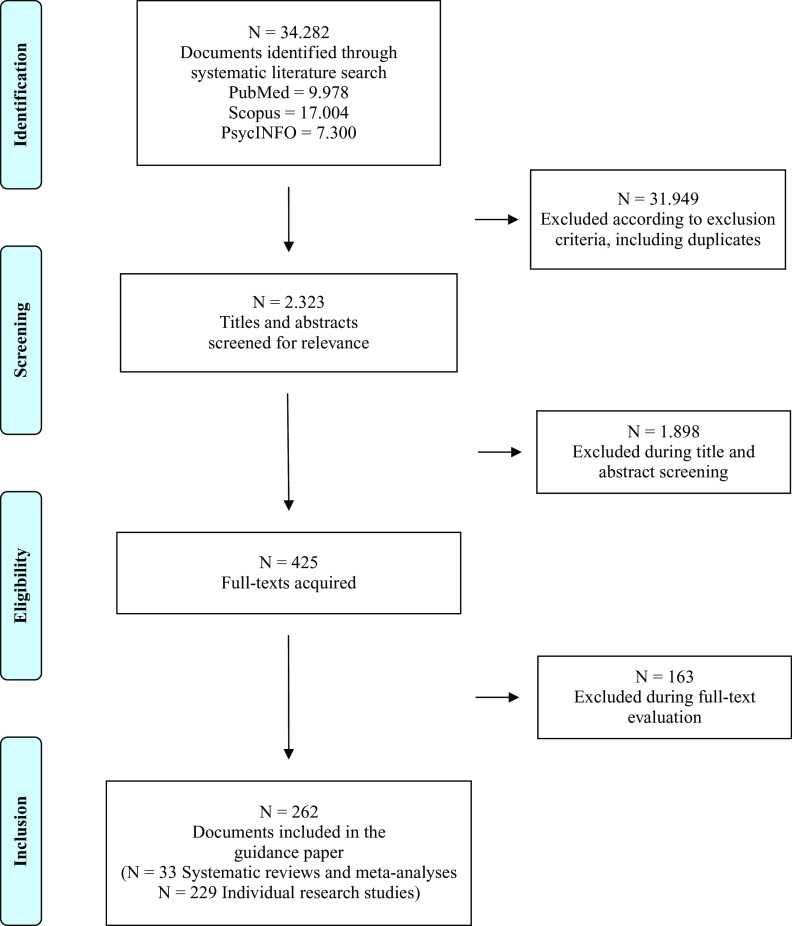
PRISMA flow diagram.

Results of the selection procedure are shown in Figure 1.

### Grading of evidence

Included studies were graded regarding the level of evidence provided, according to previous literature [29]. Grades were assigned according to the indications detailed by Gaebel et al. [28] and modified by Galderisi et al. [32]. The grading criteria of included evidence are reported in Table 1. Discrepancies in the ratings were resolved by discussion among all coauthors.

**Table 1.** Grading of evidence.

Grade	Features of quantitative studies	Features of reviews
I-Generalizable studies	Randomized controlled trials. Surveys sampling a large and representative group of persons from the general population or from a large range of service settings. Analytic procedures comprehensive and clear usually including multivariate analyses or statistical modeling. Results can be generalized to settings or stakeholder groups other than those reported in the study	Systematic reviews or meta-analyses
II-Conceptual studies	Uncontrolled, blinded clinical trials. Surveys sampling a restricted group of persons or a limited number of service providers or settings. May be limited to one group about which little is known or a number of important subgroups. Analytic procedures comprehensive and clear. Results have limited generalizability	Unsystematic reviews with a low degree of selection bias employing clearly defined search strategies
III-Descriptive studies	Open, uncontrolled clinical trials. Description of treatment as usual. Survey sampling not representative since it was selected from a single specialized setting or a small group of persons. Mainly records experience and use only a limited range of analytical procedures, like descriptive statistics. Results have limited generalizability	Unsystematic reviews with a high degree of selection bias due to undefined or poorly defined search strategies
IV-Single case study	Case studies. Provides survey data on the views or experiences of a few individuals in a single setting. Can provide insight into unexplored contexts. Results cannot be generalized	Editorials

### Grading of recommendations

Based on the evidence level of the included documents, recommendations were developed and reviewed by all coauthors. Grades were then assigned to recommendations according to the indications detailed by Gaebel et al. [28] and modified by Galderisi et al. [32]. The grading criteria of recommendations are reported in Table 2.

**Table 2.** Grading of recommendations.

Grade	Description
A	At least one study or review rated as I and directly applicable to the target population OR a body of evidence consisting principally of studies and/or reviews rated as I, directly applicable to the target population, and demonstrating overall consistency of results
B	A body of evidence including studies and/or reviews rated as II, directly applicable to the target population, and demonstrating overall consistency of results OR extrapolated evidence from studies and/or reviews rated as I or II
C	A body of evidence including studies and/or reviews rated as II–III, directly applicable to the target population, and demonstrating overall consistency of results OR extrapolated evidence from studies and/or reviews rated as II or III
D	Level of evidence rated as III or IV OR extrapolated evidence from studies and/or reviews rated as III or IV OR expert consensus

### Conceptualization of Cognitive Impairment

The definitions of the neurocognitive and social cognition domains, selected by expert consensus [36,37] and widely recognized as impaired in schizophrenia [15], are provided in Box 1.

Box 1. Cognitive domains identified by the Measurement and Treatment Research to Improve Cognition in Schizophrenia (MATRICS) initiative and Social Cognition Psychometric Evaluation (SCOPE) consensus initiatives.

**Table:** 

Neurocognitive domains (MATRICS)	Impairment observed in subjects with schizophrenia
*Speed of processing*	*Slowdown of the speed with which a person can perform perceptual-cognitive or motor tasks. Impairment in processing speed may reflect a preference for step-by-step processing rather than planning before task execution. This style of responding could be adopted to compensate working memory impairment*
*Attention/vigilance*	*Difficulty to maintain the attentional focus and to inhibit irrelevant information*
*Working memory*	*Deficit in the ability to temporarily store and manipulate information in the execution of complex cognitive tasks such as learning, reasoning, and comprehension*
*Verbal learning and memory*	*Impairment of memorization and recall of verbally presented information*
*Visuospatial learning and memory*	*Impairment of memorization and recall of visually presented information*
*Reasoning and problem solving*	*Poor ability to generate and apply alternative strategies to cope with new or unexpected problems*

Social cognition domains (SCOPE)	Impairment observed in subjects with schizophrenia
*Emotional processing*	*Impairment in perceiving and using emotional information*
*Theory of mind (ToM)*	*Impairment in mentalizing, poor mental state attribution, or deficit in cognitive empathy are terms used interchangeably. They refer to the difficulty to infer the intentions, dispositions, emotions, and beliefs of others*
*Social perception*	*Poor ability to decode and interpret social cues*
*Attribution style/bias*	*Increased tendency to attribute hostile intentions to others, inferring the causes of particular positive and negative social situations*

## Cognitive Impairment in schizophrenia: identification of the distinct domains of impairment

The NIMH-Measurement and Treatment Research to Improve Cognition in Schizophrenia (MATRICS) initiative adopted a structured consensus-building process to identify the domains of cognitive impairment in schizophrenia and developed a consensus cognitive battery for use in clinical trials in schizophrenia. To this aim, factor analytic studies of cognitive performance in schizophrenia published until 2004 were examined and factors that were replicated across several studies were selected. Thirteen-factor analyses were scrutinized, and six separable factors were selected through consensus by the expert panel (see Box 1). Based on the feedback received, the Neurocognition Committee added social cognition as one of the domains, yielding a total of seven domains [36]. Relevant papers published after 2004, including several meta-analyses, consistently demonstrate that the six neurocognitive domains identified by the MATRICS initiative as well as the four social cognition domains are impaired in schizophrenia (see Supplementary Table 1). Apart from these domains, one meta-analysis indicated an impairment of autobiographical memory and another one of semantic memory [38,39]. Several meta-analyses focused on aspects of executive functions different from those selected by the MATRICS initiative [40–44]. The relevant literature identified a wide range of subdomains: categorization, strategy forming, complex forward planning, cognitive flexibility, problem solving, working memory, attention, and set shifting, which overlap in part with each other and with the domains of “working memory” and “reasoning and problem solving” identified by the MATRICS consensus initiative.

However, factor analytic studies published in the period following the MATRICS consensus initiative did not report robust and consistent results (Supplementary Table 2). This heterogeneity seems to derive from differences in methodological and patient-related factors. Further studies, using large multicenter samples and a large array of standardized tests for the assessment of multiple domains of cognition, are needed to evaluate the factor structure with respect to what has been established by the MATRICS initiative.

Regarding social cognition, despite the agreement on its definition and the consistent demonstration of impairment in subjects with schizophrenia, there has been a lack of consensus on the identification of the independent domains which should be assessed [37]. Therefore, the NIMH “Social Cognition Psychometric Evaluation” (SCOPE) study was initiated in 2013 in order to identify independent social cognition domains and identify the best existing measures of those domains [37]. A panel of experts reviewed all available scientific information, following the “Research and Development (RAND) Corporation/University of California Los Angeles (UCLA) Appropriateness Method” (RAM), and four social cognition domains were identified (see Box 1).

Several meta-analyses [6,45–52] reported that people with schizophrenia present deficits in social cognition, especially in emotion processing, ToM, social perception and, to a lesser extent, in social knowledge and attributional bias.

Literature focusing on ToM demonstrated severe and stable impairment in first-episode outpatients, and a mild impairment in both CHR individuals and unaffected relatives [45,46]. ToM has also been found to be associated with overall neurocognition and each of its subdomains, and to be a moderator of neurocognitive task performance in subjects with schizophrenia [52]. ToM might also play a role in poor insight [53] and could have a stronger correlation with functional outcome than emotion recognition [54].

## Factor structures of cognitive impairment in schizophrenia

Since there is consistent evidence for the existence of distinct social cognition and neurocognition constructs, social cognition has been analyzed as a separate domain [55].

Several factor-analytic studies examined the factor structure of neurocognitive deficits in subjects with schizophrenia. However, findings across studies were discrepant and, until now, there is no broad consensus on the number of independent domains and on the model of their factor structure (single, multiple, or hierarchical) (Supplementary Table 2).

The confirmatory factor analysis (CFA) conducted by Keefe et al. supported a single, second-order factor model, according to which the cognitive impairment could be sufficiently represented by a single cognitive factor, which hierarchically comprised five domains: speed of processing, vigilance, working memory, verbal memory, and reasoning [56]. In the multifactorial models, a certain number of cognitive domains were found to cluster as separable but inter-correlated factors. Some authors found that the seven-factor model had the best goodness-of-fit [57,58], while others found that the six [59] or three [60–62] had the best fit. Three of these studies [60–62] used CFA on the MATRICS Consensus Cognitive Battery (MCCB) domains, demonstrating that a three-factor model, including processing speed, attention/working memory, and learning, provided a better fit than the unifactorial structure. Two CFA studies described a hierarchical model, consistent with models of intelligence in healthy samples, in which individual cognitive tests loaded on six cognitive domain factors, which in turn loaded on the general cognitive ability factor [63,64].

The presence of discrepant findings between studies may be primarily due to heterogeneity in assessment instruments, or in sample sizes and characteristics. As the six domains of impairment were identified through expert consensus, future studies should provide external validation of the same domains and should investigate whether these domains demonstrate distinct associations with functional outcome measures or specific sensitivity to different pharmacological and psychosocial treatments.

Very few heterogeneous studies regarding the factor structure of social cognition have been conducted subsequently to the SCOPE initiative [55]. Some exploratory factor analytic studies found multifactorial social cognition models [65–68]. Each study identified different factors, which seem to represent diverse latent constructs: the observed heterogeneity does not allow conclusions. The CFA study conducted by Browne et al. in subjects with schizophrenia and healthy controls, based on SCOPE structure, supports a one-factor model for both groups [69]. Differences in the assessment of social cognition across studies and the inclusion of patients at different stages of the illness could explain discrepancies in results. Therefore, future studies need to focus on the use of psychometrically sound instruments to measure social cognition in large homogeneous populations.

## Recommendations

Considering the available literature, the working group elaborated the following recommendations:

**Table:** 

Grade	Recommendation 1 (based on studies included in Supplementary Table 1)
A	The six neurocognitive domains identified by the MATRICS initiative should be carefully assessed in subjects with schizophrenia, in all phases of the disorder, as well as in CHR subjects

**Table:** 

Grade	Recommendation 2 (based on studies included in Supplementary Table 2)
A	Social cognition should be carefully assessed in subjects with schizophrenia, considering at least the following domains: ToM, social perception, and emotion processing

No recommendation is deemed appropriate by the EPA Guidance Group on Cognitive Impairment on the factor model of neurocognition and social cognition to be used in clinical trials. Further studies are deemed essential to validate the six neurocognitive domains identified by the MATRICS initiative and the four domains identified by the SCOPE initiative. Validation should be based on patterns of differential associations with functional outcome measures or pathophysiological mechanisms or differential sensitivity to pharmacological and non-pharmacological treatments.

## Impact of Cognitive Impairment in Schizophrenia

This chapter identifies scientific results on neurocognitive and social cognitive impairments and their impact on real-life functioning and QoL.

To facilitate the analysis, results are shown in the following way:

- Effects of neurocognition and social cognition on functional outcome
- Effects of neurocognition on QoL
- Effects of social cognition on QoL

### Effects of neurocognition and social cognition on functional outcome

Cognitive impairment is a central feature of schizophrenia [15] and has been identified as the strongest predictor of functional outcome [70,71]. A large body of literature shows that cognitive dysfunction in patients with schizophrenia accounts for 20–60% of the variance in measures of functional outcome [17,72]. These impairments are present across the course of the illness, from prodromal to early onset psychosis and to more chronic patients. Cognitive deficits are evident from an early stage [11] and are often strongly associated with functional impairment [16,17,19]. This association is very robust as it was replicated in numerous studies, using different types of assessments and different patient groups across all phases of the illness, including high-risk subjects and subjects in prodromal states [73], first-episode patients (FEPs) [74], and older patients with chronic schizophrenia [75,76]. Cognitive deficits constitute one of the main limiting factors for recovery in the context of psychiatric treatment and rehabilitation. Despite treatment of symptoms with antipsychotics, impairments in daily functioning still represent a major treatment issue. Many studies demonstrated that functional outcome is more closely related to cognition than to positive or negative symptoms [71,77,78]. The question has moved from whether cognitive dysfunction is related to functional outcome to how cognition is related to functional outcome. Composite measures of cognitive performance seem to account for 25–50% of the variance in real-world functioning, and the relationship between neuropsychological functioning and functional outcome seems to be mediated by functional capacity [70,79,80]. Other factors have a significant impact on real-life functioning, such as empathy, negative symptoms, and depression [73,81–83]. In a sample of outpatients with chronic schizophrenia [83], factors influencing both functional capacity and real-life behavior were investigated. Real-life behavior was significantly predicted by interpersonal reactivity. Functional capacity seems mainly related to neurocognition. Studies that have linked neurocognition to social cognition and/or social cognition to functional status have shown social cognition in schizophrenia to be a mediator of relations between neurocognition and functional status [17, 21,84,85].

In the meta-analysis by Fett et al. [19], social cognition explained relatively more variance in community outcome than neurocognition. This difference was largely due to the ability to infer other’s mental states, for instance mentalizing or ToM.

The estimated average correlations between community functioning and each of the neurocognitive and social cognitive domains were strongest for mentalizing, verbal fluency, social perception, and knowledge. A network analysis that investigated 740 patients with schizophrenia [72] found that functional capacity and everyday life skills were the most central and highly interconnected nodes in the network. Functional capacity bridged cognition with everyday life skills, and the everyday life skills node was linked to disorganization and expressive deficits. Interpersonal relationships and work skills were connected to avolition; the interpersonal relationships node was also linked to social competence. The high centrality of functional capacity and everyday life skills in the network suggests that improving the ability to perform tasks is relevant for any therapeutic intervention in schizophrenia.

The meta-analysis by Halverson et al. [20] explored relationships between functional outcome in schizophrenia spectrum disorders and different domains of neurocognition and social cognition. Overall, associations between social cognition, neurocognition, and functional outcome showed significant small-to-medium effect sizes. Social cognition explained more variance in functioning than neurocognition [86,87]. In the mediation analysis associations between neurocognition, social cognition and different domains of functional outcome were found. Verbal learning and memory were shown to correlate with community functioning, working memory with social behavior in the milieu. For social cognition, the strongest associations were present for social knowledge and perception and community functioning. ToM was shown to correlate with social behavior in the milieu. Reasoning and problem solving demonstrated the strongest relationships with social problem solving, working memory with social skills, and ToM was shown to correlate with social skills.

Similar associations between neurocognition and social cognition with functional outcome are already present in the early stages of illness. The relationship between cognition and outcome was observed also in FEPs highlighting the importance of early intervention [88,89]. The influence of cognitive reserve as a mediator between cognitive domains and function in FEP was shown by Amoretti et al. [90] and by Gonzalez-Ortega et al. [91].

In a study by Modinos et al. [92], individuals at CHR of psychosis were assessed. It was found that abnormalities in social cognition at baseline were associated with poor functional outcome after 12 months. Poor functional outcome was associated with baseline abnormalities in the recognition of angry emotion. These findings have potential implications for the stratification of individuals at CHR of psychosis according to subsequent outcome and suggest that functional outcome might be improved by interventions that target emotional processing.

Distinctions between self-report and observer reported measures of real-world functional outcome have become an area of focus in schizophrenia research. Self-report has been shown to be minimally correlated with observer reported real-world functional outcome.

Ho et al. [93] demonstrated that functional capacity partially mediates the relationship between overall cognitive ability and observer reported real-world functioning in work skills and community participation. It appears that cognitive impairment might be a precursor to poor acquisition of functional capacity, for instance what a person can do, which subsequently affects real-world functioning, for instance what a person actually does. Self-report of ability seems to be a further determinant of real-world functional outcome. This newly defined component addresses just how well individuals evaluate their own abilities and performance, and this type of self-awareness was referred as “introspective accuracy” (IA). The results of a study by Silberstein and Harvey [94] indicate that IA of neurocognition strongly predicted nonsocial functional outcomes (everyday activities). IA of social cognition showed a significant correlation of medium strength to interpersonal relationships while showing a small relationship to everyday activities. These findings support the idea to combine observer ratings and patients’ self-reports because the discrepancy score adds some understanding of everyday social deficits.

The role of cognitive ability in observer versus self-reported real-world functioning may be explained by different mechanisms. Deviation between observed and expected cognitive ability is a core cognitive feature of schizophrenia related to neurophysiological, clinical, and psychosocial functioning [95]. Hochberger et al. [95] found that 24% of the total patient population exhibited significant deviation between observed and expected cognitive ability. The magnitude of this deviation was associated with worse psychosocial functioning. Since the relationship between psychopathology, neurocognitive deficits, and functional outcome is very complex, new statistical methods have been applied including computational model tools such as artificial neural networks [96]. In the study by Bosia et al. [96], processing speed turned out to be the first rank-predictor of functional outcome. Attention and verbal memory were also shown to have an impact on functioning. This finding stands in line with previous and recent findings.

By using a structural equation approach, Ojeda et al. [97] found that processing speed, verbal memory, and premorbid functioning predicted outcome. Social cognition and processing speed explained 47% of the variance in community functioning in a study by Lewandowski et al. [98].

Vita et al. [99] showed that autistic symptoms may identify a subgroup of people with schizophrenia with worse outcome. Subjects with schizophrenia with more severe autistic symptoms showed poorer processing speed, attention, verbal memory, social cognition, poorer functional capacity, real-world interpersonal relationships, and participation in community-living activities [99].

Supplementary Table 3 shows all systematic reviews and meta-analyses (level of evidence I) available on the effects of neurocognition and social cognition on functional outcome.

### Recommendations

Considering the available literature, the working group elaborated the following recommendations:

**Table:** 

Grade	Recommendation 3 (based on studies included in Supplementary Table 3)
A	Neurocognitive and social cognitive impairments should be assessed in research settings and clinical practice as they have an important impact on multiple functional outcomes, such as community functioning, work skills, interpersonal relationships, and functional capacity

**Table:** 

Grade	Recommendation 4 (based on studies included in Supplementary Table 3)
A	Both self-reports and observer reports of cognitive ability should be taken into account as they give additive and complementary information: self-report of neurocognition predicts everyday activities and self-report of social cognition predicts interpersonal relationships

### Effects of neurocognition on QoL

Studies on the effects of neurocognition on QoL are shown in Supplementary Table 4. Some small studies with evidence level III showed an association of neurocognition with QoL, and a large meta-analytic study revealed a moderate correlation between verbal ability and processing speed with subjective QoL, while a large study with 1032 subjects with schizophrenia showed no association of neurocognitive functioning with QoL [100] or a negative correlation [101]. In a population of older adults with schizophrenia, neurocognitive impairment was associated with reduced overall functioning and low education with diminished QoL [102].

### Recommendations

Considering the available literature, the working group elaborated the following recommendations:

**Table:** 

Grade	Recommendation 5 (based on studies included in Supplementary Table 4)
B	Neurocognitive functioning should be assessed in research settings and clinical practice as it can have a correlation with QoL in people living with schizophrenia. However, further studies are suggested to examine the possible relationship between neurocognition and QoL

### Effects of social cognition on QoL

Results of the few studies on the effects of social cognition on QoL (QOL) are shown in Supplementary Table 5. Only one study has investigated the relationship at evidence level I, showing an association of ToM but not emotion perception or neurocognition with QoL in subjects with schizophrenia.

### Recommendations

Considering the available literature, the working group elaborated the following recommendations:

**Table:** 

Grade	Recommendation 6 (based on studies included in Supplementary Table 5)
C	Social cognition should be assessed as it could also have an impact and subjective and objective QoL. However, further studies are suggested to examine the possible relationship between social cognition and subjective and objective QoL

## Recognition and Assessment of Cognitive Impairment

### Assessment instruments

The systematic assessment of cognitive impairment still represents an unmet need for people with schizophrenia. Until 2004, this type of assessment was confined to specialized centers and limited to a few domains [71,103,104]. Many different instruments were employed in the past decades: most of them were adapted from clinical neuropsychology and were too long and complex, as they assessed the entire neuropsychological profile of an individual. A systematic review, conducted by Bakkour in 2014 [105], found that the batteries most often used in trials assessing cognition in schizophrenia were the C*ambridge Neuropsychological Test Automated Battery (CANTAB), the CogState, and the Repeatable Battery for the Assessment of Neuropsychological Status (RBANS)*; sometimes single subtests were adopted [105]. However, these instruments were not specifically designed for schizophrenia and were developed to assess cognitive deterioration in elderly subjects with dementia, with possible ceiling effects in subjects with schizophrenia. Furthermore, the batteries were too complex and time-consuming for their use in clinical trials and routine clinical assessments.

A renewal of interest in the cognitive assessment of people with schizophrenia was related to the increasing acknowledgment of the strong relationships of cognitive deficits with functional outcome [71,103,104]. In the early 2000s, research focused on those aspects of cognition that demonstrated a strong correlation with a variety of functional outcome measures (community functioning, functional capacity, social skills acquisition). Later on, social cognition, which was not included in neuropsychological batteries, became also a focus as it represents a mediator of the impact of neurocognition on functioning [16,21,71,72,106]. The renewed interest and the association with functional outcome stimulated the development of batteries specifically devoted to the cognitive assessment of subjects with schizophrenia [107–109].

## Instruments Developed for Assessing Neurocognitive Impairment in Subjects with Schizophrenia

### Performance-based instruments

Until 2004, no standardized battery for assessing neurocognitive impairment in schizophrenia was developed, contrary to what happened for other diseases, such as dementia. The first instrument specifically designed for the disorder was the Brief Assessment of Cognition in Schizophrenia (BACS), developed by Keefe and his group [108]. The BACS had a rather short administration time (35 min) and a high completion rate. Its results correlated with those obtained using a standard battery [108]. The included subtests explore six domains of neurocognition (Supplementary Table 6) and were chosen on the basis of test–retest reliability, practice effects, and sensitivity to impairment in schizophrenia (Supplementary Table 7). The BACS also showed a correlation with measures of functional outcome, the University of California Performance-based Skills Assessment (UPSA, a measure of functional capacity), and the Independent Living Skills Inventory (ILSI, a measure of real-life functioning) [110] and was found to have better psychometric properties, compared to RBANS, in subjects with schizophrenia [111]. It has been translated and validated into nine languages [108,112–119] (Supplementary Table 6). Normative data in different cultural contexts (USA, Italy, Taiwan-Mandarin-speaking Chinese population; Singapore-English-speaking Chinese population) are available [119–122]; they show a good degree of consistency in western countries [119,121], while those collected Taiwan and Singapore appear significantly different from US data, demonstrating the need for adaptation. Western samples performed better on language-related tasks, while Chinese samples performed better on non-language-related tasks, indicating that theapplication of BACS western norms to determine the performance of Chinese populations might result in data misinterpretation [120,122]. Even if BACS proved to be a valid tool, it was developed by a single group, without a large consensus on the domains to be assessed and on criteria for test selection [36].

To analyze the efficacy of new compounds on cognition, the Food and Drug Administration required the development of an instrument through the acquisition of a consensus among experts. To this aim, the MATRICS initiative developed a consensus cognitive battery for subjects with schizophrenia, designed for use in clinical trials [109]. The process of test selection was conducted following the RAND/UCLA appropriateness method (an internationally recognized method to define the appropriateness of tests or clinical procedures using consensus among expert panels): 74 experts were questioned about the criteria for including subtests in the battery [123]. Five criteria were selected as most important: 1) test–retest reliability; 2) high utility as a repeated measure; 3) relationship with the functional outcome; 4) potential response to pharmacologic agents; and 5) tolerability and practicality [36]. Of note, these criteria only partially overlap with those applied for BACS. Through the evaluation of adherence to the five criteria cited above, 10 tests were selected. The resulting MCCB explored the seven domains of cognition that were identified by the consensus conference. Supplementary Tables 6 and 7 provide details on the cognitive domains assessed by the MCCB and the psychometric properties of this instrument. At the present time, the MCCB is the only performance-based assessment battery that includes a subtest exploring a social cognition domain. Furthermore, despite a rather long administration time (60–90 min), 95% of participants returned for re-administration: this was underlined as high tolerability index (Supplementary Table 7). Several studies demonstrated that the MCCB is a sensitive instrument, with a good correlation to functional outcome [107,124]; in addition to that, its psychometric properties were found adherent to key criteria individuated by the MATRICS initiative [125,126]. Based on its psychometric properties and the strong relationship with functional outcome, the MCCB has been proposed as the gold standard in assessing cognitive impairment in subjects with schizophrenia. It has been translated into 24 languages (Supplementary Table 6) and validated in different countries, USA, Brazil, Italy, Czech Republic, Poland, Japan, Norway, China, Singapore, and Spain [13,109,127–134]. Normative data in different cultural contexts (USA; Brazil; Italy, Czech Republic; Norway; China; Singapore; Spain) are available [13,127,128,131–135]. Consistent normative data for MCCB are available for western countries, while normative data from Singapore significantly differ from US data [132]. The overall performance of Singaporeans, compared to the US population, was poorer in all subtests except the Spatial Span subtest in which no mean difference between samples was detected [132]. The most important obstacle to the use of MCCB in routine clinical assessment of patients with schizophrenia is the long administration time (60–90 min). Therefore, other short-administered tools were developed, aimed at use in everyday clinical routine (the Screen for Cognitive Impairment in Psychiatry [SCIP], the Brief Neurocognitive Assessment (BNA), and the Brief Cognitive Assessment Tool for Schizophrenia [B-CATS]).

The SCIP is a tool for the screening of cognitive impairment in subjects with affective disorders and psychoses. In particular, this test was specifically developed in order to provide a short instrument assessing neurocognitive impairment, thus facilitating the implementation into everyday clinical practice. It is a brief, pencil‐and‐paper test that requires about 15 min for its administration [136]. Three equivalent forms are available and include the assessment of processing speed, attention, verbal fluency, and verbal memory domains (Supplementary Table 6). SCIP showed adequate psychometric properties in terms of test–retest reliability, temporal stability and internal consistency, specificity and sensitivity, as well as criterion and discriminant validity [137–141] (Supplementary Table 7).

The BNA has a 10-min administration time and was developed with the aim of capturing the maximum possible variance, using only two subtests that explored two domains (Supplementary Table 6). The BNA was able to capture 76% of the total variance found using a standard neurocognitive battery (The Clinical Antipsychotic Trials of Intervention Effectiveness-CATIE-cognitive battery) (Supplementary Table 7) [142].

Another instrument, the B-CATS, was similarly developed, choosing subtests from batteries employed in previous trials and selecting those tests that account for a large proportion of variance in the global score per minute of administration time. It has an administration time of about 10 min and offers a global score of cognition, rather than exploring single domains [143] (Supplementary Table 6–7). Both BNA and B-CATS were subsequently compared to the MCCB and the measures were found to be highly correlated. The BNA showed a test–retest reliability that was similar to MCCB, low practice effects, and sensitivity to longitudinal changes [144]. The B-CATS was found to have good internal consistency and test–retest reliability; it was highly correlated with the composite score of MCCB and total score of UPSA [145]. However, the subtests included in both the above short batteries are included in the MCCB: this is to be considered when correlations between these instruments are evaluated. Therefore, further validation of these tools is advised. A study that compared the ability of two of these short tests (SCIP and B‐CATS) found that the SCIP was better than the B‐CATS in predicting global cognitive impairment in subjects with psychosis [146]. According to a systematic review conducted in 2014 [105], the MCCB was the instrument adopted in most trials (*N* = 69), followed by the BACS (*N* = 24). In addition, the BACS has proven to be a feasible tool in a clinical rehabilitation setting [147]; however, more research is advisable. BNA and B-CATS were not used in any clinical trial. At the present time, given the recent introduction of these short batteries (BNA and B-CATS) and the small amount of evidence, more studies should be conducted in order to assess the validity of these instruments in routine clinical assessment of cognition in schizophrenia.

### Interview-based instruments

Alongside performance-based tests, the MATRICS initiative adopted interview-based cognitive assessment as a co-primary measure that has face validity for patients and clinicians [148]. There is no universal agreement on how much change in performance-based instruments is clinically meaningful, while interview-based measures are designed to evaluate the impact on the functioning of the cognitive deficits and might capture better the clinical meaning of changes over time or following pharmacological or psychosocial treatments [149–151]. Subjects with schizophrenia might have poor insight about their impairment; thus, interview-based measures usually require informants and an expert rater, as it is the case for the assessment of cognitive deterioration in elderly subjects with dementia. Two interview-based instruments, the Clinical Global Impression of Cognition in Schizophrenia (CGI-CogS) and the Schizophrenia Cognition Rating Scale (SCoRS), were evaluated within the MATRICS Psychometric and Standardization Study [148] to verify if they fitted the five MATRICS criteria stated above. These scales were both found to be acceptable and comparable across the various criteria [148].

CGI-CogS was modeled after the development of the Clinical Interview-Based Impression of Severity, a tool widely used for assessing dementia. The interview consists of 38 items, divided into two major categories: (a) activities of daily living and (b) neurocognitive state (Supplementary Table 6). Each item can be evaluated on a scale from 0 to 7 and the administration time is of approximately 30 min. CGI-Cogs shows high internal consistency, good inter-rater reliability, and high test–retest reliability; in addition, the caregiver and rater global scores correlate with neurocognition in the moderate range and with functioning in the moderate-high range [152]. No other study using CGI-CogS was conducted; this is probably due to the development of shorter interview-based instruments. Therefore, further studies are needed.

The other interview-based instrument developed within the MATRICS initiative, the SCoRS, is composed of 18 items, based on the Brief Cognitive Scale, and modified by an experts’ panel. The SCoRS items can be rated on a scale of four levels of severity and assess six cognitive domains (Supplementary Table 6). The administration time is of 20–30 min. The composite score ranges from 1 to 10. The SCoRS provides a measure strongly correlated with real-world functioning [153] and is regarded as a valid co-primary measure as intended by the MATRICS initiative; this is also supported by a narrative review of all research conducted using SCoRS [154]. However, a work by Vita and his group on the validity of the SCoRS demonstrated that the pattern of correlations between SCoRS and functioning varied in different samples; in particular, the relationship with functioning was found only in clinically stable patients, but not in recently hospitalized ones, suggesting a limited value of the SCoRS in acute phases [155]. Further studies are needed to confirm this finding, thus allowing a better characterization of potential factors that influence the correlation between SCoRS and functioning. Furthermore, SCoRS seems to be a valid instrument for assessing the patient and caregiver’s insight into her/his cognitive functioning. A study [156] showed that the patient’s rating scores of SCoRS did not correlate with neurocognitive performance assessed with BACS, while the caregiver’s rating scores correlated only with performance on executive functions. This result suggests a poor awareness of cognitive impairment from both the patient and caregiver perspectives. SCoRS has been translated into 22 languages (Supplementary Table 6) and validated in the US, Italy, Iran, Japan, Korea, and Singapore [114,155,157–159]. Validation studies of SCoRS translated versions found patterns of correlations between cognitive performance and interviewer and informant scores similar to those observed in the US, although some differences were found, likely due to differences in patient populations [114,155,157,158].

To shorten the administration time, the Cognitive Assessment Interview (CAI), a semi-structured interview, was developed by experts within the MATRICS initiative, using both CGI-CogS and SCoRS as “parent instruments.” The development of this interview was based on the item-response theory analysis of the original scales, which showed that only 10–12 items were necessary to achieve an accurate estimate of the neuropsychological deficits [160]. CAI explored six out of the seven domains of cognition identified by the MATRICS initiative (Supplementary Table 6). The severity of the deficit was assessed on a 7-level scale [161]. The measure was subsequently validated by administering it to 150 stable patients diagnosed with schizophrenia and found to be correlated with neurocognition, functional capacity, and everyday functioning. Its short administration time (15 min) and the absence of practice effects made it a reliable instrument in detecting changes across time [162]. CAI has been translated and validated in the US, Italy, Turkey, and Spain [161,163–165] (Supplementary Table 6). A qualitative research study, which investigated the cross-cultural adaptability of four intermediate measures of functioning (Independent Living Scales, UCSD Performance-Based Skills Assessment, Test of Adaptive Behavior in Schizophrenia, and CAI) reported that, while the majority of subscales required major adaptations, CAI required considerably less cultural adaptation [166].

The systematic review [105] on all tools used for assessing cognition in schizophrenia in clinical trials found that SCoRS was used in few trials (*N* = 9) and CGI-CogS was used once. The exclusion of the CAI is probably due to the initial stage of development of the interview-based measures in 2014 [105].

A summary of the characteristics of neurocognition assessment tools is provided in Supplementary Table 6.

### Recommendations

The working group elaborated the following recommendations on the instruments to be used for the assessment of cognitive impairment in schizophrenia, taking into account the level of evidence concerning their psychometric properties, the coverage of the seven cognitive domains identified by the MATRICS Consensus Initiative as the most frequently impaired in subjects with schizophrenia, the administration time, and the availability of translations and validation in the largest number of languages.

**Table:** 

Grade	Recommendation 7 (based on studies included in Supplementary Table 7)
C	Due to its good psychometric properties, the short time of administration, the coverage of five of the seven cognitive domains identified by the MATRICS Consensus Initiative as the most frequently impaired in subjects with schizophrenia, and availability of translations and validation in nine languages, the BACS can be used for the assessment of neurocognitive impairment of subjects with schizophrenia, in the context of clinical trials and to individualize cognitive remediation interventions

**Table:** 

Grade	Recommendation 8 (based on studies included in Supplementary Table 7)
B	Due to its good psychometric properties, coverage of the seven cognitive domains identified by the MATRICS Consensus Initiative as the most frequently impaired in subjects with schizophrenia, and availability of translations and validations in 26 languages, the MCCB should be used for the assessment of neurocognitive impairment of subjects with schizophrenia, in the context of clinical trials and to individualize cognitive remediation interventions

**Table:** 

Grade	Recommendation 9 (based on studies included in Supplementary Table 7)
B	The use of BACS or MCCB should be complemented by instruments for the assessment of social cognition

**Table:** 

Grade	Recommendation 10 (based on studies included in Supplementary Table 7)
B	Due to its good psychometric properties and coverage of four neurocognitive domains, SCIP should be used as a screening instrument for neurocognitive impairment in subjects with schizophrenia in clinical practice and for inclusion of subjects in clinical trials

**Table:** 

Grade	Recommendation 11 (based on studies included in Supplementary Table 7)
B	Due to the coverage of only one (BNA) or two (B-CATS) neurocognitive domains, these instruments should not be used as screening instruments in clinical practice or for the inclusion of subjects in clinical trials

**Table:** 

Grade	Recommendation 12 (based on studies included in Supplementary Table 7)
C	Due to their good psychometric properties and face validity, SCoRS or CAI can be used as co-primary measures in the assessment of cognitive impairment of subjects with schizophrenia in routine clinical context and clinical trials

### Instruments developed for assessing social cognition impairment in subjects with schizophrenia

As emerged in the previous paragraphs, some instruments developed for the assessment of cognition also included a test or an item to evaluate social cognition impairment in subjects with schizophrenia (MCCB, CGI-CogS, and CAI). Therefore, over the years, some research groups have developed different customized instruments to assess exclusively social cognition domains. However, most of them tend to be poorly validated and standardized and show substandard psychometric properties [37,45,48,167–171]. Therefore, as mentioned in the paragraph on conceptualization, in 2013 the NIMH “Social Cognition Psychometric Evaluation” (SCOPE) study was designed to identify not only core domains of social cognition but also instruments that best assess these domains [37]. Based on the literature data, during the first and second phases of the SCOPE study, over 100 measures were nominated, and, after a survey of experts, 21 were forwarded to the RAND panel for consideration. The Mayer-Salovey-Caruso Emotional Intelligence Test (MSCEIT) [172], a test included within the MCCB to explore social cognition, was not considered in this project since the basic psychometrics properties of this instrument have been already established before the SCOPE study [37,109,173]. Eight instruments were positively evaluated and selected for psychometric evaluation via RAND panel ratings, based on previous data concerning the following factors: (a) reliability, test–retest and interrater reliability as applicable, as well as internal consistency; (b) distributions, floor and/or ceiling effects, and normality of distributions; (c) utility as a repeated measure, stability over time in the absence of intervention, or sensitivity to intervention associated change; (d) convergent and discriminant validity, relationship to social cognitive measures relative to other abilities and constructs; (e) relationship to functional outcomes; (f) practicality for administration; and (g) tolerability for patients. The eight included instruments were: Bell Lysaker Emotion Recognition Task (BLERT) and Penn Emotion Recognition Task (ER-40) assessing the emotional processing domain; Reading the Mind in the Eyes Test and The Hinting task assessing ToM; The Awareness of Social Inference Test (TASIT), assessing the emotional processing and ToM; Relationships Across Domains (RAD) assessing social perception; Ambiguous Intentions Hostility Questionnaire (AIHQ) and Trustworthiness Task assessing the attributional bias/style domain.

During the third phase of the SCOPE study, psychometric properties of all the above-mentioned eight instruments have been evaluated in a sample of 179 stable outpatients with schizophrenia and 104 healthy controls. Finally, during the fourth and fifth phases of the SCOPE study, psychometric properties of the eight instruments were evaluated in a sample of 218 stable outpatients with schizophrenia and 154 healthy controls. These instruments included those that were classified during the third phase as adequate for the use in clinical trials (BLERT and Hinting task), or acceptable with modifications (ER-40, Eyes task, and TASIT), and three new measures, the Mini Profile of Nonverbal Sensitivity (MiniPONS) and the Social Attribution Task-Multiple Choice (SAT-MC), assessing social perception; the Intentional Bias Task (IBT), assessing attributional bias/style.

In the following paragraphs, we will illustrate all the above-mentioned instruments, dividing them on the basis of the social cognition domain explored.

### Emotional processing

*The BLERT* [174] (Supplementary Table 8) measures the ability of a person to identify affect cues. It is an audio-visual task designed to elicit a person’s ability to properly discriminate seven emotional states (happiness, sadness, fear, disgust, surprise, anger, or no emotion) expressed by facial, voice-tonal, and upper-body movement of a male actor during 21 10-s video clips. BLERT has a short administration time of about 6–8 min and good psychometric properties in line with the key criteria individuated by the SCOPE initiative (Supplementary Table 9). In particular, BLERT showed good test–retest reliability and internal consistency, limited potential for floor/ceiling effects, good utility as a repeated measure, good convergent and discriminant validity, good practicality for administration, tolerability for patients, and good sensitivity to differentiate between patients and healthy controls [37,169,170,175]. BLERT also showed significant correlations with measures of functioning, functional capacity, and social competence [37,169,170] and significantly predicted real-world functioning [170]. The modified version of the BLERT, which included response time and confidence ratings, tested by Pinkham et al. [169] had psychometric properties similar to the original version, although it showed greater practice effects and reduced criterion validity as compared to SCOPE phase 3. Of note, one of the weaknesses of BLERT is that the same Caucasian male actor is used for all stimuli, which might lead to a non-appropriate use of this instrument in non-Caucasian populations [176].

*The Penn Emotion Recognition Task* (ER-40) [177] (Supplementary Table 8) assesses facial emotion recognition ability. It includes 40 color photographs of faces depicting a given emotion (i.e., happiness, sadness, anger, or fear) or a neutral expression. Stimuli are balanced for poser’s gender, age, and ethnicity, and for each emotion category. Participants are instructed to examine a series of faces and identify the expressed emotion from five possible choices. ER-40 has a short administration time of about 3–4 min and good psychometric properties in line with key criteria defined by the SCOPE initiative (Supplementary Table 9). In particular, studies have reported good test–retest reliability and internal consistency, limited potential for floor/ceiling effects, a good utility as a repeated measure, practicality for administration and tolerability for patients, good sensitivity to differentiate between patients and healthy controls, and good convergent validity with modest discriminant validity [37,169,170,175,178]. ER-40 also showed significant correlations with measures of functional capacity [169,170] and social competence [169,170,179], but not of real-life functioning [170], at odds with what observed for BLERT [169,170]. The modified version of the ER-40, which included response time and confidence ratings, tested by Pinkham et al. had the advantage that confidence ratings correlated with measures of real-life functioning [169]. In addition, ER-40 accuracy and response time emerged as unique significant predictors of social competence, even when controlling for all other cognitive and social cognitive variables [169]. However, further studies are needed to confirm the associations between the ER-40-modified version and real-life functioning.

*The* MSCEIT [171] (Supplementary Table 8) is a 141-item scale, made up of eight tasks, measuring four branches of emotional intelligence (EI): perceiving emotions, using emotions to facilitate thoughts, understanding emotions, and managing emotions (ME). MSCEIT-ME represents to date the only measure that assesses emotional regulation, a subdomain of the emotional processing domain. Each subscale consists of two tasks. The MSCEIT can be scored at three levels: (a) an overall score reflecting a general level of EI; (b) two area scores, experiencing EI and strategic EI; and (c) four branch scores (each measured by two subtests). Each of these scores is obtained through two scoring criteria: expert scoring criterion and consensus scoring criterion. The expert scoring criterion is based on responses to the test items from 21 members of the International Society for Research on Emotion. The consensus scoring criterion is based on the responses to the test items from a large and heterogeneous standardization sample of over 5,000 subjects. Actually, MSCEIT scores are computed using the general consensus approach based on a large community sample rather than the expert rating approach. However, the interpretation of the consensus-based scoring method is controversial as it is unclear whether the scores reflect true variations in EI [180]. MSCEIT has been shown to have a long administration time of about 35 min and modest to good psychometric properties [173–175]. In particular, good internal consistency has been reported for MSCEIT branch scores, as well as for MSCEIT total score [173,175,181], good test–retest reliability for MSCEIT branches 1 and 4 [84,109], good sensitivity to differentiate between patients and healthy controls [173,175], and good tolerability and good discriminant validity [173]. A low degree of convergent validity, as well as practicality and utility as a repeated measure, have also been reported [173], [175]. Furthermore, MSCEIT total score and branch scores demonstrated modes correlations with measures of functioning [73,109,173,182,183].

### Theory of mind

*The Hinting task* [184] (Supplementary Table 8) was devised to test the ability of subjects to infer the true intent of indirect speech utterances. The task comprises 10 short passages read aloud presenting an interaction between two characters and involving one of the characters dropping a hint. The participants are then asked to say what the character dropping the hint intended. The Hinting task has a short administration time of about 5–7 min and overall good psychometric properties (Supplementary Table 9). In particular, it has good internal consistency and test–retest reliability, small practice effects, limited potential for floor/ceiling effects [37,169,170], good practicality for administration and tolerability for patients [169,170], good sensitivity to differentiate between patients and healthy controls [170], and good convergent but weak discriminant validity [37]. The Hinting task also demonstrated a correlation with measures of functioning, functional capacity, and social competence [37,169,170]; furthermore, it was the only significant predictor or showed significant incremental validity in the prediction of functional capacity and social competence [169,170]. However, a recent study [175], investigating psychometric properties of different social cognitive measures in an Asian sample, found the Hinting task less favorable psychometric properties in terms of internal consistency in comparison with previous studies. Probably, this was due to the cultural sensitivity of the task that examines inferential ability through the presentation of short vignettes, which might require cultural adaptation.

*The Reading the Mind in the Eyes Test (Eyes Test)* [185] (Supplementary Table 8) measures the capacity to discriminate the mental state of others from expressions in the eye region of the face. It includes 36 photographs of male and female eyes, rather than the whole face, depicting emotional states. For each photograph, participants are asked to choose the emotional state that best describes the eyes, choosing among four possible emotions. Each stimulus is presented with four response options. Participants are awarded one point for each correct item. It has a short administration time and adequate psychometric properties (Supplementary Table 9): good internal consistency and test–retest reliability; good utility as a repeated measure; good convergent and good to modest discriminant validity; practicality for administration, tolerability for patients, and good sensitivity to differentiate between patients and healthy controls [37,169,170]. The Eyes Task also was significantly correlated with measures of functional capacity [169,170] and social competence [169,170,186], but not of real-life functioning [170]. The modified version of the Eyes Task, which included definitions of terms used, tested by Pinkham et al. [169], in order to reduce the dependence of performance on vocabulary, did not ameliorate the lack of relationship with measures of real-life functioning [169]. Overall, the Eyes Task represents a promising instrument to assess the ToM domain. However, due to some limitations in its psychometric properties, even in the modified versions of this instrument, further validation studies are needed.

### Emotional processing and ToM

*TASIT* [187] (Supplementary Table 8) consists of videotaped vignettes of everyday social interactions and includes three parts, each with alternative forms (form A and B): *Part I: The Emotion Evaluation Test*, which evaluates the ability to recognize the basic emotions expressed by others in 28 video sequences; the subject is asked to identify the emotion expressed by a character, choosing from 7 options (surprise, happiness, anger, sadness, anxiety, disgust, neutral); *Part 2: Social Inference—Minimal (SI-M),* which includes 15 vignettes that represent dialogues between two actors and assess comprehension of sincere versus sarcastic exchanges; *Part 3: Social Inference—Enriched (SI-E),* which includes 16 vignettes that provide additional information before or after the dialogue of interest to “set the scene” and assess lies versus sarcasm. The administration time of the entire test is about 30–45 min; Part III, which is used to detect lies and sarcasm, lasts about 17–19 min. The total score and the score for part III have good internal consistency and test–retest reliability [69,169,187]. All three parts showed good convergent and weak discriminant validity, good tolerability for patients but weak practicality due to the administration time, and significant practice effect [37,169,170,175] (Supplementary Table 9). Part III has shown correlations with measures of functional capacity [17,21,72,106,169,170,188], social competence [169,170], and real-life functioning [17,67,72,106,170,188,189]. The modified version of the TASIT-III, which includes response time and counterbalanced administration of test forms across visits, tested by Pinkham et al., has psychometric properties similar to the original version, although response time scores do not correlate with measures of real-life functioning [169] (Supplementary Table 9). Counterbalancing form administration reduced the discrepancy between forms noted in phase 3 of the SCOPE study [169]. In a large sample of subjects with schizophrenia, recruited within a large multicenter study of the Italian Network for Research on Psychoses (NIRP), it has been demonstrated that social cognition, whose assessment included the TASIT, as well as the MSCEIT and FEIT, was correlated with real-life functioning and functional capacity at baseline [17,188] and at 4-year follow-up [21]. In the same sample, through a network analysis, the authors found that TASIT-1 was connected to all the other social cognition nodes and bridged the social cognition domain with the functional capacity node and, through the latter one, with the real-life functioning nodes [72]. These results were also confirmed at 4-year follow-up [106]. Different factors might play a role in discrepancies of results concerning the presence or the absence of association between TASIT and real-life functioning. In particular, the smaller sample size, a milder impairment of social cognition, or a reduced variance in real-life functioning observed in Pinkham et al.’s studies [169,170], as compared to other studies [17,18,21,72,106], might account for the lack of associations between TASIT and real-life functioning [69,169]. Therefore, further studies including large populations of subjects with schizophrenia are needed.

### Social perception

*RAD* [190] (Supplementary Table 8) is a 75-item paper-and-pencil measure of competence in relationship perception. The content and format of RAD are based on relational model theory, which proposes that individuals use their implicit knowledge of four relational models to understand social relationships and predict the behavior of others: (a) communal sharing, (b) authority ranking, (c) equality matching, and (d) market pricing. The administration time is estimated at 35 min (16 min for the abbreviated version). The test has weak psychometric properties [37,175] (Supplementary Table 9), in particular, considerable floor effects, low practicality, and poor tolerability by patients [170].

*The SAT-MC* [191] (eTable 8). Participants viewed a short animation of geometric shapes enacting a social drama. The animation was shown twice, and participants then answered 19 multiple-choice questions about what happened. This instrument showed weak psychometric properties, in particular, poor test–retest reliability, a great floor effect, a modest relationship with measures of functional capacity and social competence, and no relationship with measures of functioning [169] (Supplementary Table 9).

*The MiniPONS* [192] (Supplementary Table 8) tests accuracy in decoding interpersonal cues (face, body, and voice tone). Participants were presented 64 two-s auditory or visual segments of a Caucasian female exhibiting facial expressions, voice intonations, and/or gestures and had to choose which of two behavioral labels best described the situation. This instrument showed weak psychometric properties, in particular a great floor effect, a poor tolerability, a modest relationship with measures of functional capacity and social competence, and no relationship with measures of real-life functioning [169,175] (Supplementary Table 9).

At the present time, given the low psychometric properties of tasks assessing social perception in schizophrenia, and the limited amount of evidence, further studies are needed in order to validate a measure to assess this social cognition domain.

### Attributional bias/style

*The AIHQ* [193] (Supplementary Table 8) evaluates hostile social cognitive biases. Participants read five hypothetical negative social situations with ambiguous causes and record a reason why the scenario occurred. For each situation, participants rate the intentionality of the other’s action, how angry it would make the participant feel, and how much s/he would blame the other. These three items are summed up for an overall blame score with higher scores indicating greater blame, perceived intention, and anger. Additionally, participants provide two open-ended responses: an explanation of why the event occurred, and what they would do in response to the event. These open-ended questions are evaluated by two independent raters according to the extent to which the participant interpreted the situation in a hostile manner (hostility bias, rating a hostility index) and the extent to which the individual reports aggression in her or his behavioral response (aggression bias, rating an aggression index). AIHQ has a short administration time of about 5–7 min. It shows good internal consistency [37] but a low test–retest reliability (with the exception of the blame score) [170]. The instrument has low sensitivity in differentiating between patients and healthy controls [170], low to moderate degree of utility as a repeated measure [170,194–197], and moderate to not significant convergent validity scores [67,198]. However, the study of Lim et al. [175] found acceptable test–retest reliability for the AIHQ Hostility Bias subscale (AIHQ-HB) in the controls and slightly poorer test–retest for the AIHQ Aggression Bias subscale (AIHQ-AB) in patients. A possible reason for different results between Pinkham et al. [170] and Lim et al. [175] is that the SCOPE [170] only administered items on ambiguous scenarios (Pinkham et al. [170]), while Lim et al. [175] administered the full AIHQ, which consisted of vignettes on ambiguous, accidental, and intentional scenarios. Further evaluation of this aspect is needed. Furthermore, AIHQ did not correlate with measures of real-life functioning, functional capacity, and social competence [37,170] (Supplementary Table 9).

*The Trustworthiness task* [199]. Participants are shown 100 (or 42 in the abbreviated version of the task) faces of unfamiliar people, showing ethnically diverse males and females in natural poses, and are asked to judge how much they would trust the person by providing a rating on a 7-point scale (Supplementary Table 8). This scale has often been used in patients with brain injury and with autism spectrum disorder [37], and rarely in subjects with schizophrenia, in which it showed weak psychometric properties (Supplementary Table 9), in particular, low convergent validity, low sensitivity in discriminating patients from healthy controls, and no relationship with measures of real-life functioning, functional capacity, and social competence [37,54,170]. On the other hand, the instrument showed good reliability, utility as a repeated measure, practicality of administration, and tolerability for patients [170,200] (Supplementary Table 9).

*The IBT* [201] (Supplementary Table 8) assesses the tendency to attribute intentionality to the actions of others. Participants read 24 brief descriptions of ambiguous actions and quickly indicate whether that action occurred “on purpose” or “by accident.”. IBT showed acceptable psychometric properties (good internal consistency, utility as a repeated measure, relationship with functional capacity and real-life functioning, tolerability and practicality, sensitivity in differentiating patients from controls) [169,202], but some limitations, in particular, low test–retest reliability scores, and high rate of missing data due to limited response times [169] (Supplementary Table 9). Even if IBT represents a promising instrument to evaluate attributional style/bias, the relevant literature is scant at the present time. Therefore, this instrument needs more validation studies to be recommended for assessing impairment in attributional style/bias in subjects with schizophrenia.

At the present time, given the low psychometric properties of tasks assessing attributional bias/style in schizophrenia, and the small amount of evidence, further studies are needed in order to validate a measure to assess this social cognition domain.

In conclusion, to date, different instruments are available to assess emotional processing and ToM in subjects with schizophrenia, while no validated instrument might be indicated in assessing social perception and attributional bias/style. In addition, unfortunately, a unified battery assessing all social cognition domains impaired in subjects with schizophrenia is not available. Therefore, much more effort involving experts in this field is needed to identify and develop broader measures of social cognition relevant to schizophrenia. The cross-cultural validity of the different instruments also requires scrutiny, in light of the considerable influence of cultural factors on social interactions and difficulties arising from transferring subtle nuances of social communication from one language to the other. In the Italian version of the TASIT, adopted in the studies of the Italian Network for Research on Psychoses [17,186], the videotaped vignettes of the test had to be dubbed in Italian by a prestigious society in the field of film industry, as the job requires the professional expertise of actors and of course, entails not negligible costs.

A summary of the characteristics of social cognition assessment tools is provided in Supplementary Table 8.

### Recommendations

Considering the available literature, the working group elaborated the following recommendations:

**Table:** 

Grade	Recommendation 13 (based on studies included in Supplementary Table 9)
B	Due to their good psychometric properties, BLERT and Hinting task should be used for the assessment of the social cognition domains emotional processing and ToM, respectively

**Table:** 

Grade	Recommendation 14 (based on studies included in Supplementary Table 9)
C	Due to its psychometric properties, TASIT can be used for the assessment of the emotional processing and ToM domains

**Table:** 

Grade	Recommendation 15 (based on studies included in Supplementary Table 9)
B	Due to their inadequate psychometric properties, RAD, MiniPONS, and SAT-MC should not be used to assess social perception impairments in subjects with schizophrenia

**Table:** 

Grade	Recommendation 16 (based on studies included in Supplementary Table 9)
B	Due to their inadequate psychometric properties, AIHQ and the Trustworthiness Task should not be used to assess attributional bias/style in subjects with schizophrenia

## Assessment of Cognitive Impairment in Early Intervention Settings

Patients with FEP and UHR subjects show widespread and persistent cognitive impairments [11,203,204] that can impede functional recovery. Indeed, cognitive deficits have consistently been reported to be associated significantly with the functional prognosis of UHR and FEP patients in both cross-sectional and longitudinal studies [19,205–216].

Cognitive assessment forms an important part of the assessment battery used in early intervention facilities as the cognitive deficits in themselves may be the focus of targeted interventions, but furthermore, as the cognitive assessment allows for taking the cognitive deficits into account when offering therapy in early intervention settings; that is by tailoring the therapy, so it fosters areas of growth and does not exceed the individual’s cognitive capacity. Additionally, cognitive assessment is of relevance when facilitating and supporting the patient in his/her vocational pursuits. A comprehensive cognitive assessment should comprise both neurocognitive and social cognitive deficits.

### Assessment of neurocognitive deficits

The assessment of neurocognitive impairments has a longer tradition than social cognitive assessments, and thus the selection of neurocognitive batteries and the literature on the topic are more elaborated and have already been described in previous sections [7,9–11,14].

Reviewing the literature on cognitive assessment batteries used in cognitive remediation trials in early phases of psychoses revealed that many of the studies used an assessment battery that adhered to the cognitive domains defined in the MATRICS initiative [109] with 3 out of 13 studies specifically using the MCCB [217–219]. The additional studies used a variety of tests assessing selected or multiple aspects of cognitive function with the domains of memory functions and executive functions being the most commonly assessed [220–227]. Regarding cognitive remediation trials in the UHR subjects, fewer studies have been conducted [228]. To date, evidence from only three RCTs and three cohort studies are available. Two of the three RCTs and one cohort study used the MCCB [229–231], while the remaining studies used tests predominantly indexing processing speed and verbal learning [232–234].

To achieve a comprehensive understanding of the potential areas of deficits, and strengths, cognitive assessments in early intervention facilities should comprise a battery that covers the six core neurocognitive domains and not merely providing a neurocognitive global score. The findings from cognition studies on FEPs and UHR individuals indicate the utility of a standardized cognitive assessment battery such as the MCCB to be used in early intervention facilities. An alternative shorter battery that can be used in these facilities is the BACS, which covers four of the six MATRICS-defined neurocognitive domains. Domain scores and a neurocognitive composite score can be derived from the BACS battery. The BACS battery and BACS subtests have also shown efficacy in detecting neurocognitive deficits in patients with a first-episode psychosis [235,236] and UHR individuals [233,237,238]. Both the MCCB and the BACS have exhibited robust correlations with functional measures [109,121]. The CANTAB [239] offers a selection of cognitive tests, representing the core neurocognitive domains, and has proven sensitive to cognitive deficits in patients with a FEP [223] and UHR individuals [240,241]. Hence, the CANTAB tests may show utility to form a neurocognitive assessment battery, with the limitation already discussed, that is, the included tests were developed for elderly patients with dementia and might present a ceiling effect especially in young FEP or UHR individuals.

Finally, the Measure of Insight into Cognition-Self Report (MIC-SR) represents a measure of self-perceived cognitive ability that has been recently found to be related with overall functioning and QoL in UHR individuals [242].

In conclusion, assessing neurocognitive deficits in early intervention facilities is recommended to be in keeping with the MATRICS-designated separate cognitive domains. The standardized neurocognitive batteries the MCCB and the BACS constitute psychometrically sound instruments shown to be sensitive to the neurocognitive deficits in the UHR population and in FEP and may therefore be the preferred choices. However, normative data for the adolescent population should be collected to improve the accuracy of the batteries in detecting neurocognitive impairment in UHR and the FEP subjects under 18 and to help diagnostic decision-making in clinical settings. The CANTAB may offer supplementary tests to index the range of neurocognitive deficits in the putative prodromal and early stages of psychosis, but with the limitation reported above.

### Recommendations

Considering the available literature, the working group elaborated the following recommendations:

**Table:** 

Grade	Recommendation 17
A	Assessing neurocognitive performance is recommended in early intervention facilities both in FEP and UHR subjects

**Table:** 

Grade	Recommendation 18
B	Standardized cognitive batteries such as MCCB and BACS should be used to assess neurocognitive performance in early intervention settings as they represent valid instruments for the adult population

**Table:** 

Grade	Recommendation 19
C	Standardized cognitive batteries such as MCCB and BACS can be used to assess neurocognitive performance in early intervention settings in adolescents, but normative data need to be collected

#### Assessment of social cognitive deficits

Social cognition is a relatively young research field that has suffered a significant problem of lacking social cognitive tests with good psychometric properties. The BLERT [243], Hinting task [235,243–246], ER-40 [247], Reading the Mind in the Eyes task [243,248–250], and TASIT [84,235] have been used in studies with FEP patients and found to be effective in detecting social cognitive deficits in this population. Likewise, the TASIT [73,238,251] and the ER-40 [251] have been found to be efficient in detecting deficits in mental attributions and emotion recognition in UHR patients. Additionally, the Reading the Minds in the Eyes (Eyes) task has been used in UHR studies [206,248], but the test’s ability to discriminate between UHR individuals and healthy controls still need to be established [248]. Consequently, the UHR research is in vital need of further validation of social cognitive tests.

Considering the subjective experience of social cognition, only preliminary findings are currently available in FEP and UHR subjects [252].

In conclusion, there is currently no standardized social cognitive test battery available to be recommended for use in early intervention settings. Based on the findings from the comprehensive SCOPE test evaluation, the best available social cognitive measures to be used in psychosis research are the BLERT, the Hinting task, and the ER-40 which have proven strong psychometric properties. The Eyes, TASIT, and IBT have acceptable psychometric properties and may show utility as measures of ToM and attributional style. These six tests are therefore suitable for use in early intervention settings, but the psychometric limitations to the latter three measures must be kept in mind. Furthermore, it must be recognized that the SCOPE initiative validated the tests for use in patients with established psychosis, and while they have shown efficacy in detecting social cognitive deficits in FEP and UHR populations, the psychometric properties of the tests may not apply as consistently for the FEP and UHR populations (e.g., they may show ceiling effects). This underscores a need for the development and validation of social cognitive tests specifically for use in the putative prodromal state of psychosis and FEP to capture the potential subtleties of social cognitive deficits occurring in these phases.

### Recommendations

Considering the available literature, the working group elaborated the following recommendations:

**Table:** 

Grade	Recommendation 20
A	Assessing social cognition performance is recommended in early intervention facilities both in FEP and UHR subjects

**Table:** 

Grade	Recommendation 21
B	BLERT, the Hinting task, and the ER-40 should be recommended in early intervention settings for their good psychometric properties in adult FEP or UHR subjects

**Table:** 

Grade	Recommendation 22
C	Due to their psychometric properties, the Eyes and TASIT can be used for the assessment of the emotional processing and ToM domains in early intervention settings in adult FEP or UHR subjects

### Discussion

The aim of the present work is to provide a comprehensive and detailed overview of cognitive impairment in schizophrenia and to give evidence-based recommendations for its assessment both in research settings and in real-world clinical practice. In fact, assessment generally represents the first step for improvement, both for implementing currently available evidence-based treatment options and to appropriately develop new and more focused pharmaco- and psychotherapeutic remedies, including rehabilitation, aiming for precision psychiatry [253].

Research supports that cognitive impairment is a core feature of schizophrenia and is not just the result of other symptomatological dimensions or the consequence of current treatments [5,254]. Several meta-analyses and systematic reviews consistently demonstrated that at least 80% of subjects with schizophrenia present a mild to severe impairment in different cognitive domains, involving both neurocognitive and social cognitive domains [3–9,17]. In line with the NIMH consensus conference and major systematic reviews, six neurocognitive domains as well as four social cognition domains are impaired in schizophrenia [36,77,109,135,255]. The key neurocognitive domains include speed of processing, attention/vigilance, working memory, verbal learning and memory, visuospatial learning and memory, reasoning, and problem solving, while the key social cognitive domains include emotional processing, social perception, ToM, and attributional bias [19,169,170,255]. Cognitive impairment is present since the first episode of psychosis and also in subjects at risk for psychosis [3,6,11,204], as well as in non-affected first-degree relatives of people with schizophrenia [12,13]. The overall magnitude and pattern of cognitive impairment remain substantially stable over the course of schizophrenia [14]. The deficits in multiple neurocognitive domains seem to affect real-life functioning more than negative and positive symptoms [15–18]. The impact of neurocognitive deficits on real-life functioning seems to be mediated at least in part by social cognition [15,17–21,256,257]. However, despite their crucial role in determining a worse real-world functioning, cognitive deficits are often not assessed in clinical practice.

The fifth edition of DSM includes cognition as a domain that needs to be evaluated by clinicians in the course of a diagnostic assessment [258]. In the last decades, the identification of cognitive impairment in schizophrenia is improved and studies reviewed in this paper provide evidence that cognition can be reliably assessed using appropriate instruments. The present guidance for the optimal assessment of cognitive symptoms in schizophrenia is based on expert consensus and systematic reviews [36,37,77,105,109,135,255]. Based on the reviewed evidence, we recommend a systematic assessment of the neurocognitive domains identified by the MATRICS initiative in subjects with schizophrenia, in all phases of the disorder, as well as in subjects at risk to develop psychosis.

This assessment should be conducted as early as possible, also in the perspective of developing a personalized treatment program [253], and repeated to observe potential changes, particularly following interventions that specifically target cognitive performance in order to assess their effectiveness and document potential improvements.

However, it must also be considered that a thorough assessment of the different domains of cognition requires human and technical resources, which are globally—and even in many European countries—either not available or neglected; this issue has been particularly problematic also in wealthier countries given the recent difficulties faced by mental health services linked to the COVID-19 pandemic [259–262]. Therefore, the profile of cognitive impairment of many patients currently goes unrecognized [263]. Moreover, the still restricted treatment options available for cognitive impairment, including pharmacotherapy effectiveness, may not balance favorably with an extensive and elaborate assessment.

How does that translate into current clinical practice? The DSM-5 provides specifiers for clinician-rated symptom severity profiling in psychotic disorders, including “impaired cognition”—in the chapter Assessment Measures of Section III Emerging Measures and Models [258] hence not in Section II on Diagnostic Criteria and Codes of Mental Disorders. The ICD-11 [264] in its chapter on schizophrenia or other primary psychotic disorders with pre-coordinated course specifiers provides somewhat similar symptom severity specifiers, including cognitive impairment with operationalizations in the “Clinical Descriptions and Diagnostic Guidelines” (now “Requirements” [265]), which can be directly post-coordinated with the respective psychotic disorders. The expected global mandatory assessment of cognitive impairment represents a step forward to at least recognize and judge the severity of cognitive impairment compared to its full neglect, which still represents an often-observed issue [263]. In fact, this development in both DSM-5 and ICD-11 points in the right direction of the current global clinical situation in which many countries or institutions do not have even the option and the resources to perform detailed assessments, let alone have specific and effective treatments available.

In this perspective, providing some kind of evaluation of cognitive performance for people living with schizophrenia in everyday, real-world clinical practice, even with simpler assessment tools that have lower levels of recommendation, or with interview-based instruments, or with clinical observation,-following symptoms severity specifiers, represents a substantially better alternative compared to leaving cognition completely unassessed, particularly if available resources are limited.

Likewise, providing an assessment of cognitive performance at least once in the lifetime of the patient is considered preferable than never performing any kind of evaluation.

As to the factor structure of neurocognition and social cognition to be used in clinical trials, no recommendation is deemed appropriate by the EPA Guidance Group on Cognitive Impairment on the basis of the available evidence. Further studies are needed to validate the six neurocognitive domains identified by the MATRICS initiative [36], and the four social cognitive subdomains identified by the SCOPE initiative [37]. Validation should be based on patterns of differential associations with functional outcome measures or pathophysiological mechanisms or differential sensitivity to pharmacological and non-pharmacological interventions. In recent years, the assessment of cognitive functions progressed with the development of validated, reliable, and comprehensive clinician-rated neuropsychological batteries and self-rated instruments with better assessment of experiential cognitive symptoms. This guidance paper provides evidence-based recommendations for using both observer reports and self-reports of cognitive performance, as they give additive and complementary information [95]. In particular, self-report of neurocognition predicts everyday activities and self-report of social cognition predicts interpersonal relationships [94]. In this context, MCCB should be used because it covers the seven cognitive domains identified by the MATRICS Consensus Initiative, BACS could be used for its short time of administration, and SCIP should be used as a screening tool. We also advise a carefully social cognitive assessment, taking into account the following subdomains: emotion processing, social perception, and ToM. Due to their good psychometric properties, BLERT and Hinting tasks should be used for emotional processing and ToM assessment, respectively, while TASIT could be used for emotional processing and ToM assessment. Furthermore, it is essential to highlight that the MATRICS initiative also adopted interview-based cognitive assessment tools as a co-primary measure [148]. Interview-based measures are designed to evaluate the impact of cognitive impairment on functioning and might capture better the clinical meaning of changes over time or following pharmacological or psychosocial interventions [150,151,161]. Due to their good psychometric properties and face validity, we recommend SCoRS or CAI as co-primary measures in the assessment of cognitive impairment of subjects with schizophrenia in routine clinical contexts and clinical trials.

It should also be noted that, when performing a thorough assessment of cognitive performance, interviews should also be conducted with caregivers and family members. They could provide further valuable insight regarding the patient’s cognitive functioning, as well as relevant details regarding the cognitive strategies and mechanisms adopted in real-world situations in daily life. Obtaining a clear overview of the patient’s cognitive strengths and weaknesses, in fact, represents a valuable asset when devising personalized treatment programs [253, 266].

The guidance also provides a systematic review of the state of the art of assessment in FEP and in individuals at risk for psychosis, highlighting the need to extending to the early stages of schizophrenia the use of a comprehensive cognitive assessment battery and further development of these assessment tools in the prodromal phases, and in subjects at risk.

As regards the limitations of the present guidance paper, no manual and non-systematic literature search was conducted on additional databases. However, a systematic search comprising three different electronic databases can be considered an accurate literature search [267].

Only the results of works published in English language were reported in the guidance; however, this represents a source of bias in systematic literature that is often considered of small effect [268, 269].

Future research perspectives include different areas of interest, but a number of topics of relevance emerged as requiring further studies. In particular, future research should focus on a better understanding of the factorial structure of cognitive deficits in schizophrenia, better exploring the relationship between deficits in neurocognitive and social cognition performance and both objective and subjective QoL, and developing valid and reliable tools to assess attributional style bias and social perception.

A summary of the reported findings and of the key points discussed in the guidance, providing several take-home messages, is provided in Box 2.

Box 2. Summary and take-home messages.

- *Neurocognitive and social cognition performance should always be carefully assessed in people living with schizophrenia and with clinically high-risk states, considering the important negative effects of cognitive deficits on functional outcomes and QoL.*
- *This assessment should be performed not only in the perspective of better characterizing the patient but also to help in the development of personalized treatment and rehabilitation programs.*
- *Cognitive performance should be assessed at least once in the lifetime of the patient, and optimally several times, in different phases of the illness and at the start and at the completion of dedicated treatment programs.*
- *The MCCB represents the most appropriate and complete validated tool that is currently available to assess neurocognitive performance in people living with schizophrenia and with clinically high-risk states.*
- *The BACS could be used as an alternative for its short time of administration.*
- *The SCIP could be used as a screening instrument.*
- *Interview-based instruments (SCoRS, CAI) can be used to obtain useful supplemental information.*
- *Social cognition performance should be assessed with the BLERT and the Hinting task for the emotional processing and ToM domains, respectively.*
- *The TASIT can be used to obtain useful additional information on both domains.*
- *For other domains of social cognition, no available test has sufficiently reliable psychometric proprieties to be recommended.*
- *In contexts with limited or very limited available resources, providing any type of cognitive assessment is better than providing no cognitive assessment at all.*

The construct of schizophrenia, even after about 110 years since its origin [1] and despite many modifications, has still made it into the contemporary classification systems, on the basis of which most of our evidence-based treatments have been clinically tested. However, although no paradigm shift has as yet happened in the classification of schizophrenia, research on transdiagnostic domain-specific deconstruction (Research Domains Criteria or RDoC) or reconstruction of hierarchical higher-order constructs (Hierarchical Taxonomy Of Psychopathology or HiTOP), including schizophrenia and other psychotic disorders, is fully underway [270-273].

Depending on whether and which new and practically useful entities will inherit which kind of domains of cognitive impairment in which new configuration, the kind and direction of development of new treatment concepts may also depend on using new methodological approaches for “precision psychiatry” [253].

Overall, the comprehensive review of the evidence and the elaboration of recommendations might contribute to advance the field, allowing a better cognitive assessment, avoiding overlaps with other psychopathological dimensions, such as negative symptoms, and antipsychotics side effects, such as extrapyramidal and secondary negative symptoms. Despite the clinical relevance of cognitive deficits identification in order to improve outcomes and to achieve recovery in people living with schizophrenia, the low grade of some recommendations reflects the still limited literature available in this field.

### Conclusions

Neurocognitive and social cognitive impairments in schizophrenia should be assessed in research settings and in clinical practice as they have an important impact on functional outcomes. This guidance paper aimed to promote the adoption of shared assessment protocols both in clinical trials and in real-world clinical setting, leading the way to further progress in the field of cognitive functions assessment and recognition in schizophrenia patients. Studies specifically aimed to assess neurocognitive and social cognitive functions at all the stages of schizophrenia should be carried out, in order to optimize the recognition and management of these symptoms, with the ultimate goal to improve outcomes and achieve recovery. Furthermore, sound methodological longitudinal studies aimed to assess the natural course and the trajectory of neurocognitive and social cognitive functions from the earliest phases of schizophrenia are extremely needed. Despite many steps forward are achieved in the last 15 years, much remains to be done, in order to reach a standardization of the neurocognitive and social cognitive construct and to identify effective strategies for cognitive deficits recognition in clinical practice. The dissemination of this guidance paper may promote the development of shared guidelines concerning the assessment of cognitive functions in schizophrenia, with the purpose to improve the quality of care and to obtain recovery.

## Data Availability

All the data that support the findings of this study are available within the article and its supplementary material.

## Supplementary material

For supplementary material accompanying this paper visit https://doi.org/10.1192/j.eurpsy.2022.2316. click here to view supplementary material
