# Supplementary material for: European Psychiatric Association guidance on assessment of cognitive impairment in schizophrenia
Source: Eur Psychiatry. 2022 Sep 5;65(1):e58. doi: 10.1192/j.eurpsy.2022.2316 (PMC9532219; doi:10.1192/j.eurpsy.2022.2316)
Supplement: Supplementary file 1 [file S0924933822023161sup001.docx]

**Table e1.** Cognitive impairment in subjects with schizophrenia. Systematic Reviews and Meta-Analyses.

| **Article** | **Type of study** | **Level** | **Assessment instrument or task / Explored domains** | **Sample size** | **Results (effect size/mean difference for the comparison between subjects with schizophrenia and healthy controls)** |
| --- | --- | --- | --- | --- | --- |
| Fioravanti et al., 2005 [1] | Meta-analysis | I | NART, WMS-R, CERAD neuropsychological battery, RCFT, VF task, BNT, the Reading Subtest of the WRAT, SCWT, WCST and TMT / IQ, memory, language, executive function (attention, response inhibition, working memory, abstract thinking, cognitive flexibility, set shifting), and attention. | 113 studies were included (4365 SCZ and 3429 HC) | Data based on IQ, memory, language, executive function and attention showed significant heterogeneity across studies (respectively *χ*2(46) = 185*.*07, *χ*2(57) = 303*.*23, *χ*2(35) =127*.*70, *χ*2(37) = 81*.*89, *χ*2(47) = 111*.*18, all *p* at least *<*.0001). However, in all five cognitive domains, analysis indicated a consistent trend for SCZ to perform worse than HC, with significant heterogeneity across studies. Memory (d = -0.83) shows the highest mean difference between SCZ and HC and language or IQ the lowest (d = -071). |
| Dickinson et al., 2007 [2] | Meta-analysis | I | Wechsler digit symbol test, or a close variant, such as a SDMT compared to other 36 cognitive tests grouped into 9 common cognitive domains / processing speed, episodic memory, executive functioning, working memory, sustained attention, motor speed, fluency, intellectual ability, problem solving | 37 studies were included (1961 SCZ and 1444 HC) | Combination of mean effect sizes across studies by means of a random effects model yielded a weighted mean effect for digit symbol coding of g = −1.57 (95% confidence interval, −1.66 to −1.48). This effect was similar to a grand mean effect of g = −0.98 and was significantly larger than effects for widely used measures of episodic memory, executive functioning, and working memory. |
| Sprong et al., 2007 [3] | Meta-analysis | I | False belief/deception task, intention-inferencing task, comprehension of irony and comprehension of metaphors / ToM | 29 studies were included (1518 SCZ)  To analyse the effect of specific clusters of symptoms on mentalising impairment, the symptom subgroups used by different research groups were divided into four categories: (a) symptoms of disorganisation. (b) no symptoms of disorganisation. (c) paranoid symptoms, (d) remitted patients. | This meta-analysis showed significant and stable mentalizing impairment in SCZ (d = –1.255). All symptom subgroups showed significant mentalising impairment, but participants with symptoms of disorganisation were significantly more impaired than the other subgroups (p < 0.01). |
| Hoekert et al., 2007 [4] | Meta-analysis | I | Emotional prosody task, different tasks depending on the study / Emotion processing (perception of emotional prosody and expression of emotional prosody) | 17 studies were included (sample not provided) | A significant and stable mean weighted effect size was found for the perception of emotional prosody, d = −1.24, 95% CI = −1.55 to −0.93. The effect was also found in the early stages of the illness. Expression of emotional prosody was also significantly impaired, d = −1.11, 95% CI = −1.78 to −0.43. |
| Bora et al., 2009 [5] | Meta-analysis | I | False-belief tasks, HINT, eyes task / ToM | 36 studies were included (1181 SCZ and 936 HC) | In "remitted" SCZ, the degree of ToM impairment was less pronounced than in non-remitted SCZ (d = 1.21) but it was still significant (d = 0.80). The persistence of ToM deficits in "remitted" patients suggests that there are trait-related mentalizing impairments in schizophrenia. |
| Forbes et al., 2009 [6] | Systematic Review  Meta-analysis | I | Twenty-one tests or subtests of phonological (verbal) working memory (e.g., digit span forward and backward, LNS, VLT) 11 tests or subtests of visuospatial working memory (e.g., visuospatial span – forwards and backwards, IVRT), four subtests of executive working memory (CANTAB SWM strategy score, Random Number/Letter Generation) / phonological (verbal) working memory, visuospatial working memory, executive working memory | 187 studies were included (sample not provided) | Deficits in SCZ were found for phonological working memory (for all tests, absolute effect sizes were from 0.55 to 1.41), visuospatial working memory (with absolute effect sizes ranging between 0.51 and 1.29) and executive working memory (effect sizes ranging between 0.73 and 0.92). |
| Doughty and Done, 2009 [7] | Systematic Review  Meta-analysis | I | Naming task, word picture matching task, verbal fluency task, semantic associations, semantic priming task, and categorisation task / Semantic memory | 91 studies were included (sample not provided) | Impairment is reported with large effect sizes for tests of naming (-1.45; 95% CI: -1.86, -1.04) and verbal fluency (-1.33; 95% CI: -1.15, -1.11), medium effect sizes for word picture matching (-0.58; 95% CI: -0.92, -0.24) and association (0.63; 95% CI: 0.003, 1.28) and small effect sizes for categorization (-0.49; 95% CI: -0.86, - 0.12) and priming tests (-0.02; 95% CI: -0.18, - 0.14). |
| Chan et al., 2010 [8] | Meta-analysis | I | Facial emotion perception task, Facial emotion identification task, facial emotion discrimination task / perception of facial emotion (emotion perception, part of emotion processing) | 28 studies were included (sample not provided) | SCZ have moderately to severely impaired perception of facial emotion (d = −0.85; 95% CI: −1.04, −0.66). |
| Knowles et al., 2010 [9] | Extended meta-analysis | I | Digit Symbol Coding, TMT-A and TMT-B, letter fluency, category fluency, WCST / Speed of processing, executive functions (attention, response inhibition, working memory, abstract thinking, cognitive flexibility, set shifting) | 36 studies (1,915 SCZ and 1,416 HC) were identified in the original meta-analysis by Dickinson et (2007) [2].  47 studies (4,135 SCZ and 2,292 HC) were entered into the extended meta-analysis. | The effect size of digit symbol coding tasks in schizophrenia is significantly larger than the effects of other cognitive measures. The largest effect size was for coding tasks (g = –1.50), followed by category fluency (g = –1.31). |
| Kohler et al., 2010 [10] | Meta-analysis | I | Identification tasks, differentiation tasks / facial emotion identification and differentiation (Emotion processing) | 86 studies were included (3822 SCZ) | Analysis of facial identification and differentiation impairment collapsed across the entire sample revealed a large overall effect size (d = -0.91, 95% CI = -0.97 < d < -0.84) that was significantly heterogeneous. |
| Westerhausen et al., 2011 [11] | Meta-analysis | I | Card and computerized Stroop Color-Word Interference Test / Executive functions (Cognitive inhibition) | 36 studies were included (1081 SCZ and 1026 HC) | SCZ exhibit an increased Stroop interference effect in response time (g = 0.43; 95% CI: 0.35, 0.52). Studies using the classical card version of the paradigm showed a significantly larger effect size than studies using a single-trial computerized version of the paradigm (g = 0.60 vs. g = 0.19). |
| Fioravanti et al., 2012 [12] | Meta-analysis | II | Several cognitive instruments (e.g., measures of memory efficiency, measures of memory functioning, digit span, verbal functioning [measures of fluency, naming tasks, etc.], WCST, Reaction Time) / Memory, global cognitive functioning, language, Executive function (attention, response inhibition, working memory, abstract thinking, cognitive flexibility, set shifting), attention | 247 studies were included (sample not provided) | Cognitive deficits are examined in 5 different domains: Memory (−1.22; 95% CI: -1.44, -1.01), Global cognitive ability (−0.96; 95% CI: -1.07, -0.85), Language (−0.99; 95% CI: -1.10, -0.87), Executive function (−1.10; 95% CI: -1.27, -0.92), Attention (0.99; 95% CI: 0.86, 1.12). There is a generalized impairment across cognitive domains in SCZ when compared to HC. |
| Savla et al., 2013 [13] | Update of a previous meta-analysis published in 2005 | I | Social cognition task (e.g., eyes task, HINT, Reading the Mind in the Eyes task, FEIT, MSCEIT, IPSAQ, PONS, SFRT) / Social Cognition Domains (ToM, social perception, social knowledge, attributional bias, emotion perception, emotion processing) | 112 studies were included (3908 SCZ and 3570 HC) | SCZ performed worse than HC across all domains (social perception, ToM, social knowledge, attributional bias, emotion processing) with large effects for social perception (g = 1.04), ToM (g = 0.96), emotion perception (g = 0.89), and overall emotion processing (g = 0.88), medium effect for social knowledge (g = 0.54) and negligible effect for attributional bias (g = -0.02 for Externalizing bias and g=-0.17 for Personalizing bias). |
| Bora et al., 2013 [14] | Systematic Review  Meta-analysis | I | HINT, eyes task, Cartoon, picture story task, Faux pas test, TASIT, CIT, MASC / ToM in first-episode psychosis, individuals at ultra-high risk for psychosis and their unaffected relatives | 21 studies were included (3005 first-episode, ultra-high risk individuals and unaffected relatives and 1351 HC) | ToM was substantially impaired in first-episode psychosis (d = 1.0) and this deficit was comparable to findings in chronic patients. ToM was also impaired in unaffected relatives (d = 0.37) and ultra-high risk subjects (d = 0.45) and performances of these groups were intermediate between first-episode psychosis and healthy controls. |
| Fatouros-Bergman et al., 2014 [15] | Meta-analysis | I | Several instruments among which CVLT, SVLT, HVLT-R, logical memory test from WMS, VF animal naming, TMT, LNS, Digit Symbol, Immediate Memory and Figure Recall Test from RBANS, Ax-CPT, PASAT, RVP, ToL, WMS, CANTAB, BVMT-R, PRM, RCFT / Verbal Memory, speed of Processing, working memory, attention, visual memory, executive functions (reasoning and problem solving) | 23 studies were included (1106 SCZ and 1385 HC) | Drug-naïve SCZ performed worse than HC in all cognitive domains analyzed: verbal memory (−1.03; 95% CI: −1.44, −0.63), speed of processing (−1.03; 95% CI: −1.23, −0.82), working memory (−0.97; 95% CI: −1.25, −0.69), attention (−0.80; 95% CI: −0.95, −0.65), visual memory (−0.78; 95% CI: −1.21, −0.34), executive functioning (−0.74; 95% CI: −0.85, −0.62). Verbal memory, speed of processing and working memory were three of the domains with the greatest impairments. |
| Lee et al, 2015 [16] | Meta-analysis | I | Several instruments or tasks among which Reading the mind in the eyes task, TASIT, HINT, VSIT, RAD, MSCEIT, AIHQ, IPSAQ, ANSIE, FEIT, FEDT, PERT, DANVA-2, Pictures of facial affect task, Facial affect recognition test / Social cognition domains (ToM, social perception, attributional bias, emotion processing) in prodromal psychosis | 20 studies were included (1229 subjects at clinical high risk and 825 HC) | Results indicated that individuals at clinical high risk exhibited significant impairments in all domains of social cognition compared with healthy controls. The overall effect size for social cognition was medium (g = −0.477). The largest effect size was identified for attributional bias (g = −0.708). A medium effect size was identified for emotion processing (g = −0.446) and ToM (g = −0.425), and small effects were identified for social perception (g = −0.383). |
| Berna et al., 2016 [17] | Meta-analysis | I | AMT, SDM, AMQ, AMI, AME, e-AGI; MRS / Autobiographical memory (memory specificity, richness of detail, and conscious recollection) | 20 studies were included (sample not provided) | This meta-analysis found moderate-to-large effect sizes with regard to the 3 parameters commonly used to assess autobiographical memory: memory specificity (g = −0.97), richness of detail (g = −1.40), and conscious recollection (g = −0.62). |
| Grimes et al., 2017 [18] | Meta-analysis | I | CVLT; WMS; RAVLT; HVLT; ROCFT; Benton Visual Retention Test / verbal and visual memory | 50 studies were included (sample not provided) | SCZ have severe verbal and visual memory impairments, which are not explained by task difficulty. For verbal memory domains, large effect sizes (ranging from 1.09 to 1.52) were found for all comparisons. For visual memory domains, large effect sizes (ranging from 0.99 to 1.31) were found for all comparisons. |
| Knapp et al., 2017 [19] | Meta-analysis | I | ToL, Tower of Hanoi, Stockings of Cambridge planning tasks / Executive functions (planning performance) | 31 studies were included (1377 SCZ and 1477 HC) | The findings demonstrated a planning deficit in SCZ (mean effect size: 0.67; 95% CI: 0.56, 0.78) that was moderated by task difficulty (number of moves required for a solution). |
| Watson et al., 2017 [20] | Meta-analysis | I | Stockings of Cambridge planning task / Executive functions (spatial planning) | 11 studies were included (662 with psychosis and 497 HC) | There were significant differences between people with non-affective psychosis and HC at all difficulty levels of performance. There was a very large effect size at the highest 5-level of difficulty (−1.61; 95% CI: −3.14, -0.08) and a moderate effect both at the medium 3-level of difficulty (−0.58; 95% CI: −0.75, −0.40) and across all difficulty levels (−0.66; 95% CI: −0.85, −0.46). |
| Thai et al., 2018 [21] | Meta-analysis | I | BADS six subtests: Rule Shift Cards; in Action Program, in Key Search, Temporal Judgment, in Zoo Map), MSET / Executive function (cognitive flexibility/set shifting, inhibition, novel problem solving, planning, monitoring own performance, estimation of time, planning a route, inhibition; organization) | 10 studies were included (375 SCZ and 541 HC) | Relative to HC, SCZ overall performed significantly worse in all subtests. Moderate effect sizes with regard to temporal estimation (−0.76; 95% CI: −1.18, −0.34) and strategy-forming (−0.85; 95% CI: −1.17, −0.55), while very large effect sizes were seen regarding complex forward planning (−1.52 95% CI: −2.23, −0.82), inhibition (−1.37; 95% CI: −2.01, −0.64), cognitive flexibility (−1.08; 95% CI: −1.57, −0.58) and novel problem solving (−1.16; 95% CI: −1.46, −0.87). The total composite showed the largest effect size (−1.70; 95%CI: −2.17, −1.24). |
| Zhang et al., 2019 [22] | Meta-analysis | I | MATRICS Consensus Cognitive Battery / MCCB composite score, speed of processing, attention/vigilance, visual learning, working memory, verbal learning, problem solving, social cognition | 56 studies were included (3167 FES and 3107 HC) | Compared with HC, FES showed impairment with large effect size in overall cognition (SMD = -1.60, 95% CI -1.82 to -1.38,  *I* ^2^ = 67%) and all seven cognitive domains, with the SMD ranging from -0.87 to -1.41.In nine MCCB subtests, patients with FES showed significant difference: Symbol Coding (SMD = −1.90), Trail Making Test (TMT) (SMD = −1.36), Continuous Performance Test-Identical Pairs (SMD = −1.33), Hopkins Verbal Learning Test (SMD = −1.24), Brief Visuospatial Memory Test (SMD = −1.18), Mazes (SMD = −1.16), Category Fluency (SMD = −1.01), Spatial Span (SMD = −0.69) and Mayer-Salovey-Caruso Emotional Intelligence Test (SMD = −0.38). |

**AIHQ**: Ambiguous Intentions Hostility Questionnaire; **AME**: Autobiographical Memory Enquiry; **AMI**: Autobiographical Memory Inventory; **AMQ**: Autobiographical Memory Questionnaire; **AMT**: Autobiographical Memory Test; **ANSIE**: Adult Nowicki Strickland Internal External locus of control scale; **BADS**: The Behavioural Assessment of Dysexecutive Syndrome; **BNT**: the Boston Naming Test; **CANTAB**: the Cambridge Neuropsychological Test Automated Battery; **BSRT**: Buschke Selective Reminding Test; **BVMT-R**: Brief Visuospatial Memory Test Revised; **CERAD**: The Consortium to Establish a Registry for Alzheimer’s Disease; **CI**: confidence interval; **CIT**: Character Intention Task; **CPT**: continuous performance test; **CVLT**: California Verbal Learning Test; **DANVA-2**: Diagnostic Analysis of Nonverbal Accuracy 2; **E-AGI**: Erweitertes Autobiographisches Gedächtnisinventar; **FEDT**, Face Emotion Discrimination Task; **FEIT**: The Face Emotion Identification Test; **FES**: First-episode of Schizophrenia; **HC**: healthy controls; **HINT**: Hinting Task; **HVLT-R**: Hopkins Verbal Learning Test—Revised; **IPSAQ**: The Internal, Personal, and Situational Attributions Questionnaire; **IQ**: intelligence quotient; **IVRT**: Immediate Visual Recall Tests; **LNS**: Letter number span; **MASC**: the Movie for the Assessment of Social Cognition; **MCCB**: MATRICS Consensus Cognitive Battery; **MRS**: Memory Rating Scale; **MSCEIT**: Mayer-Salovey-Caruso Emotional Intelligence Test; **MSET**: the Modified Six Elements Test; **NART**: The National Adult Reading Test; **PASAT**: Paced Auditory Serial Addition Test; **PERT**: Penn Emotion Recognition Test; **PONS**: Profile of Nonverbal Sensitivity; **PRM**: Pattern Recognition Memory test; **RAVLT**: Rey auditory-verbal learning test; **RBANS**: Repeatable Battery for the Assessment of Neuropsychological Status; **RAD**, Relationship Across the Domains test; **RCFT**: the Rey Complex Figure Test; **ROCFT**: Rey–Osterrieth Complex Figure Test; **RVP**: Rapid Visual Information Processing; **SCWT**: The Stroop-Color Word Test; **SCZ**: subjects with schizophrenia; **SDM**: Sparse distributed memory; **SDMT**: Symbol Digit Modalities Test; **SFRT**: the Situational Features Recognition Test; **SMD**: Standardised mean difference; **SVLT**: Seoul Verbal Learning Test **SWM**: social working memory; **TASIT**: The Awareness of Social Inference Test; **TMT**: the Trail Making Test; **ToL**: Tower of London; ToM: Theory of Mind; **VF**: Verbal Fluency; **VLT**: Verbal Learning Tests; **VSIT**, Video Social Inference Task; **WCST**: Wisconsin Card Sorting Test; **WM**: working memory; **WMS-R**: Wechsler Memory Scale Revised; **WRAT**: the Wide Range Achievement Test.

**Table e2**. Factor analytic studies on cognitive domains in subjects with schizophrenia.

| **Article** | **Type of study** | **Level** | **Assessment** | **Statistics** | **Sample size** | **Results** |
| --- | --- | --- | --- | --- | --- | --- |
| **Articles published before the MATRICS Consensus Initiative** | | | | | | |
| Mirsky, 1987 [23] | EFA | III | Digit Span, Arithmetic, Digit Symbol, Talland Letter Cancellation Test, Stroop test, TMT, WCST, X and AX CPT | PCA | 86 psychiatric patients | The results of the PCA indicated that the 10 test scores commonly considered to be measures of attention could best be characterized by a series of four factors. Factor 1 focuses aspect of attention; factor 2 can be designated a vigilance factor; factor 3 seems to be a numerical-mnemonic quality of attention; factor 4 regards the flexibility aspect of attention-the capacity to shift. |
| Kremen et al., 1992 [24] | EFA | III | TMT, Digit Symbol, Digit Span, WRAT-R Arithmetic, WMS Mental Control, WCST, auditory CPT, dichotic listening | PCA | 34 patients with major psychotic disorders | Four factors emerged that may be identified as: perceptual motor speed; mental control (numerical mnemonic); flexibility; and vigilance. |
| Allen et al., 1998 [25] | CFA | II | WAIS-R | χ2, χ2/df, GFI, AGFI, RMSR, NFI | 169 SCZ and WAIS-R standardization sample (N=250) | For both groups, all model fit indices used to determine model adequacy supported three-factor model composed of Verbal Comprehension, Perceptual Organization and Freedom from Distractibility factors. |
| Hobart et al., 1999 [26] | EFA | II | RBANS, WAIS-III Vocabulary, Picture Arrangement, and Comprehension, WMS-III Logical Memory I and II, Visual Reproduction I and II, Woodcock–Johnson Listening Comprehension, Stroop, Trial Making Test, Gordon CPT, WCST, Finger Tapping, Purdue Pegboard | PCA | 150 SCZ or schizoaffective disorder | EFA found six factors: intelligence, language and memory; attention (involving performance speed); a visual memory factor; a vigilance factor; an executive factor; a motor factor. |
| Kurtz et al., 2001 [27] | EFA | III | PCPT, GDS, RHY, WCST, TAP, JOLO, STEREO, BD, MAEVN, VOC | PCA | 39 SCZ and 39 HC | The three factors can be labeled as: General Intellectual, Executive functioning and Visual Vigilance. |
| Green et al., 2002, Biol Psychiatry [28] | EFA | III | DS-CPT, Span of Apprehension, spatial work and reference memory tests, FAS fluency, CVLT, Digit Span Distractibility, Pin Test, WCST, Block Design, TMT | PCA | 62 SCZ | The PCA yielded three orthogonal interpretable factors: a perceptual discrimination factor, a memory and verbal fluency factor, and an executive factor. |
| Friis et al., 2002 [29] | EFA | II | CVLT, BMT, TAP, WCST, COWA, TMT, DSDT, CPT-IP | PCA | 219 FE | Five dimensions have been identified: working memory, verbal learning, executive function, impulsivity, and motor speed. |
| Dickinson et al., 2004, Biol Psychiatry. [30] | CFA | III | 18 subtests of WAIS-III and WMS-III | Structural equation modeling; RMSEA | 97 SCZ or schizoaffective disorder and 87 HC | Approximately two thirds of the overall effect of a schizophrenia diagnosis on cognitive performance was mediated through a single common factor. |
| Gladsjo et al., 2004 [31] | CFA | II | WAIS-R subtests, TMT, Boston Naming Test, Letter Fluency, Story Memory, CVLT, Figure Memory, Grooved Pegboard, WCST, Booklet Category Test | χ2, χ2/df, RMR, GFI, AGFI, RMSEA, and Tanaka's version of the AIC | 209 SCZ and related psychotic disorders | A six-factor model with two pairs of correlated errors fit the neuropsychological data significantly better than competing models with fewer factors. The six factors included verbal crystallized, attention/working memory, verbal episodic memory, speed of information processing, visual episodic memory, and reasoning/problem solving. |
| Keefe et al., 2004 [32] | EFA | II | BACS Digit Sequencing, Symbol Coding, Tower of London, Token Motor Task, Verbal Fluency, Verbal Memory | PCA | 150 SCZ | PCA was completed to determine the factor structure of the BACS. The factor structure suggests a three-factor solution. Measures that emphasize motor speed and general cognitive functions load on the first factor; the memory and working memory measures load on the second factor, and executive function loads on the third factor. |
| **Factor analyses published after the MATRICS Consensus Initiative (Neurocognition)** | | | | | | |
| Dickinson et al., 2006 [33] | CFA | II | WCST, TMT-A, TMT-B, some subtest of WAIS, Symbol Cancellation Test, BDAE, WMS-R, CVLT, Line Orientation, MAE | χ2, GFI, RMSEA, BNFI, AIC | 148 SCZ and 157 HC | Main analyses examined the fit of a hierarchical 6-factor model, in which associations among the factors were assumed to reflect their strong shared relationship to a general cognitive ability factor. The model incorporated the factors of verbal comprehension, perceptual organization, verbal memory, spatial memory, processing speed, and executive/working memory. |
| Keefe et al., 2006 [34] | EFA  CFA | II | Controlled oral word association test, Controlled oral word association test, Grooved pegboard, digit symbol test, WCST, WISC, HVLT, Computerized test of visuospatial working memory, Letter–number sequencing test, CPT, FEIT | PCA  χ2 | 1493 SCZ | A PCA of the neurocognitive data from this study suggested that the battery yields a single principal component with all measures showing intercorrelations in the medium to large range (2- 3- and 4-component solutions were examined). CFA suggested that a hierarchical single factor model of five domain scores (Speed of Processing, Vigilance, Working Memory, Verbal Memory, Reasoning) provided the best fit. |
| Genderson et al., 2007 [35] | EFA  CFA | II | WAIS IQ, Zero/One/Two/Three-back test, WMS-R, CVLT, JLO, Letter and category fluence, TMT-A, TMT-B, WCST | PCA  X2, Bentler's CFI | 99 SCZ, 167 their unaffected siblings and 131 HC | EFA of the full sample yielded a 7-factor model that included verbal memory, working memory, visual memory, IQ/speed/fluency, executive function, attention and digit span. A CFA with maximum likelihood estimation revealed that the 7-factor model fit observed data from the three groups adequately. |
| Noh et al., 2010, [36] | CFA | II | Some subtests of WAIS, AVLT, CFT, DS-CPT, TMT-A/B, Letter and category fluence, One / Two-back test, Span:3 and 12 letters | Maximum likelihood Estimation extraction with Bollen-Stine bootstrapping | 114 stabilized SCZ and 120 HC | The multifactorial-6-factor model, which included the speed of processing, working memory, verbal learning and memory, visual learning and memory, attention/vigilance, and reasoning/problem solving as suggested by the MATRICS, showed the better goodness of fit than 1-2-3-factor, multifactorial 4-5 model and hierarchical 4-5-6 model. |
| Dickinson et al. 2011 [37] | A combination of EFA, CFA and MCFA | II | TAT, TBT, WCST, WAIS, Nback One/Two/three-back test, WMS, CVLT, WRAT | PCA  χ2 / df, Tucker Lewis Index, root mean square error of approximation | 496 SCZ, 504 unaffected siblings and 823 HC | EFA yielded factors for verbal memory, visual memory, processing speed, working memory span, n-back performance, and card sorting. CFA indicated that the hierarchical model, was a good fit for data from all groups. MCFA suggested that this hierarchical structure was fully invariant for controls and siblings. |
| Ojeda et al., 2012 [38] | CFA | II | Stroop, TMT, BTA-N, BTA-L, Digit Symbol Substitution Test/Logical Memory subtest/Digits Backward test/Letter Number Sequencing Task of WAIS-III, RCFT-IR, RCFT-DR, WCST | χ2, χ2/df, CFI, RMSEA, SRMR and NNFI | 100 SCZ and 53 HC | CFA supported the hypothesized 6-factor cognitive structure consisting of processing speed, attention, verbal memory, visual memory, working memory, and executive functioning (five-factor model, four--factor model and unifactorial model were tested). |
| Burton et al., 2013 [39] | CFA | II | MCCB | AIC, BIC, and Adjusted BIC | 183 SCZ or schizoaffective disorder | A three-factor MCCB model representing processing speed, attention/working memory, and learning fits the data well and was an improvement over a unifactorial model. Symbol coding, spatial span, and visual learning were the most robust predictors for each of the three factors; symbol coding proved to be the best single predictor of overall cognitive performance. |
| Lam et al.,2014 [40] | EFA  CFA | II | BACS, CPT, WASI, WCST, JLO | NFI, RFI, incremental fit index, NNFI/Tucker - Lewis index, CFI, RMSEA | 1012 English-speaking ethnic Chinese HC and 707 SCZ | A 3-factor model of executive functioning, vigilance/speed of processing and memory appeared the best to discriminate SCZ from HC (six-, four- and three-factor CFA models were fitted) |
| McCleery et al., 2015 [41] | CFA | II | Beta version of MCCB | AIC, AICc, BIC, SABIC, RMSEA, CFI, χ2, χ2/df | 281 SCZ | The seven correlated factors model (including speed of processing, attention/vigilance, working memory, verbal learning, visual learning, reasoning and problem solving, and social cognition) was the best fit compared to a single-factor model, a three correlated factors model including speed of processing, working memory, and general cognition, and a hierarchical model. |
| Lo et al., 2016 [42] | CFA | II | MCCB | SRMR, CFI, NNFI, χ2, RMSEA | 3 studies with 300 outpatients who have severe mental illness (54.3% schizophrenia-spectrum diagnoses) | A three factor structure of the MCCB-including processing speed, attention/working memory, and learning was replicated and demonstrated stronger model fit than the unifactorial structure. |
| **Factor analyses published after the MATRICS Consensus Initiative (Social cognition)** | | | | | | |
| Mancuso et al., 2011 [43] | EFA | III | FEIT, MSCEIT, PONS, AIHQ, TASIT- Part 3 | Maximum Likelihood extraction method | 85 SCZ, schizoaffective disorder or psychosis | EFA revealed 3 factors with relatively low inter-correlations that explained a total of 54% of the variance: Hostile attributional style, Lower-level social cue detection, and Higher-level inferential and regulatory processes. |
| Mehta et al., 2013, Schizophr Res. [44] | Systematic Review | I | SC and NC measures | N/A | The review comprised 20 studies with sample of SCZ or schizoaffective disorder | There was consistent evidence for the existence of distinct SC and NC factors. The results were inconsistent regarding factor structure of SC. Unitary, binary and multi-factorial constructs were reported, possibly due to variability and lack of comprehensiveness of the SC measures used. |
| Corbera et al., 2013 [45] | EFA | III | SAT-MC, BLERT, HINT, BORRTI, IRI | PCA | 30 SCZ or schizoaffective disorder and 24 HC | Social cognitive measures in subjects with SCZ and HC revealed three factors: Interpersonal Discomfort, Basic Social Cognition and Empathy. |
| Buck et al., 2016 [46] | EFA | III | AIHQ, FEIT, FEDT, beads in the jar task, HINT, TASIT | PACE | 65 SCZ or schizoaffective disorder and 50 HC | The factor structure of social cognition in schizophrenia separates hostile attributional style and social cognition skill. |
| Browne et al., 2016 [47] | CFA | II | BLERT, ER-40, Eyes, TASIT, HINT | FIML | 179 SCZ or schizoaffective disorder and 104 HC | Results of CFA support a one-factor model of social cognition for both individuals with SCZ and HC. |
| Mike et al., 2019 [48] | EFA | II | HINT, TASIT- Part 3, Penn Emotion Recognition Task, Penn Emotion Acuity Task, MSCEIT | Maximum likelihood method | 126 early course of SCZ or schizoaffective disorder | The results of this study indicate a three-factor structure of social cognitive skills within the early course of schizophrenia: Emotion Perception, Emotion Management, and ToM. |

**AGFI**: Adjusted Goodness-of-Fit index; **AIC**: Akaike information criterion; **AIC_c_**: corrected AIC; **AIHQ**: Ambiguous Intentions Hostility Questionnaire; **AVLT**: Auditory Verbal Learning Test; **BACS**: Brief Assessment of Cognition in Schizophrenia; **BD**: Wechsler Adult Intelligence Scale-Revised, Block Design subtest; **BDAE**: Animal Naming; **BIC**: Bayesian Information Criterion; **BLERT**: Bell Lysaker Emotion Recognition Task; **BMT**: Backward Masking Test; **BNFI**: Bentler and Bonnet’s non-normed fit index; **BORRTI**: Bell Object Relations Reality Testing Inventory; **BTA-N**: score on form N—numbers from Brief Test of Attention; **BTA-L**: score on form L - letters from Brief Test of Attention; **CFA**: confirmatory factor analysis; **CFI**: comparative fit index; **CFT**: Complex Figure Test; **COWAT**: Controlled Oral Word Association task; **CPT**: Continuous Performance Test; **CPT-IP**: Continuous Performance Test, Identical Pairs version; **CVLT**: California Verbal Learning Test; **DS-CPT**: Degraded Stimulus-Continuous Performance Test; **DSDT**: Digit Span Distractibility Test; **EFA**: exploratory factor analysis; **ER-40:** Penn Emotion Recognition Task; **Eyes** = Reading the Mind in the Eyes Test; **FAS**: form of Controlled Oral Word Association; **FE**: first-episode schizophrenia; **FEDT**: Face Emotion Discrimination Task; **FEIT**: Facial Emotion Identification Test; **FIML**: Full information maximum likelihood method; **GDS**: Gordon Diagnostics’ Continuous Performance Test, efficiency score; **GFI**: Goodness-of-Fit Index; **JLO**: Judgment of Line Orientation; **JOLO**: Judgment of Line Orientation; **HC**: healthy controls; **HINT**: Hinting Task; **HVLT**: Hopkins verbal learning test; **MAE**: Multilingual Aphasia Examination Visual Naming; **MAEVN**: Visual Naming subtest of the Multilingual Aphasia Examination; **MC**: Social Attribution Test-Multiple Choice; **MCCB**: MATRICS Consensus Cognitive Battery; **MCFA**: multiple groups CFA; **MSCEIT**: Mayer-Salovey-Caruso Emotional Intelligence Test; **NFI**: Normed Fit Index; **NNFI**: Non-Normed Fit Index; **PACE**: Conditional maximum likelihood extraction method; **PCA**: principal component analysis; **PCPT**: Penn Continuous Performance Test, efficiency score; **PONS**: The Half-Profile of Nonverbal Sensitivity; **PRFI**: relative fit index; **RBANS**: Repeatable Battery for the Assessment of Neuropsychological Status;**RCFT-IR**: immediate free recall from Rey Complex Figure Test; **RCFT-DR**: delayed recall from Rey Complex Figure Test; **RHY**: Seashore Rhythm Test; **RMR**: Root Mean Square Residual; **RMSEA**: root mean square error of approximation; **RMSR**: Mean Square Residual; **SABIC**: sample-size adjusted BIC; **SAT-** **IRI**: Interpersonal Reactivity Index; **SCFA**: single common factor analysis; **SCZ**: subjects with schizophrenia; **SRMR**: Standardized Root Mean Square Residual; **STEREO**: Stereognosis, total time; **Stroop-C**: color condition from Stroop Color Naming Test; **TAP**: Finger Tapping Test; **TASIT**: The Awareness of Social Inferences Test: Minimal Subscale; **TMT-A**: Trail Making Test-A; **TMT-B**: Trail Making Test-B; **VOC**: Wechsler Adult Intelligence Scale-Revised, Vocabulary subtest; **WAIS:** Wechsler Adult Intelligence Scale; **WASI**: Wechsler Abbreviated Scale of Intelligence; **WCST**: Wisconsin Card Sorting Test; **WCST-M**: Wisconsin Card Sorting Test-Modified; **WISC**: Wechsler Intelligence Scale for Children; **WMS**: Wechsler Memory Scale; **WMS-R**: Wechsler Memory Scale-Revised; **WRAT**: Wide Range Achievement Test.

**Table e3.** Effects of neurocognition and social cognition on functioning: characteristics and results of systematic reviews and meta-analyses.

| **Study** | **Type of study** | **Level of**  **evidence** | **Instruments: Predicitor measures/Outcome measures** | **Sample Size** | **Results** |
| --- | --- | --- | --- | --- | --- |
| Green et al 2000 [49] | Review  4 Meta-analyses | I | Neurocognitive batteries including measures of verbal ability, verbal and visual memory, executive functioning, visual spatial measures, attention/vigilance, information processing.  Types of functional outcome:  (1) Success in psychosocial skill acquisition, (2) laboratory assessments of instrumental skills and social problem-solving ability and (3) community outcome/daily activities. | 37 studies were included,  sample sizes ranging from 188 – 1002 SCZ or SA | Neurocognitive constructs (secondary verbal memory, immediate memory, vigilance, and executive functioning (card sorting) are related to functional outcome measures. The estimated pooled r's for the relationships ranges from 0.20 to 0.40, and the effect sizes  range from small-medium to medium-large. The relationships are larger when global or composite measures of neurocognition was considered.  The idea of "learning potential" requires a fundamental shift in assessment: from what the individual currently knows to what the individual is capable of learning. A concept like learning potential can be viewed as a mediator. |
| Green et al 2004 [50] | Review  Longitudinal studies, 6-month Follow up | I | Cognitive performance measures: Verbal IQ, REY-O, WMS-R logical memory and paired associates; WCST, VF, CPT, DS, HVLT proverbs, ER, TMT A and B, Aphasia screening, CERAD, Halstead categories, TAP, Stroop, LNS, RBANS, FF, Grooved pegboard, visual learning, CVLT, PASAT, RAVLT, BVRT, ToH.  Outcome measures: specific measures of work outcome (such as job performance and job tenure) to general measures of social adaptation and degree of independent living. SFS, QoL, SPS, Work Behavior Inventory, ADLs, Total number of hours worked. Community outcome (work, school, independent living): Quality of Well Being, Strauss Carpenter Outcome, SR, daily life activities Community adaptation, SAS II, Social Behavior Scale, GAF, ADLs, work. | 18 studies were included  1052 SCZ  (FEP and chronic) or schizoaffective disorder | Cognitive performance at one point in time predicts community functioning at a later point. 12 positive studies had effect sizes for the relationships in the medium to large range.  This review does not allow any firm conclusions about which cognitive constructs (e.g., attention, verbal memory, executive functions, etc.) are especially related to community outcome more than others. |
| Sergi et al. 2006 [51] | Research study  Structural equation modeling | III | Early visual processing (computerized visual masking procedures) score: FMT,  BMT.  RFS score: Independent living, social functioning, work functioning | 75 SCZ outpatients | Social perception both was predicted by early visual processing (standardized coefficient=0.57, p<0.05) and was predictive of functional status (standardized coefficient=0.44, p<0.05).  Social perception mediated the relationship between the predictor and outcome measures, as indicated by a significant indirect path between early visual processing and functional status (standardized coefficient for indirect effect=0.25, p<0.05). |
| Bowie et al. 2008 [52] | Research study  Longitudinal study | III | UPSA, SSPA, SLOF  DS Forward, DS Backward, DSDT,  LNS, RAVLT Trials I–V, RAVLT Long Delay Recall, RAVLT Recognition Hits  TMT-A, DSym, Category Fluency,  Phonological Fluency, WCST, TMT-B | 222 older SCZ outpatients | 4 cognitive factors emerged from the factor analysis: Attention/WM Verbal Learning and Memory Processing Speed Executive Functions.  The attention/working memory and processing speed factors predicted both social and living skills competence, while verbal memory and executive functions predicted living skills, but not social competence.  Results of the confirmatory path analyses: Modeling different behaviors (work skills, interpersonal, community activities):  Interpersonal Behavior was directly predicted by social, but not living skills, severity of depressive symptoms, processing speed, and executive functions.  Community Activity Participation was directly predicted by both social and living skills competence and the severity of positive symptoms. In this model, the processing speed factor predicted community performance directly and indirectly through its effects on both social competence and living skills.  Work Skills) are directly predicted by severity of depressive and positive  symptoms as well as both social competence and living skills. As in the other two models, processing speed made both a direct contribution to work skills as well as indirect effects through social competence and living skills |
| Ventura et al 2009 [53] | Meta-analysis  Cross-sectional study | I | Symptom assessment scales: BPRS, SANS, SAPS,  PANSS.  Neurocognitive tests: Logical Memory WMS-R, Paired Associates WMS-R, CVLT, HVLT, RVLT,  Buschke-List Learning Test, Rey-O, WMS-R  BVMT, Hooper Visual Orientation Test, DS Forward (WAIS), DS Backwards (WAIS), Spatial Span WMS-R, Letter-Number Sequencing (WAIS-III), DSTD, WCST, WAIS-BD, Gorham's Proverbs, Ravens Progressive Matrices, TMT A and B, SCWT, FAB, Canceling Test of Zazzo, COWAT, Chicago Word Fluency, Jones-Gorman Design Fluency Test, DS –WAIS, Lexical Decision Task, Hayling Sentence Completion Test, Purdue Pegboard, CPT, SOA, DSDT.  Functional outcome: community functioning (work or school performance, social functioning, independent living, and quality of life), skills assessment (role-play test such as AIPSS and UPSA | 73 studies were included  6519 SCZ | Negative symptoms were found to be significantly related to neurocognitive  functioning (p < .01) and were significantly related to functional outcome (r=−.42, p<.01) defined as community functioning and with skills assessment (r = −.28, p<.01). The relationship between neurocognition and community functioning and skills assessment was partially mediated by negative symptoms (Sobel test for indirect effects: z= 133.20, p<. 01 and z = 4.33, p<.01, respectively).  Negative symptoms mediated the relationship between neurocognition and functional outcome, appear to explain 17.6.% of variance in outcome.  Neurocognition and negative symptoms are both predictors of functional  outcome, negative symptoms might at least partially mediate the relationship between neurocognition and outcome.  The study sample was not randomly selected, the measurement of neurocognition was heterogeneous. |
| Addington et al. 2010 [54] | Research study Structural equation modeling | III | FEIT, FEDT, SCRT, SFRT, QLS, SFS,  AIPPS, COWAT, category instances, logical memory subtests of the WMS-R, RAVLT, Rey-O, LNS, WCT, DS-CPT, SPAN, TMT A and B, Grooved Pegboard, and the Stroop. | 43 FE, 53 SCZ multiple episodes,  55 HC | Illness-related effects on social cognition are fully mediated  by cognitive abilities. The link between cognition and social function is fully mediated by social cognition. A significant portion of the relation between group status (schizophrenia spectrum v. nonpsychiatric) and social function was mediated via cognitive and social-cognitive skills (R2 = 0.23, or 29.2% of 79.7%) and a substantial portion of illness-related associations with social function remains to be determined (γ = 0.56, or 70.8% of the total effect). |
| Fett et al 2011 [55] | Systematic review and meta-analysis | I | MCCB: the 7 cognitive factors identified by the MATRICS committee (1) reasoning & problem solving; (2) processing speed; (3) attention & vigilance; (4) working memory; (5) verbal learning & memory; (6) visual learning & memory; (7) verbal comprehension additional verbal fluency and a neurocognitive composite score were used.  Social cognitive domains: (1) ToM, (2) EP, (3) Social perception & knowledge  Domains of functional outcome: Community functioning: independent living skills and social or work functioning as indicators of everyday functioning.  Social behavior in the milieu mostly refers to observed behavior and comprises staff-ratings of the participants’ behavior in different treatment or (in)patient settings.  Social problem solving refers to the ability to recognize every- day social problems and to generate respective solutions. The outcome is based on observed behavior.  Social skills consist of behavior-based tests that assess interactional skills (e.g., eye contact, voice volume) in role-play tasks. | 52 studies were included  2692 patients with non-affective psychosis | The meta-analyses of correlations between cognitive domains and outcome domains revealed a stable pattern of significant small to large mean correlations between both cognitive domains and functional outcome (all p’s < 0.001–0.016).  Neurocognition and outcome:  The largest effect size was present for the association between verbal fluency and community functioning. Social behavior in the milieu had the strongest associations with verbal learning & memory and visual learning & memory.  Social problem solving had the strongest relationship with reasoning & problem solving. Social skills were also strongly associated with reasoning & problem solving but showed the strongest association with attention & vigilance.  Social cognition and outcome: The largest mean correlation was present for the relationship between ToM and community functioning.  Differential correlations between social- and neurocognition and community functioning: ToM was significantly stronger associated with community functioning than all NC domains (all p’s < 0.05), except verbal fluency. EP was more strongly associated with community functioning than attention & vigilance (p < 0.05). |
| Nüechterlein et al. 2011 [56] | Research study | III | CPT, visual BMT, SPAN, TMT A and B, DSDT, CVLT, SAS (interview format) | 47 SCZ | 3 cognitive factors (Working memory, attention and Early Perceptual Processing and Verbal memory and Processing speed) at baseline predicted 52% of the variance in work outcome (R = .719), with working memory (t = 4.47, P < .0001) and verbal memory and processing speed (t = 3.11, P = .004) making significant contributions. The level of these cognitive deficits at a stabilized outpatient baseline point was a very strong indicator of likelihood of return to work or school in this period. |
| Schmidt et al. 2011 [57] | Review  Structural equation modelling  Mediating analysis | II | Neurocognition:  COWAT, CPT, LNS, AVLT, WMS-R, PFA, ER – Questionnaire, SCST-R.  Function Outcome: GAF | 15 studies were included  148 SCZ | All neuro- and social cognitive measures had significant associations with social and/or psychological functioning, but none was significantly correlated with  vocational functioning. Neurocognition accounted for 14% of the variance in functional outcome  The direct path from neurocognition to functional outcome was no longer significant, as soon as the mediator was entered into the model (b = .15, P = .37). Instead, social cognition encompassing emotion perception and social schema was significantly associated with neurocognition (b = .67, P < .001) and was predictive of functional  status itself (b = .35, P < .05). The model explained 21% of the variance in functional outcome. All of the model fit indices were very good (v2 = 42.85, df = 32, P = .10,  CFI = .97, RMSEA = .04). The bootstrapping estimate revealed a significant indirect effect (b = .23, 95% CI = .037 to .85, P<.05). These data are consistent with a complete  mediation effect through social cognition.  Social cognition encompassing emotional perception and social schema was significantly associated with neurocognition, explained 21% of the variance in functional outcome.  Functional capacity mediate relationship between cognitive variables and global functional outcome. |
| Green et al. 2012 [58] | Research study | III | MSCEIT, TASIT, RAD  SANS, SAPS | 50 prodromal, 81 FE,  53 SCZ chronic | Emotion processing, ToM, and social relationship perception revealed clear impairment in schizophrenia across phase of illness. Importantly,  in this cross-sectional cohort study, any evidence of progression or improvement over the 3 phases of illness was seen.  social cognitive impairment starts early in the course of illness and remains stable. |
| Horan et al. 2012 [59] | Research study  12-month follow up | III | BPRS, MSCEIT, TASIT, RAD, RFS | 55 FE | Social cognition at baseline and follow-up  assessments predicted functioning at the 12-month follow-up across the domains of work, independent living, and social networks. Lower levels of baseline social cognition led to poorer  work outcome over 1 year. |
| Irani et al. 2012 [60] | Meta -  analysis | I | EP - Tasks (EP): emotion identification and emotion differentiation  Functional Outcome domains: community functioning, social behavior in the milieu, social problem solving, and social skills and functional outcome scales associated with EP: appropriate affect, communication dysfunction, global outcome, inappropriate behavior, independent living skills, interpersonal anguish, nonverbal  social skills, verbal social skills, occupational dysfunction, overall social skills, performance-based skills, relationships, self-care, social adjustment, social functioning and  work productivity. | 25 studies were included  1306 SCZ or schizoaffective disorder | Overall analysis of the relationship between EP and functional outcomes revealed an effect size of 0.31 (95% CI 0.13<δ<0.49, p = 0.001). the association between emotion identification and functional outcomes was statistically significant (N = 24, δ = 0.36, 95% CI 0.14<δ<0.57, p = 0.001).  When examining the relationship between EP as measured by emotion identification tasks and functional outcome domains, community functioning (Z = 2.17, p = 0.03), social problem solving (Z = 3.17, p = 0.002) and observed social skills (Z = 3.69, p < 0.001) were positively associated with emotion identification abilities while social behavior in the milieu was not (Z = −0.18, p = 0.86). Examination of the subdomains that comprise the functional domains revealed statistically significant effect sizes for independent living skills (Z = 2.95, p = 0.003), nonverbal communication (Z = 2.12, p = 0.03) and social skills/functioning (Z = 3.61, p < 0.001). |
| Ho et al. 2013 [61] | Research study | III | Observer Ratings of SLOF. Self-reported Ratings of Real-World Functioning:  Work and Social Impairment subscales of the BADS, RBANS, UPSA, PANSS, | 138 SCZ outpatients | Functional capacity significantly mediated the relations between cognitive ability and observer rated real world functioning, but not self-reported real-world functioning, with small to medium effect sizes observed for all outcomes.  The bootstrapping 95% confidence interval [.018, .206] revealed that the UPSA significantly mediated the relationship between the RBANS (cognitive ability) and SLOF Activities (observer rated participation in community activities).  The bootstrapping 95% confidence interval [.001, .110]  revealed that the UPSA significantly mediated the relationship between the overall cognitive ability (RBANS) and observer reported real-world functioning in work (SLOF Work Skills), |
| Galderisi 2014 [62] | Research study | II | Neurocognitive functions: MCCB  Social cognition: MSCEIT, FEIT, TASIT  Functional capacity: UPSA-B, SLOF | 921 SCZ | Illness-related variables (neurocognition, disorganization, avolition and positive symptoms) and incentives predict real-life functioning either directly or through the mediation of resilience, stigma, social cognition, functional capacity, and engagement with mental health services. The final SEM model explained about 54% of the SLOF variance.  Neurocognition exhibited the strongest, though indirect, association with real-life functioning, complex associations among investigated predictors, mediators, and real-life functioning. |
| Lepage 2014 [63] | Review | III | Logical memory and Verbal Pairs subtests of the WMS, RAVLT, Rey-O, Visual Reproduction subtests of WMS, BVMT, Elithorn`s maze test, picture completion and WAIS-BD, LNS, DS subtest of the WAIS, Spatial Span subtest of WMS, WM subtest of the TAP, TMT-A, mental tracking of the WMS, TMT-B, WCST, SCWT, Shift task of the CANTAB, CPT, d2 Test of Attention, DS of the WAIS, go-no-go task of the TAP, Cartoon Prediction subtest of the Four Factor Social Intelligence test, Expression Grouping, Social Translation subtests.  Independent living, social and interpersonal functioning, community activities and leisure, vocational functioning | 13 studies were included  1445 SCZ remitted, 1051 non-remitted, 62 FE remitted, 78 non-remitted | Verbal memory performance consistently exhibits a strong association with clinical outcome (poor outcome group) showing the largest deficits. A strong link between neurocognitive deficits and impairments in several aspects of functioning were found.  Need to standardize definitions of functional outcomes and distinguishing between one`s competence to perform tasks and what one does in everyday life. |
| Moore et al.  2015 [64] | Research study  2 independent samples | III | RBANS, UPSA-B, PANSS | Study 1: 435 SCZ, 390 BD Study 2: 205 SCZ | Study 1: The correlation between NCS and functional capacity was higher in the schizophrenia group, yet significant in both groups (schizophrenia group: r=0.65, p<0.001; bipolar group r=0.39, p<0.001). In both the schizophrenia and bipolar participants, when cognitive composite z-scores were >0 (indicating normal to above normal performance), cognition was not related to functional capacity. Study 2: the RBANS and UPSA-B were highly correlated in this study (r=0.68, p<0.001). When neuropsychological screening battery z-scores were >−1 (indicating low average to average performance), cognition was not related to functional capacity. |
| Strassnig et al. 2015 [65] | Analyses of pooled data from four separate studies  CFA, generic prediction model | II | MCCB, BACS, TMT-A, Animal naming fluency, LNS, HVLT, WAIS -3digit symbol task, UPSA-B  SLOF Scale: interpersonal functioning, everyday activities, vocational performance, PANSS | 4 study cohorts were included  821 SCZ or affective disorder | The overall 4-sample model was found to be associated with tenable fit: χ2 = 318.3 df = 180, RMSEA = .061, CFI = .944. The variance accounted for in interpersonal functioning was 23%, while the variance accounted for in everyday activities was 28%, and variance accounted for in work functioning was 19%.  Cognition and functional capacity were predictors of the severity of deficits in performing everyday activities and vocational outcomes but would not predict social functioning.  Negative symptoms that measure social motivation and engagement were related to social outcome. Further, no negative symptoms manifested any significant correlations with either everyday activities or vocational outcomes. |
| Bechi et al. 2017 [66] | Research study | III | PANSS, WAIS-R, BACS, RMET, FEIT, IRI, PAS, Risky Families Questionnaire (RFQ), QLS, UPSA-B | 79 SCZ | The forward stepwise regression analyzing predictors of functional capacity, evaluated with UPSA-B, yielded a significant model (F (4,73) =10.26, p < 0.0001, Adjusted R2=0.32), that included IQ, IRI, PAS and BACS, explaining 32% of the variance of functional outcome with a significant effect only for IQ (p < .0001 β=0.46). A The forward stepwise regression analyzing predictors of functional performance, evaluated with QLS, yielded a significant model (F (5,72) =6.99, p < 0.0002, Adjusted R2=0.28), that included IRI, FEIT, PANSS, RFQ and RMET, explaining 28% of the variance in daily functioning, with significant effects for IRI (p=0.007 β=0.34),) FEIT (p=0.020 β=0.30) and PANSS total score (p=0.003 β=−0.28). |
| Galderisi et al. 2018 [67] | Multicenter, cross-sectional study | II | Neurocognitive functions: MCCB  Social cognition: MSCEIT, FEIT, TASIT  Functional capacity: UPSA-B, SLOF | 921 SCZ | Network Analysis: The network analysis showed that functional capacity and everyday life skills were the most central and highly interconnected nodes in the network. Functional capacity bridged cognition with everyday life skills. Working memory node showed the highest centrality: it was strongly connected to all the other neurocognition nodes and bridged them with social cognition and functional capacity and, through functional capacity, with real-life functioning nodes. Verbal learning, visuospatial learning, and attention were also directly connected with functional capacity. The TASIT-1 node was connected to all the other social cognition nodes and bridged the social cognition domain with the functional capacity node and, through the functional capacity node, with the real-life functioning nodes.  In the real-life functioning domain, everyday life skills had the highest centrality. It was strongly connected with the other 2 functioning nodes (interpersonal relationships and work skills) and with functional capacity.  The functional capacity and Specific Level of Functioning Scale (SLOF) everyday life skills were the most central nodes of the whole network (hAs Functional capacity bridged neurocognition and social cognition with the everyday life skills node. |
| Strassnig et al. 2018 [68] | Research study | III | MCBB, PANSS, SLOF, PANSS reduced expression, PANSS reduced emotional experience | 821 SCZ chronic | Cognitive test performance was correlated with interpersonal relationships, everyday activities, and work skills in the overall sample. The shared variance for these correlations was 1%, 12%, and 26% respectively across the three functional domains.  In patients with neuropsychologically impaired t scores ≤40, both everyday activities and work were correlated with cognitive test performance with shared variance at 5% and 3% respectively. |
| Bosia et al.2019 [69] | Research study | III | BACS, WCST, CPT, WAIS-R  QLS, PANSS | 214 SCZ | Processing speed (Symbol Coding performance) turned out to be the first-ranked predictor of functional outcome and attention measures and verbal memory were shown to have a major impact on functioning. Four Profiles were constructed based on specific levels of functioning, each characterized by a distinct distribution of key clinical and neurocognitive measures. |
| Halverson et al. 2019 [70] | Meta-analysis | I | Neurocognition domains based on NC factors identified by the MATRICS Committee: attention and vigilance, processing speed, reasoning and problem solving, verbal comprehension, verbal fluency, verbal learning and memory, visual learning and memory, working  memory, and combined neurocognition  Social cognitive domains: SC domains were based  on SC factors identified by the MATRICS Committee: attribution bias, emotion perception and processing, social knowledge and perception, theory of mind, and combined social cognition  Domains of functional outcome: community functioning (e.g., activities of daily life and relationships), social behavior in the milieu (e.g., observed behaviors in  a specific context), social problem solving (e.g., abilities to address a social problem or generate solutions), and social skills (e.g., social interaction  abilities like eye contact and conversation skills | 116 studies were included  12.868  Schizophrenia Spectrum Disorder | A random-effects meta-analysis yielded a medium-sized average correlation between overall NC (i.e., across all NC domains) and functional outcomes and (ûp=0.21, 95% CI [0.18, 0.24], p < .01) based on 399 effect sizes; 60.88% of the variation in effect sizes was due to heterogeneity between studies (Q=1556.36, p < .001; I2=60.88).  Mean correlation estimates across specific NC functional outcome relationships were small to medium in size (ûp=.06–.33). The smallest effect was observed between verbal comprehension and social behavior in the milieu (ûp=.06, p=.60). The largest effect was observed between overall neurocognition and social skills (ûp=.33, p < .001).  An association between social cognition and neurocognition, and functional outcomes demonstrated significant small-to-medium effect sizes. Social cognition explained more unique variance in functioning than neurocognition (7.3% vs 4.4%, 9.2% total average variance.  A random-effects meta-analysis yielded an overall medium correlation between all domains of social cognition and functional outcomes, (ûp=0.24, 95% CI [0.19, 0.28], p < .01) based on 119 relationships with 62.29% of the variation in effect sizes due to heterogeneity between studies (Q=300.10, p < .001; I2=62.29). Random-effects meta-analyses examining relationships between summary SC and specific functional outcome domains also yielded medium correlations (ûps=.21–.46, ps < .01) based on 3 – 82 relationships.  Mean correlation estimates across specific SC-functional outcome relationships were small to medium in size (ûp=.08–.38). The smallest effect was observed between attribution bias and community functioning (ûp=.08, p=.16). The largest effect was observed between theory of mind and social skills (ûp=.38, p < .001).  A random effects mediation analysis explored the potential role of SC as a mediator between NC and functional outcomes in the same subset of studies. Correlations between NC and SC allowed for modeling of dependency between these two domains. Results suggest SC is a partial mediator between NC and functional outcomes. When SC was included as a mediator in the model, the relationship between NC and functional outcome decreased but was still significant (ûp=.14, p < .01). |
| Ojeda et al. 2019 [71] | Structural equation modeling (SEM) approach | II | DAS-WHO Scale; QLS | 165 SCZ | Neurocognitive capacity linked to clinical symptoms and premorbid functioning showed good fit to the observed data (Satorra-Bentler χ2 = 604.83; RMSEA = .08; SRMR = .11; NNFI = .96; CFI = .97).  Processing speed, verbal memory and premorbid functioning predicted outcome; verbal fluency predicted outcome via negative symptoms. |
| Amoretti et al 2020 [72] | Prospective study  Path analysis model  2-years follow-up | III | The neuropsychological battery:  (CPT-II; Verbal Learning Test Spain Complutense for adults (TAVEC), DS Subtest, LNS of the WAIS-III, WCST, GCI.  Cognitive reserve: IQ (WAIS-III), PAS Scale,  FAS areas: autonomy, occupational functioning, cognitive functioning, financial issues, interpersonal relationships, and leisure time. | 211 FE at baseline  139 FE at follow – up | At follow-up, the effect of attention (p = 0.003) and negative symptoms (p = 0.012) assessed at baseline on functioning was partially mediated by CR ( p = 0.032 and 0.016), whereas the relationship between verbal memory ( p = 0.057) and functioning was mediated by CR ( p = 0.014). Verbal memory and positive and total subscales of PANSS assessed at follow-up were partially mediated by CR and the effect of working memory on functioning was totally mediated by CR |
| Gonzalez-Ortega et al. 2020 [73] | Followed up study  Path analysis | III | Cognitive domains are assessed: processing  speed, attention, verbal memory, working memory executive function, and premorbid IQ.  Cognitive reserve: Estimated premorbid IQ, educational level, and occupational  attainment  Social cognition: MSCEIT,  Functional outcome: FAS Test | 282 FE | At 2 years of follow-up, social cognition acted as a mediator between cognitive reserve and functioning. Likewise, social cognition was a mediator between verbal memory and functional outcome. The results of the bootstrap analysis confirmed these significant mediations (95% bootstrapped CI (−10.215 to −0.337) and (−4.731 to −0.605) respectively). |
| Hochberger et al. [74] | Part of the Consortium of Genomics in schizophrenia  (COGS-2) study | I | SLOF, UPSA, Penn-CNB. | 1290 individuals, 684 SCZ,  606 HC | The magnitude of deviation from expected cognitive ability was associated with neurophysiological abnormalities, longer illness duration, higher levels of negative symptoms and worse psychosocial functioning. Patients with significant deviation had  lower psychosocial functioning (SOF total score: F [1500] ¼ 14.54, p < 0.001, hp 2 ¼ 0.028, x Diff ¼ -2.24, 95% CIDiff ¼ [-3.40, -1.09]; UPSA total score: F [1500] ¼ 36.25, p < 0.001, hp 2 ¼ 0.068, x Diff ¼ -7.94, 95% CIDiff ¼ [-10.53,-5.35]).  Assessment of cognitive deviation between observed and expected cognitive ability is a core cognitive feature of schizophrenia. |
| Lewandowski et al. 2020 [75] | Research study | III | MCCB  MCAS including independence in daily living, social interest and involvement, work and leisure activities, and participation in treatment. | 211 patients with psychotic disorder (SCZ, schizoaffective, BD) and  87 HC | MCAS was correlated with all MCCB cognitive domains and the Composite.  The final regression model predicting community functioning was significant (F=36.78, p>.0001), and explained 47% of the variance in MCAS score (Adjusted R-squared=.47). Social cognition (but not processing speed) were significant. |
| Modinos et al. 2020 [76] | Research study EU-GEI | III | Degraded Facial Affect Recognition Task, GAF | 213 CHR | Within the CHR sample, better anger recognition at baseline was associated with worse functional outcome within 2 years of baseline (odds ratio [OR], 0.88; 95% CI, 0.78-0.99; P = .03). Poor functional outcome in individuals at CHR was found to be associated with baseline abnormalities in recognizing negative emotion. |
| Rojnic Kuzman et al. 2020 [77] | prospective cohort study, longitudinal naturalistic study | III | Neurocognitive tests: RAVLT-B, DS Backwards, FAB, TMT-B, Rey-O, Phonetic Fluency test, GAF. | 129 FE after 18-month of treatment | Functional outcome at month 18 assessed with GAF scores was predicted by sex, neurocognitive symptoms, and GAF at baseline, as well as with pharmacotherapy and daily hospital treatment. Female sex, higher initial scores at RAVLTB, and better GAF scores at baseline predicted higher GAF scores at month 18, indicating better functional outcome. Likewise, being treated within a psychosocial multimodal program as a part of day hospital and taking more than one antipsychotic at baseline predicted better final GAF scores, whereas taking benzodiazepines at baseline predicted lower final GAF scores. |
| Salagre et al 2020 [78] | Class growth analysis  2-years follow-up | III | MCCB  4 trajectories of psychosocial functioning | 261 FE | Latent class growth analysis identified four distinct trajectories: Mild impairment- Improving trajectory (Mi-I) (38.31% of the sample), Moderate Impairment-Stable trajectory (Mo-S) (18.39%), Severe Impairment-Improving trajectory (Se-I) (12.26%), and Severe Impairment-Stable trajectory (Se-S) (31.03%). Participants in the Mi-I trajectory were more likely to have higher parental socioeconomic status, less severe baseline depressive and negative symptoms, and better premorbid adjustment than individuals in the Se-S trajectory. Participants in the Se-I trajectory were more likely to have better baseline verbal learning and memory and better premorbid adjustment than those in the Se-S trajectory. Verbal learning and memory are associated with more resilient trajectories of psychosocial functioning. |
| Vita et al 2020 [79] | Real – world multicenter study of the Italian Network for Research on Psychoses | I | PAUSS, MCCB,  Functional capacity: UPSA-B  Real world functioning: SLOF | 921  Schizophrenia spectrum disorders  ASD symptoms in schizophrenic disorder  3 groups of subjects | Subjects with more severe ASD symptoms showed a poorer performance in the processing speed (p=0.010), attention (p=0.011), verbal memory (p=0.035), and social cognition (p=0.001) domains, and an overall lower global cognitive composite score (p=0.010). Subjects with more severe ASD symptoms also showed poorer functional capacity (p =0.004), real-world interpersonal relationships (p < 0.001), and participation in community-living activities (p<0.001). Schizophrenic patients with more severe ASD symptoms showed poorer processing speed, attention, verbal memory, social cognition, and lower global cognitive composite score.  Comparisons in cognitive and functional measures ASD symptoms may have an impact on the life of people with schizophrenia. |

**ADLs**: Alzheimer’s Disease Assessment Scale; **AIPSS:** Assessment of Interpersonal Problem Solving Skills; **ASD**: Autism Spectrum Disorders;  **AVLT**: Auditory Verbal Learning Test; **BACS**: Brief Assessment of Cognition in Schizophrenia**; BADS:** Behavioral Activation for Depression Scale; **BD**: Bipolar disorder; **BMT**: Backward Masking Test; **BPRS:** Brief Psychiatric Rating Scale; **BVMT:** Benton Visual Memory Test; **BVRT**: Benton Visual Retention Test; **CERAD**: The Consortium to Establish a Registry for Alzheimer’s Disease; **CI**: confidence interval; **CANTAB**: the Cambridge Neuropsychological Test Automated Battery; **GCI**: Global Cognitive Index; **CFA**: confirmatory factor analysis; **CFI**: comparative fit index; **CHR**: Clinical High Risk; **COWAT**: Controlled Oral Word Association task; **CPT**: Continuous Performance Test; **CVLT**: California Verbal Learning Test;; **DAS-WHO Scale**: WHO Disability Assessment Schedule; **DS:** Digit Span; **DS-CPT**: Degraded Stimulus-Continuous Performance Test; **DSDT**: Digit Span Distractibility Test; **DSym:** Digit Symbol Substitution Test; **EP**: Emotion Perception**; ER**: Emotion Recognition; **RMET**: Reading the Mind in the Eyes Test; **FAB:** Frontal Assessment Battery; **FAS Test:** Controlled Oral Word Association Test; **FAS**: Functioning Assessment short ; **FE**: first-episode schizophrenia; **FEDT**: Face Emotion Discrimination Task; **FEIT**: Facial Emotion Identification Test; **FF:** Figural Fluency; **FMT**: Forward Masking Test; **GAF**: Global Assessment of Functioning**; GCI:** Global Cognitive Inventory**; GVO:** good vocational outcome; **HC**: healthy controls; **HINT**: Hinting Task; **HVLT**: Hopkins verbal learning test; **IPSA-Q**: Internal, Personal and Situational Attributions Questionnaire; **IRI:** Interpersonal Reactivity Index; **ISMI:** Internalized Stigma of Mental Illness Scale; **IQ**: intelligence quotient;**; LNS**: Letter number span; **MAS**: Metacognition Assessment Scale;; **MCAS:** Multnomah Community Ability Scale; **MCCB**: MATRICS Consensus Cognitive Battery; **MMSE:** Mini Mental State Examination; **MSCEIT**: Mayer-Salovey-Caruso Emotional Intelligence Test; **NART**: The National Adult Reading Test; **NC**: Neurocognition; **PANSS**: Positive and Negative Syndrome Scale; **PAS**: Education and participation in leisure; **PASAT**: Paced Auditory Serial Addition Test; **PAUSS:** The PANSS Autism Severity Scale; **PENN – CNB:** University of Pennsylvania Computerized Neurocognitive Battery; **POV:** Poor Vocational outcome**; PSI**: Processing Speed Index; **QoL**: Quality of Life; **QLS**: Quality of Life Scale**; Q-LES-Q Index:** Quality of Life Enjoyment and Satisfaction Questionnaire; **QLS-BR:** Quality of Life Scale Brazilian Adaptation; **PCA**: principal component analysis; **RAD**, Relationship Across the Domains test; **RAVLT**: Rey auditory-verbal learning test; **REY-O:** Rey-Osterrieth Complex Figure Test; **RBANS**: Repeatable Battery for the Assessment of Neuropsychological Status; **RFS**: Role Functioning Scale; **RMETRMR**: Root Mean Square Residual; **RMSEA**: root mean square error of approximation; **RT:** Reaction Time; **SANS:** Scale for the Assessment of Negative Symptoms**; SAS:** Social Adjustment Scale; **SAPS**: Scale for the Assessment of Positive Symptoms; **SCoRS**: Schizophrenia Cognition Rating Scale; **SCRT:** Social Cue Recognition Test; **SCST-R:** computerized Social Component Sequencing Task-Revised **SCWT**: The Stroop-Color Word Test; **SCZ**: subjects with schizophrenia; **SFRT**: the Situational Features Recognition Test; **SFS**: Social Functioning Scale; **SLOF**: Specific Level of Functioning; **SOA:** Span of Apprehension; **SPAN:** Span of Apprehension; **SPS:** Social Problem Solving; **SR:** social relations; **SRMR**: Standardized Root Mesan Square Residual; **SSPA:** Social Skills Performance; **TAP**: Finger Tapping Test; **TASIT**: The Awareness of Social Inferences Test: Minimal Subscale; **TMT-A**: Trail Making Test-A; **TMT-B**: Trail Making Test-B; **ToH:** Tower of Hanoi; **ToM**: Theory of Mind; **UPSA**: UCSD Performance-Based Skill Assessment; **VF**: Verbal Fluency; **WAIS-R:** Wechsler Adult Intelligence Scale-Revised; **WAIS-R-BD:** Wechsler Adult Intelligence Scale-Revised, Block Design subtest**;** **WCST**: Wisconsin Card Sorting Test; **WM**: working memory; **WMS**: Wechsler Memory Scale; **WMS-R**: Wechsler Memory Scale-Revised

**Table e4.** Effects of neurocognition on QoL: study characteristics and results.

| **Study** | **Type of study** | **Level of evidence** | **Instruments** | **Sample size** | **Results** |
| --- | --- | --- | --- | --- | --- |
| Ritsner 2007 [80] | Research study | III | CANTAB Tests:  five cognitive domains: motor skills, attention,  memory, learning, sustained attention, and executive function: Big/Little Circle (BLC), Reaction Time (RTI), Matching to Sample Visual Search (MTS), Delayed Matching to Sample (DMS), Pattern Recognition Memory  (PRM), Spatial Recognition Memory (SRM), Spatial Span (SSP), Paired Associates Learning (PAL), Rapid Visual Information Processing (RVP), Spatial Working Memory (SWM), Intra/Extra Dimensional Set Shift (IED), and Stockings of Cambridge (SOC).  Q-LES-Q and QLS | 62 SCZ chronic | When cognitive variables were considered on their own, the cognitive functions  explained from 13 to 35% of the variability in self-report and observer rated HRQL domain scores.  Significant association of the cognitive deficits with QoL impairment. a deficit in executive functions (IED, SWM, SOC), memory (SSP, DMS), visual sustained attention  (RVP), learning (PAL), and motor skills (RTI, BLC) were significantly associated with quality-of-life impairment in various domains. Significant positive indicators of the total variance in the Q-LES-Index scores were performance in executive functions (IED, R2 = 20.3%, and SWM, R2 = 8.3%) and motor skills (RTI, R2 = 7.2%).  Performance in executive functions appears to significantly contribute to predicting the general quality of life or QLS, total score (IED, R2 = 8.5%), instrumental role functioning (IED, R2 = 12.3%, and SOC, R2 = 10.5%), and interpersonal relations and social network (IED, R2 = 15%).  Deficits in memory functions (SRM) accounted for 13.5% of the total variance in instrumental role functioning, and for 7.7% of the variability in intrapsychic foundations. In addition, sustained attention (RVP) and motor skills (RTI) markedly contributed to the prediction of the total variance in the instrumental role functioning ratings (14.1%, and 11.8%, respectively). |
| Lipkovich et al. 2009 [81] | Clinical trial | III | LNS of the WAIS-III, verbal memory. RAVLT, WAIS – R DS, COWAT, QLS scale subdomains of functioning: Instrumental, Intrapsychic, Interpersonal  PANSS | 414 SCZ | At baseline Processing speed showed effects (via negative symptoms) on three domains of QoL.  At 24 weeks, changes in processing speed affected changes in functioning, both directly and indirectly via changes in negative symptoms (PANSS Neg). |
| Tomida et al. 2010 [82] | Research study | III | WCST, CPT, Japanese Schizophrenia Quality of Life Scale (JSQLS): three areas: psychosocial conditions; motivation/energy and symptoms/side-effects. PANNS | 52 SCZ | Cognitive function affects subjective QoL (symptom side/effects area). Sustained attention in subjects would be lower than that in the normal population, and this did not affect subjective QOL. |
| Woon et al. 2010 [83] | Research study | III | BACS, WHOQOL-BREF, GAF | 83 SCZ outpatients | Cognitive dysfunction (working and verbal memory) and lower GAF were correlated with poorer QoL. GAF did not mediate the neurocognitive deficits on QOL. |
| Kurtz and Tolman 2011 [84] | Research study | III | Illness Insight: Lack of Judgement from the PANSS.  Vocabulary, digit span and digit symbol subtests of the WAIS-III and IV), FAS, CVLT, Penn Conditional Exclusion Test (PCET), SWL | 71 SCZ stable or schizoaffective disorder | Three measures of elementary neurocognition, crystallized verbal ability, attention, and executive-function, were all inversely related to SQOL. Verbal ability, attention, working memory and problem-solving were inversely related to subjective QoL.  Illness insight was inversely related to SQOL. The relationship between neurocognitive deficits and satisfaction with life was not mediated or moderated by a measure of insight into illness. |
| Maat et al. 2012 [85] | Part of the multicenter study: Genetic Risk and Outcome in Psychosis (GROUP) | II | WHOQOL-BREF, degraded facial affect recognition task, HINT, BVMT, WAIS III, PANSS | 1032 SCZ, 1011 siblings, 552 HC | In the regression analysis the variables age, gender, IQ, total PANSS score and social cognition explained 17% of the variability in QOL in patients, (F (7,805) = 24.16, pb.001).  The hinting task was a significant predictor of QOL in this model (ß=−.085, p=.02).  The interaction between total PANSS score and performance on the hinting task significantly predicted the QOL of patients. For the interaction factor the regression coefficient (ß) was −.358. By Cohen's conventions, a combined effect of this magnitude can be considered ‘large’ (f2 = −.256).  Theory of mind but not emotion perception or neurocognition was associated with QoL in patients. |
| Tolman and Kurtz 2012 [86] | Meta-analytic investigation | I | WAIS-Vocabulary Subtest, Letter Fluency, COWA-FAS, CPT,  DS Subtest of the WAIS,  DS, WMS-Logical Memory, Immediate (LMI) and Long Delay (LMII), CVLT/HVLT/RAVLT immediate and delayed, DSym, TMT A and B, WCST,  WHOQOL-BREF, QOL, SWL. | 20 studies were included  (10 objective and 10 subjective)  1615 SCZ | Small effect sizes were found for the relationship between [WAIS]-Vocabulary, d = 0.34, 95% CI: 0.13/0.55), working memory (WAIS-DS, d = 0.26, 95% CI: 0.11/0.41; WAIS-Letter–Number Sequencing, d = 0.17, 95% CI: 0.06/0.28), CVLT/HVLT/RAVLT immediate, d = 0.37, 95% CI: 0.24/0.51; CVLT/ HVLT/RAVLT delayed, d = 0.13, CI: 0.01/0.25), processing speed (WAIS-DS, d = 0.23, 95% CI: 0.10/0.36), and objective QOL. Executive function was found to have a small-medium effect size relationship to objective QOL (WCST-PE, d = 0.28, 95% CI: 0.14/0.41; WCST-CAT, d = 0.55, 95% CI: 0.38/0.72).  Most neurocognitive domains were not significantly correlated with subjective  QOL, except for crystallized verbal ability and processing speed, which were negatively correlated with subjective QOL and letter fluency, which was positively  correlated with subjective QOL. Small effect sizes were revealed for verbal IQ (WAIS-vocabulary, d = _0.29, 95% CI: _0.49/_0.10), processing speed (WAIS-DS d = _0.19, 95% CI: _0.36/_0.02), and letter fluency (d = 0.26, 95% CI: 0.09/0.43). |
| Cruz et al. 2016 [87] | Research study  Interview-based assessment of cognition | III | SCoRS: memory, reasoning and problem solving, working memory, language production, and motor  skills. (interview based, patient, informant), QLS-BR, PANSS -P, PANSS-N | 79 SCZ outpatients | Cognitive deficits assessed by SCoRS, and negative symptoms assessed by PANSS are predictors of quality of life in schizophrenia. A multivariate regression including  PANSS-N and SCoRS had an adjusted R-squared of 0.263 and both coefficients were significant at a = 1%.  The results indicate that interview-based instruments are also suitable to detect the impact of cognitive impairment on quality of life. |
| Shin et al. 2016 [88] | Research study | III | SSTICS: domains of memory, attention, executive function, lan- guage, and praxis, ISMI, Schizophrenia Quality of Life Scale Revision 4 (SQLS-R4). | 70 SCZ outpatients | Self-perceived cognitive deficits are associated with QoL  Multiple regression analysis showed that the SSTICS and ISMI scores significantly predicted the SQLS-R4 score (P,0.01). Mediation analysis revealed that the strength of the association between the SSTICS and SQLS-R4 scores decreased from β=0.74 (P,0.01) to β=0.56 (P,0.01), when the ISMI score was statistically controlled. The Sobel test revealed that this difference was significant (P,0.01), indicating that internalized stigma partially mediated the relationship between self-perceived cognitive deficits and quality of life. |
| Takahashi et al. 2017 [89] | Comparison of high-risk individuals with patients with schizophrenia and healthy controls | III | BACS, SCoRS, QLS, SOFAS | 33 CHR, 45 SCZ,  63 HC | Regression analyses demonstrated that the QLS score was predicted by SOFAS (for both groups) and SCoRS (for CHR-P) scores.  The QLS score was significantly predicted by SOFAS (Beta = 0.552, t = 4.35, p < 0.001) and SCoRS global rating score (Beta = −0.409, t = −3.22, p = 0.004) for the CHR-P group (Adjusted R2 = 0.727), and by SOFAS (Beta = 0.713, t = 6.67, p < 0.001) for the schizophrenia group (Adjusted R2 = 0.497).  No significant effects of cognition on QoL in schizophrenia |
| Lu et al. 2018 [90] | Meta-analysis | I | QOL: WHOQOL, SF-36 | 18 studies were included  2425 SCZ  2072 HC | Compared with HC, SCZ had significantly poorer overall QOL [11 studies; SMD:-1.07 (95%CI:-1.44, -0.70), P < 0.001] as well as in the physical, psychological, social and environmental QOL domains. |
| Kurtz et al. 2019 [91] | Research study | III | MCCB, CPT-IP and MSCEIT  Psychosocial functioning assessment: DAS-WHO2  Subjective quality of Life assessment: SWL, PANSS | 59 Patients SCZ or schizoaffective disorder within 5-years of diagnosis | With respect to cognition, working memory scores from the MATRICS battery were linked to SWL Work subscale scores (r=−0.30) and total scores (r=−0.27). With respect to cognition, no measures from the MATRICS battery were linked to WHODAS ratings.  Severity of positive and negative symptoms were linked to total WHODAS scores. With respect to the Life Activities subscale from the WHODAS only higher positive symptoms were linked to poorer scores on the Life Activities subtest. Both higher ratings of positive and negative symptoms from the PANSS were linked to poorer Community Participation scores on the WHODAS. |
| Ehrminger et al 2020 [92] | Multivariate model  Structural equation modeling | I | Cognition: Memory: CVLT, Doors test, WAIS DS and Arithmetic, CPT, WAIS Matrices and Similarities, Category VF (animal), Six Elements test (error score), TMT A and B, WAIS Code,  Cognitive reserve: number of school years since the first  Mandatory grade, NART, Information Subtest of the WAIS.  Insight: BIS, SUMD.  Functioning: GAF, QOL: 8 S-QoL sub-scores: psychological well-being, self-esteem, family relationships, relationships with friends, resilience, physical well-being, autonomy, and sentimental life | 776 SCZ or schizoaffective disorder  FondaMental Foundation FACE-SZ cohort within a French national network | Cognitive reserve is associated with QoL only through its relationship with cognitive performance, and not via functioning (the total effect of the multiple mediation is β = −0.1, p = 0.01).  Cognitive performance is negatively associated with QoL, and this association is not mediated by insight, notably because cognition was not directly linked to insight.  Cognition and functioning were only indirectly associated via symptoms. |
| Hoertel et al. 2021 [93] | Multicenter study  Structural equation modeling | III | MMSE, QoL Scale (QLS) 4 domains: interpersonal relations, intrapsychic foundations, instrumental role and common objects and activities.  GAF | 353 older SCZ multicenter sample French community mental health teams | Severity of general psychopathology symptoms, depression, and cognitive impairment,  reduced overall functioning, low education, greater number of medical conditions and greater number of antipsychotics were independently and negatively associated with quality of life. Negative symptoms had an additional negative effect above and beyond the effects of other factors. R2 of the model was 0.52, indicating that the model explained 52% of the outcome variance.  General psychopathology symptoms, depression and cognitive impairment were independently related to lower quality of life in older adults with schizophrenia. |

**BACS**: Brief Assessment of Cognition in Schizophrenia; **BIS**: Birchwood Insight Scale; **BVMT:** Benton Visual Memory Test; **CHR**: Clinical High Risk; **CI**: confidence interval; **CANTAB**: the Cambridge Neuropsychological Test Automated Battery; **COWAT**: Controlled Oral Word Association task; **CPT**: Continuous Performance Test; **CVLT**: California Verbal Learning Test; **DAS-WHO**: WHO Disability Assessment Schedule **DS:** Digit Span; **DSym**: Digit Symbol Substitution Test; **FAS**: Functioning Assessment short; **GAF**: Global Assessment of Functioning; **HC**: healthy controls; **HINT**: Hinting Task; **HVLT**: Hopkins verbal learning test; **ISMI:** Internalized Stigma of Mental Illness Scale; **IQ**: intelligence quotient;**; LNS**: Letter number span; **LES-Q**: Life Enjoyment and Satisfaction Questionnaire; **MCAS:** Multnomah Community Ability Scale; **MCCB**: MATRICS Consensus Cognitive Battery; **MMSE:** Mini Mental State Examination; **MSCEIT**: Mayer-Salovey-Caruso Emotional Intelligence Test; **NART**: The National Adult Reading Test; **PANSS**: Positive and Negative Syndrome Scale; **QoL**: Quality of Life; **QLS**: Quality of Life Scale; **Q-LES-Q Index:** Quality of Life Enjoyment and Satisfaction Questionnaire; **QLS-BR**: Quality of Life Scale Brazilian Adaptation; **RAVLT**: Rey auditory-verbal learning test; **SCoRS**: Schizophrenia Cognition Rating Scale; **SCZ**: subjects with schizophrenia; **SMD**: Standardised mean difference; **SOFAS**: Social and Occupational Functioning Assessment Scale; **SPAN:** Span of Apprehension; **SQLS-R4**: Schizophrenia Quality of Life Scale Revision 4; **SSTICS:** Scale to Investigate Cognition in Schizophrenia; **SUMD**: Test of Awareness of mental Disorder; **SWL:** Satisfaction with Life Scale; **SSTICS**: Subjective Scale To Investigate Cognition in Schizophrenia; **TMT-A**: Trail Making Test-A; **TMT-B**: Trail Making Test-B; **VF**: Verbal Fluency; **WAIS-R:** Wechsler Adult Intelligence Scale-Revised; **WHOQOL-BREF**: World Health Organization Quality-of-Life Scale; **WMS**: Wechsler Memory Scale; **WMS-R**: Wechsler Memory Scale-Revised

**Table e5.** Effects of social cognition on QoL: study characteristics and results.

| **Study** | **Type of study** | **Level of evidence** | **Instruments** | **Sample Size** | **Results** |
| --- | --- | --- | --- | --- | --- |
| Kurtz et al. 2012 [94] | Intervention study  Study on the effects and predictors of change associated with a rehabilitation programme | III | PSI from the WAIS III and IV, PCPT, CVLT-II, PCET, PEAT,  PANSS, QOL, SWL | 44 SCZ outpatients | Changes in objective QoL and changes in subjective SWL both correlated with the Penn Emotion Acuity Test. |
| Maat et al. 2012 [85] | Part of the multicenter study: Genetic Risk and Outcome in Psychosis (GROUP) | II | WHOQOL-BREF, degraded facial affect recognition task, HINT, BVMT, WAIS III, PANSS | 1032 SCZ, 1011 siblings, 552 HC | In the regression analysis the variables age, gender, IQ, total PANSS score and social cognition explained 17% of the variability in QOL in patients, (F (7,805) = 24.16, pb.001). The hinting task was a significant predictor of QOL in this model (ß=−.085, p=.02).  The interaction between total PANSS score and performance on the hinting task significantly predicted the QOL of patients. For the interaction factor the regression coefficient (ß) was −.358. By Cohen's conventions, a combined effect of this magnitude can be considered ‘large’ (f2 = −.256).  Theory of mind but not emotion perception or neurocognition was associated with QoL in patients. |
| Tas et al. 2013 [95] | Research study | III | Cognition: WMS-III, WCST, TMT-B, CPT, RAVLT, BVMT Emotion perception: FEIT, FEDT,  mental state decoding and reasoning: RMET, HINT, UOT,  Attributional style: IPSAQ  QOL, PANSS | 28 SCZ | The quality of Interpersonal Relations was significantly predicted by the negative symptoms and the estimated intelligence. The quality of occupational performance was significantly predicted by estimated intelligence. The quality of Intrapsychic Foundation was predicted by negative symptoms and mental state reasoning. Executive functioning and estimated intelligence predicted the Common Objects and Activities subdomain of QoL.  According to the mediator analysis, social cognition did not show a potential mediating effect among all predictors and QoL subdomains. |
| Hasson-Ohayon et al. 2015 [96] | Cross sectional  Research study | III | FEIT, Faux-Pas Task, MAS-A, Social QoL Scale | 39 SCZ, 60 HC | Two of the subscales of MAS-A, Understanding of Others' Minds and Self Reflectivity were significant predictors of SQoL (F(4,94) = 25.01, p b .001, R2 = .52). Higher MAS-A Understanding of Others' Minds and lower MAS-A Self-Reflectivity scores were associated with better social quality of life. |
| Martin Contero et al. 2017 [97] | Research study | III | IRI, GEOPTE scale of social cognition for psychosis, WHOQOL-BREF | 41 SCZ | The GEOPTE scale showed an inverse relation with all dimensions of QoL |

**BVMT:** Benton Visual Memory Test; **CPT**: Continuous Performance Test; **CVLT**: California Verbal Learning Test; **FEDT**: Face Emotion Discrimination Task; **FEIT**: Facial Emotion Identification Test; **GEOPTE**: Grupo Espanol para la Optimizacion del Tratamiento de la Esquizofrenia; **HC**: healthy controls; **HINT**: Hinting Task; **IPSA-Q**: Internal, Personal and Situational Attributions Questionnaire; **IRI:** Interpersonal Reactivity Index; **IQ**: intelligence quotient; **MAS**: Metacognition Assessment Scale; **PANSS**: Positive and Negative Syndrome Scale; **PCET:** Penn Conditional Exclusion Test; **PCPT**: Penn Continuous Performance Test; **PEAT**: Penn Emotion Acuitiy Test; **PSI**: Processing Speed Index; **QoL**: Quality of Life; **RAVLT**: Rey auditory-verbal learning test; **RMET**: Reading the Mind in the Eyes Test; **SCZ**: subjects with schizophrenia; **SWL**: Satisfaction With Life; **TMT-B**: Trail Making Test-B; **UOT:** Unexpected Outcome Task; **WAIS:** Wechsler Adult Intelligence Scale; **WCST:** Wisconsin Card Sorting Test; **WHOQOL-BREF**: World Health Organization Quality-of-Life Scale; **WMS**: Wechsler Memory Scale; **WMS-R**: Wechsler Memory Scale-Revised.**Table e6.** Available validated instruments for neurocognition in schizophrenia**.**

| **Instrument** | **Subtests / Number of items** | **Domains** | **Administration time** | **Language** |
| --- | --- | --- | --- | --- |
| **Performance-based instruments** | | | |  |
| BACS [32] | 1-List learning (Verbal memory);  2-Digit sequencing task (Working memory);  3-Token motor task (Motor speed);  4- Category instances and COWA test (Verbal fluency);  5-Tower of London (Reasoning/problem solving);  6-Symbol coding (Attention/vigilance and Speed of processing) | Verbal memory; Working Memory; Motor speed; Verbal fluency; Attention and Speed of processing; Reasoning/problem solving | ~35 minutes | English [32]; Chinese [98]; German [99]; French [100]; Spanish [101]; Italian [102]; Japanese [103]; Persian [104]; Brazilian Portuguese [105]. |
| SCIP [106] | 1-Verbal learning test-Immediate;  2-Verbal Learning Test-Delayed;  3-Working memory test;  4-Verbal fluency test;  5-Processing Speed Test | Speed of processing; Attention; Verbal fluency; Verbal memory | ~15 minutes | English; Danish; Dutch; French; German; Italian; Spanish [106] |
| MCCB [107] | 1-BACS: Symbol Coding;  2-Category Fluency: Animal Naming;  3-Trail Making Test: Part A;  4-Continous Performance Test, identical pairs version;  5-Wechsler Memory Scale-Revised III: Spatial Span;  6-Letter Number Span;  7-Hopkins Verbal Learning Test-Revised;  8-Brief Visuospatial Memory Test-Revised;  9-Neuropsychological Assessment Battery: Mazes;  10-Mayer-Salovey-Caruso Emotional Intelligence Test: Managing emotions branch | Speed of processing; Attention/ vigilance; Working memory; Verbal memory & learning; Visual memory & learning; Reasoning/problem solving; Social cognition | 60-90 minutes | English; Traditional Chinese; Simplified Chinese; Croatian; Dutch; French; German; Hebrew; Hindi; Italian; Japanese; Kannada; Korean; Marathi; Polish; Brazilian Portuguese; Romanian; Russian; Serbian; Spanish, Central & South America; Spanish, Spain; Tamil; Telugu; Ukrainian. |
| B-CATS [108] | 1-Trail Making test part A;  2-Trail Making test part B: Digit symbol substitution;  3-Category Fluency: Animal Naming | Speed of processing | ~10 minutes | English [109] |
| BNA [108] | 1-Wechsler Adult Intelligence Scale-Revised Digit symbol test;  2-Letter-number sequencing test | Speed of processing; Working memory | ~10 minutes | English [108] |
| **Interview-based instruments** | | | |  |
| SCoRS [110] | 18 items | Speed of processing; Attention/vigilance; working memory; reasoning/problem solving; memory; language production | 20-30 minutes | English; Professional translations: Bulgarian; Traditional Chinese; Simplified Chinese; Czech; Dutch; French (FR, CA); German; Italian; Japanese; Korean; Polish; Romanian; Russian; Spanish; Ukrainian. Academic translations: Greek; Iranian; Brazilian Portuguese; Serbian; Turkish [111] |
| CGI-CogS [112] | 38 items;  Two categories:  1-Activities of daily living;  2-Neurocognitive state | Speed of processing; Attention/ vigilance; Working memory; Verbal memory & learning; Visual memory & learning; Reasoning/problem solving; Social cognition | ~30 minutes | English [112] |
| CAI [113] | 10 items | Speed of processing; Attention/vigilance; Working memory; Verbal memory & learning; Reasoning/problem solving; Social cognition | ~15 minutes | English [113]; Italian [114]; Turkish [115]; Spanish [116] |

**BACS**: Brief Assessment of Cognition in Schizophrenia; **B-CATS**: Brief Cognitive Assessment Tool for Schizophrenia; **BNA**: Brief Neurocognitive Assessment; **CAI**: Cognitive Assessment Interview; **CGI-CogS**: Clinical Global Impression of Cognition in Schizophrenia; **COWA test**: Controlled oral word association test.; **MCCB:** MATRICS Cognitive Consensus Battery; **SCIP**: Screen for Cognitive Impairment in Psychiatry; **SCoRS**: Schizophrenia Cognition Rating Scale.

| **Table e7.** Psychometric properties of available validated instruments to assess neurocognition in schizophrenia in chronological order | | | | | | |
| --- | --- | --- | --- | --- | --- | --- |
| **Article** | **Type of study** | **Level of evidence** | | **Instruments** | **Sample Size** | **Results** |
| **Performance-based instruments** | | | | | | |
| Keefe et al., 2004 [32] | Instrument development  Test selection and psychometric properties of BACS and comparison with a standard neurocognitive battery | III | | BACS  Neurocognitive standard battery (e.g., RAVLT; Reading subtest; TMT; AX-CPT; WMS) | 150 SCZ and 50 HC | BACS demonstrated high reliability and concurrent validity with a standard battery of tests in SCZ and HCs with similar ages, racial backgrounds and parental education. BACS demonstrated good test–retest reliability in SCZ and HCs. The composite scores from the BACS and the standard battery were highly correlated in patients and in controls, and neither battery was more sensitive to the overall deficits found in patients with schizophrenia. The magnitude of these deficits, which was about 1.5 standard deviations below the healthy controls, were consistent. |
| Keefe et al., 2006 [34] | Research study-Instrument validation | III | | BACS  ILSI  UPSA | 60 SCZ | BACS composite scores were significantly correlated with functional capacity (r = .65, df = 55, p < .001), and real-world functional outcome (r = .37, df = 56, p = .005). In multiple regression analyses, UPSA scores did not account for additional variance in real-world functioning beyond that accounted for by the BACS. |
| Bralet et al., 2007 [100] | Research study-Instrument validation of the French version of the BACS | III | | BACS  Neurocognitive standard battery (e.g., RAVLT; TMT; WAIS; Verbal Fluency;  Wisconsin  Card Sort Test) | 50 SCZ | All the patients completed each of the subtests of the French BACS. The mean duration of completion for the BACS French version was 36 min (S.D. ¼ 5.56). BACS global score correlated with the  standard battery global score showed t (r= 0.81, p < 0.0001). Significant correlations were found between BACS sub-scores  and the standard battery sub-scores of verbal memory, working memory, verbal fluency, attention and speed of information  processing, executive functions (p < 0.001) and motor speed (p < 0.05). |
| Kaneda et al., 2007 [103] | Research study-Instrument validation and psychometric properties of the Japanese version of the BACS  (BACS-J) | III | | BACS | 30 SCZ | Cronbach's alpha was 0.77. The BACS‐J composite score was significantly correlated with all primary measures of BACS‐J (verbal memory, working memory, motor speed, verbal fluency, attention, and executive function). All BACS‐J primary measures and the composite score were significantly correlated between two assessments. The mean score of the Digit Sequencing Task and composite score on the second assessment were significantly larger than those on the first assessment. All BACS‐J primary measures except the Symbol Coding Task were significantly correlated with relevant standard neurocognitive tests. Also, the BACS‐J composite score was significantly correlated with all standard neurocognitive tests except the Continuous Performance Test. |
| Salgado et al., 2007 [117] | Research study-Instrument validation and psychometric properties of the Brazilian version of the BACS | III | | BACS | 20 SCZ and 20 HC | All of the measures on the Brazilian BACS demonstrated significant differences between controls and patients (P<0.01).  Test-retest reliability:  The Cronbach’s α value was 0.89.  Practicality and tolerability:  The Brazilian-BACS required a mean ±SD 43.4±8.4 minutes for patients and a mean ±SD 40.5±5.7 minutes for controls (p=0.17). |
| Anselmetti et al., 2008 [102] | Research study-Co-normalization of Italian BACS scores | III | | BACS | 204 HC | Performance on the BACS was influenced by age and education. In particular, these factors had a significant effect on almost all variables, with the exception of the verbal fluency which was influenced only by education and not age. |
| Chianetta et al., 2008 [118] | Research study-Psychometric properties of BACS and RBANS | III | | BACS  RBANS | 36 SCZ and 14 HC | Both instruments were easily administrable. Internal consistency was satisfying (global scale reliability alphas of 0.90 for the BACS, and 0.87 for the RBANS), although some sub-scores from the RBANS decreased the overall consistency of the instrument. BACS and RBANS composite scores were highly correlated to verbal, non-verbal and total WAIS-III scores (BACS: r=0.727, 0.865 and 0.857, respectively; RBANS: r=0.843, 0.747 and 0.875, respectively). SCZ underperformed HC by a magnitude of 1.81 SD (BACS), and 0.78 SD (RBANS), after adjusting for education. Both batteries showed good test-retest reliability, except for three sub-scores from the RBANS (Picture naming, Digit span, List recognition task). |
| Keefe et al., 2008 [119] | Research study-  Co- normalization and standardization of the BACS | II | | BACS | 404 SCZ | The six subdomains demonstrated the expected pattern of correlations with age, gender, and education. Individual test scores were converted into standardized (T and z) scores and composite scores that were corrected for age and gender. |
| Kern et al., 2008 [120] | Research study- Co-normalization and standardization of the MCCB | III | | MCCB | 300 HC | Prominent age and education effects were observed across tests. The results for gender differed by measure, suggesting the need for age and gender corrections in clinical trials. |
| Nuechterlein et al., 2008 [107] | Instrument development  Test selection, reliability and validity of MCCB | II | | MCCB | 176 SCZ and 167 of them 4 weeks later | The expert panel evaluated more than 90 tests in seven cognitive domains to identify the 36 most promising measures. A separate expert panel evaluated the degree to which each test met specific selection criteria. Twenty tests were selected as a beta battery. The beta battery was administered to 176 SCZ and re-administered to 167 of them 4 weeks later so that the 20 tests could be compared directly.  Based on data of test-retest reliability, practice effects, relationships to functional status, practicality, and tolerability 10 tests (see Table 2.1) were selected to represent seven cognitive domains (see Table 2.1) in the MCCB. |
| Pino et al., 2008 [121] | Research study-  Validation and of the Spanish version of the SCIP | III | | SCIP | 126 SCZ and 39 HC | Average time for SCIP administration was 16.02 (SD = 5.01) minutes. Test–retest reliability intra-class correlation coefficients ranged from 0.74 to 0.90, with an internal consistency Cronbach's alpha value of 0.73. The three parallel forms of SCIP were shown to be equivalent. The SCIP scales were correlated with corresponding neuropsychological instruments, with Pearson's r between 0.38 and 0.60, p< 0.01. The SCIP effectively discriminated between the patient and control samples. |
| Hurford et al., 2011 [122] | Instrument development  Test selection of B-CATS | III | | B-CATS | FES study (73 SCZ), CHOR study (56 SCZ) and CATIE study (1005 SCZ) | The composite score from the three subtests (see Table 2.1) correlated .86 with the global score of the larger battery in 2 of the studies and correlated between .73 and .82 with the total battery scores excluding these 3 tests.  Evaluating subtests from three batteries employed in FES, CHOR and CATIE studies, three were selected. B-CATS is a short-administration time battery, reliable and useful for clinicians; however, concordance with the five criteria selected by MATRICS was not tested. |
| Keefe et al, 2011 [123] | Clinical trial- Psychometric properties of the MCCB | II | | MCCB | 323 SCZ | The test-retest reliability of the MCCB composite score was excellent (intraclass correlation coefficient=0.88). The severity of cognitive impairment was T=24.7 (SD=12.1) at screening and T=26.7 (SD=12.4) at baseline. The MCCB composite score demonstrated a large correlation with the UPSA-B composite score (r=.60, df=304, p<.001). The practice effect on the composite score was small (z=0.18). |
| Sachs et al., 2011 [99] | Research study-Instrument validation and psychometric properties of the German version of the BACS | III | | BACS | 30 SCZ and 30 HC | The German version of the BACS showed high test-retest reliability. Sensitivity and specificity scores  demonstrated good ability to differentiate between patients and controls. |
| Segarra et al., 2011 [101] | Research study-Instrument validation and psychometric properties of the Spanish version of the BACS | III | | BACS | 117 SCZ and 37 HC | All BACS cognitive subtests discriminated between patients and controls (P < .001), and the concurrent validity between the BACS and a traditional neuropsychological test battery was similar to that reported in other languages. |
| August et al., 2012 [124] | Research study-  Assessment of clinical and cognitive correlates of the MCCB | III | | MCCB | 177 SCZ and 77 HC | The MCCB was highly sensitive to the type and level of impairment typically observed in schizophrenia; the MCCB composite score was highly correlated with WASI Estimated Full Scale IQ score; the MCCB domain scores were generally moderately-highly intercorrelated; MCCB performance was minimally related to clinical symptom type and severity; the MCCB is sensitive to employment status with better performance in employed vs. unemployed patients. |
| Mohn et al., 2012 [125] | Research study-Co-normalization of Norwegian version of MCCB | III | | MCCB | 250 HC Norwegian men and women between the ages of 12 and 59 years | Women performed better than men on Speed of Processing and Verbal Learning, and men outperformed women on Attention/Vigilance and Reasoning/Problem Solving tasks. There were substantial reductions in most domains of cognitive function with increasing age. The effect of education on cognitive performance was more modest. |
| Rodriguez-Jimenez et al., 2012 [126] | Research study-Co-normalization of Spanish version of MCCB | III | | MCCB | 210 HC | Findings indicate significant age, gender, and education effects on the normative data for the MCCB in Spain, which are comparable to those effects described for the original standardized English version in the U.S. |
| Eng et al., 2013 [127] | Research study-Co-normalization of the BACS in english-speaking Chinese sample | III | | BACS | 595 HC english-speakers | Notable differences in subtest performances were found against a Western comparison sample. The overall performance of Singaporeans, compared to US populations, was poorer in all subtests except the Spatial Span subtest in which no mean differences across samples were detected |
| Kaneda et al., 2013 [128] | Research study-  Validation and psychometric properties of the Japanese version of MCCB | III | | MCCB | 37 SCZ | Cronbach's alpha for the MCCB-J was 0.72. The MCCB-J composite score was significantly correlated with all subtests of the MCCB-J. There was a significant correlation between the MCCB-J and the BACS composite score. This preliminary study indicates that the MCCB-J has good psychometric properties and validity. |
| Rapisarda et al., 2013 [129] | Research study-Co-normalization of the MCCB in Asian countries | III | | MCCB | 171 HC | Although generally lower when compared to the US norming sample, Singaporean scores reflected the same relationship with age, education, and gender, with the exception of a substantially worse performance in the social cognition domain. |
| Fervaha et al., 2014 [108] | Instrument development  Test selection and psychometric properties of BNA | II | | BNA | 1303 SCZ | The BNA explained 76% of the variance in global neurocognition in the total sample and remained consistent in subsamples stratified by clinical characteristics (e.g., severity of psychopathology) and in randomized re-sampling simulations.  BNA is easy to administer, valid and correlates with functioning and symptoms; however, concordance with the five criteria selected by MATRICS was not tested. |
| Mazhari et al., 2014 [104] | Research study-  Validation and psychometric properties of the Persian version of the BACS | III | | BACS | 50 SCZ and 50 HC | Cronbach’s alpha for the Persian-BACS was 0.74. All the Persian-BACS subscales were significantly correlated with the corresponding standard neurocognitive subscales and the Pearson correlation of the composite scores from the two instruments was 0.71. Moreover, a one-factor solution was found that accounted for 67.9% of the variance. Finally, the Persian-BACS demonstrated high ability to discriminate patients with schizophrenia from healthy controls. Good psychometric properties of the Persian-BACS suggest that it is a useful tool for assessing cognition in schizophrenic patients with Persian as their primary language. |
| Roseberry et al., 2014 [130] | Research study-  Assessment of practice effects and expectation for change over time for the MCCB | III | | MCCB | 27 SCZ and 29 HC | A significant main effect was observed for time; no significant interactions (time x diagnosis) were observed. There was no support for differential practice effects. In the absence of any behavioral, cognitive, or pharmaceutical interventions, these findings suggest limited change in performance over time in either group. |
| Araujo et al., 2015 [105] | Research study-  Validation and psychometric properties of the Portuguese version of the  BACS | III | | BACS | 116 SCZ and 58 HC | SCZ performed significantly worse than the HC (p<0.001) on all subtests of the BACS and on the total score, which attests to the discriminant validity of the test. The global score of BACS was significantly correlated with all of the subtests and with the global score for the standard battery. The BACS also had good test-retest reliability (rho>0.8) and high internal consistency (Cronbach's α ϝ 0.874). |
| Fervaha et al., 2015 [131] | Research study-  Instrument validation  Psychometric properties of BNA and comparison with MCCB | III | | BNA  MCCB | 176 SCZ and 300 HC | The BNA was highly correlated with global cognition as evaluated by the MCCB in both the SCZ (r=0.82) and HC (r=0.75). Both instruments were similarly sensitive to deficits in global cognition. |
| Jędrasik-Styła et al., 2015 [132] | Research study-  Validation and of the Polish version of the MCCB | III | | MCCB | 61 SCZ | All tests in the battery were found to have high test-retest reliability.  All the tests were rated as tolerable and practical by patients and administrators. However  practice effects were generally higher in the Polish version of the MCCB than in the  original version. |
| Lees et al., 2015 [133] | Research study-  Cross-validation of MCCB  and CogState in schizophrenia | III | | MCCB and CogState | 143 SCZ participating to three studies (Each study administered MCCB and CogState  tests on consecutive days (baseline 1 and 2) and follow-up 3–  4 weeks later) | Many MCCB tasks and domains (and CogState social cognition  and perhaps maze tasks) displayed appreciable initial practice effects. The MCCB tasks had better test-retest reliability but  there was little difference in the CogState composites’ and MCCB domains’ reliability. In experiments involving different ethnicities, or measuring change, CogState composite scores appear to be more useful, especially if repetition of baseline to reduce learning effects is undesirable. However, the MCCB tasks  correlated a little better with social skills measures,  underlining their validity, and some Cogstate-Schizophrenia Battery tasks were more sensitive to smoking’s effects. |
| Shi et al., 2015 [134] | Research study-  Co- normalization of the MCCB in China | III | | MCCB | 656 HC | Age, gender, and education had significant effects on the normative data for MCCB in China, which are comparable to those found for the original standardized English version in the U.S. and the Spanish version in Spain. Remarkably, the residence scale effects on neuropsychological performance were significant, which should be taking into account when calculating the standardized T score for each subject. The practice effects were minor and test-retest reliability of MCCB was good, which suggests MCCB as an appropriate measure for clinical and research usage in China. |
| John et al., 2016 [135] | Research study | III | | BACS | 122 SCZ | The mean composite score on the BACS was 1.8 standard deviations below the norm, and 43% had moderate or severe cognitive deficits. The BACS sub-tests of list learning and symbol coding revealed more severe deficits. |
| Kumar et al., 2016 [136] | Research study | III | | MCCB | 60 SCZ and 30 HC | Relationship to functioning:  Most of the MCCB domains and subtests, correlated with UPSA total score. Performance on MCCB was strongly associated with functional capacity in older patients with schizophrenia. |
| Wang et al., 2016 [98] | Research study-  Validation and psychometric properties of the BACS in mandarin-speaking Chinese sample | III | | BACS | 66 SCZ and 66 HC | The BACS had good test–retest reliability, and all BACS subtests had statistically insignificant practice effects. Principal components analysis demonstrated that a one-factor solution best fits our dataset (60.9% of the variance). In both patients and controls, the BACS composite scores were positively correlated with all BACS subscales and UPSA-B scales. Furthermore, all BACS subtests significantly differentiated SCZ from HC and the BACS composite score had the best discriminative validity. |
| Fonseca et al., 2017 [137] | Research study-  Co- normalization and validation of the MCCB in Brazil | III | | MCCB | Pilot study (30 HC)  Validation study (99 SCZ and 99 HC) | The results showed adequate to high levels of baseline and 4-week retest reliability, except the MSCEIT-ME; adequate internal consistency for the MSCEIT-ME for the total sample and patients group, and moderate Alpha for the health control sample; as well as evidence of convergent validity and sensitivity to differentiate performance between the groups. All the 10 MCCB measures showed the lowest learning effects. Overall, the Brazilian version of the MCCB showed similar results to the original North American version. Findings provide reassurance that the MCCB is a reliable and valid measure of cognition across different countries and cultures, which is especially important to the ongoing work in attempting to discover cognition enhancing drugs and the effects of cognitive interventions for the treatment of schizophrenia. |
| Georgiades et al., 2017 [138] | Research study-  Psychometrics properties of the MCCB | II | | MCCB | 2162 SCZ | Test-retest reliability was high (intraclass correlation coefficient=0.88) for both composite scores (cognitive composite score and neurocognitive composite score, which excludes Social cognition). Practice effects were small for the cognitive (d=0.15) and neurocognitive (d=0.17) composites. Simulated bootstrap regression analyses revealed that 3 (Speed of Processing, Working Memory, and Visual Learning) of the 7 domains explained 86% of the variance for both composite scores. |
| Wang et al., 2017 [139] | Research study-  Co- normalization of the BACS in mandarine-speaking Chinese sample | III | | BACS | 382 mandarin-speakers HC | The raw scores of all the BACS tests (verbal memory, digit sequencing, token motor test, verbal fluency, symbol coding, and Tower of London) were negatively correlated with participants’ age. Women were superior to men in verbal memory, but inferior to them in executive function. Furthermore, applying the U.S. norms of the BACS to determine the performance of the Chinese BACS results in bias with regard to verbal memory, token motor test, verbal fluency, symbol coding, Tower of London and composite score. These findings demonstrate that directly applying western cognitive norms to a Mandarin-speaking population can cause biased interpretations. |
| Gòmez-Benito et al., 2018 [140] | Research study-  Validation and of the Spanish version of the SCIP | II | | SCIP | 376 SCZ | The person reliability was .72, separation was 1.61, and the standard error of person mean was .07. The item reliability and separation statistics reached values of .98 and 6.77, respectively, whereas the raw score reliability (Cronbach's alpha) was .75. Taken together, these results indicate that the SCIP‐S is a reliable test and that its items are able to assess different levels of the construct being measured. The analysis also showed that the item location values present a narrow range (−0.79 to 0.63 logits), whereas person ability covered a wide range (−3.59 to 3.65 logits). |
| Hurford et al., 2018 [109] | Research study-  Psychometric properties of B-CATS and comparison with MCCB | III | | B-CATS  MCCB | 91 SCZ | The B-CATS demonstrates good test-retest reliability and internal consistency. It correlates 0.76 (p<0.01) with the MATRICS battery. The B-CATS and the MCCB correlated with the UPSA-B at 0.50 and 0.58 respectively. |
| Mucci et al., 2018 [141] | Research study-  Validation of the Italian version of the MCCB | II | | MCCB | 852 SCZ, 342 unaffected relatives (REL) and of 774 HC | SCZ were significantly impaired on all MCCB domains. REL had intermediate scores between SCZ and HC, showing a similar pattern of impairment, except for social cognition. Proband’s scores significantly predicted unaffected relatives MCCB scores on all domains except for visual learning. |
| Belvedere-Murri et al., 2020 [142] | Research study-  Validation of the SCIP and comparison to MoCA | II | | SCIP | 106 SCZ and 86 HC | The SCIP yielded an adequate level of internal consistency (Cronbach's alpha 0.82). The SCIP total score demonstrated high convergent validity with the RBANS Total (R = 0.71). Most of the SCIP subtests also showed acceptable convergence on the relevant  RBANS or TMT subscale scores (R N 0.50) with the exception of the WMT (R = 0.44). The SCIP Total scores demonstrated a large effect size difference (g = 1.42) between the two groups. Individual SCIP subscales also showed consistent deficits between the two groups with large effect sizes ranging from g = 0.76 on the WMT to g = 1.32 on the VLD-D. Receiver Operator Curve analysis to evaluate the accuracy of SCIP to discriminate between cognitively impaired and non-impaired subjects yielded acceptable AUC values (N0.7) for the VLT-I and WMT domains,  and excellent AUC values (N0.80) for the remainder of the domains and the total score. A SCIP total score lower than 70 provided the optimal cut-off to detect global cognitive impairment with a sensitivity of 76% and a specificity of 83%. |
| Bezdicek et al. 2020 [143] | Research study-Validation, psychometric properties and  Co- normalization of the MCCB in Czech Republic | III | | MCCB | 220 HC  67 SCZ | The internal consistency of all 10 tests in the MCCB battery was good (Cronbach's α = 0.85 (95% CI [0.83, 0.88])). The discriminative validity for the detection of neurocognitive dysfunction in schizophrenia based on the area under the curve of MCCB composite T-score was ≥90% (95% CI [0.85, 0.96]) and all MCCB domains showed p< .001. The MCCB global composite and the Speed of Processing domain score significantly predicted the PSP ratings. |
| **Interview-based instruments** | | | | | | |
| Keefe et al., 2006 [110] | Instrument development  SCoRS item selection and correlation to neurocognition (BACS), daily functioning (ILSI) and functional capacity (UPSA) | III | SCoRS  BACS  ILSI  UPSA | | 60 SCZ and care-givers | -The SCoRS global ratings were significantly correlated with composite scores of cognitive performance (BACS) and functional capacity (UPSA) and with ratings of real-world functioning (ILSI).  - Multiple regression analyses suggested that SCoRS global ratings predicted unique variance in real-world functioning beyond that predicted by the performance measures. |
| Green et al., 2008 [144] | MATRICS Psychometric and Standardization Study-  Assessment of co-primary measures of cognition according to MATRICS | II | SCoRS  CGI-Cogs  MASC  UPSA | | 176 SCZ and 300 HC | Psychometric properties (test-retest reliability, utility as a repeated measure, relationship to cognitive performance, relationship to functioning, tolerability/practicability) of the measures were considered acceptable, and the measures were generally comparable across the various criteria. |
| Ventura et al., 2008 [112] | Research study-  Validation and psychometric properties of CGI-CogS | III | CGI-CogS  Cogtest (CPT AX-version, Spatial Working Memory Test, Auditory Digit Span, Auditory Number Sequencing, Go/No–Go Test, Set Shifting Test, Strategic Target Detection Test, Symbol Digit Substitution Test, Word List Memory Test, and Face Memory Test) | | 35 SCZ and care-givers | The CGI-CogS ratings generally showed a high level of internal consistency (Cronbach's alpha=.69 to .96), adequate levels of inter-rater reliability (ICC's=.71 to .80), and high test-retest stability (ICC's=.92 to .95). Correlations of caregiver and rater global (but not "patient only rating") CGI-CogS ratings with neurocognitive performance were in the moderate range (r's=-.27 to -.48), while most of the correlations with functional outcome were moderate to high (r's=-.41 to -.72). |
| Chia et al., 2010 [8] | Research study-Validation and psychometric properties of SCoRS in Singapore | III | SCoRS | | 103 SCZ and 48 HC | SCoRS is highly reliable (ICC=0.984) and sensitive to cognitive dysfunction. ScoRS is significantly correlated with BACS composite scores and predicted functional outcomes as measured by GAF and WHO-QOL within an Asian population. |
| Ventura et al., 2010 [113] | Instrument development  CAI item selection and correlation with CGI-CogS and ScoRS and with neurocognition (MATRICS), functional outcome (SFS) and functional capacity (UPSA) | II | CAI  CGI-CogS  ScoRS  MATRICS  UPSA  SFS | | 176 SCZ | The rater’s score from the newly derived CAI (10 items) correlated highly (r=.87) with those from the combined set of the ScoRS and CGI-CogS (41 items). Both the patient (r=.82) and the informant (r=.95) data were highly correlated with the rater’s score. The CAI was modestly correlated with objectively measured neurocognition (r=-.32), functional capacity (r=-.44), and functional outcome (r=-.32). |
| Harvey et al., 2011 [145] | Clinical trial | II | SCoRS  MCCB | | 301 SCZ e randomized to 21 days of double-blind treatment with lurasidone  (N= 150) or ziprasidone (N=151) | Utility as a repeated measure:  Lurasidone patients demonstrated significant within group-improvement from baseline on the MCCB composite score (p = 0.026) and on the SCoRS (p < 0.001), but ziprasidone patients did not improve on either the MCCB composite (p = 0.254) or the SCoRS (p = 0.185). At endpoint there was a statistical trend (p = 0.058) for lurasidone to demonstrate greater improvement from baseline in SCoRS ratings. Improvements in interview-based aspects of cognition were not related to MCCB test changes and had minimal correlations with changes in symptoms. These data suggest that interview-based cognitive measures such as the SCoRS may be sensitive to changes after 3 weeks of treatment in patients with schizophrenia. |
| Gonzalez et al., 2013 [146] | Cross-cultural study-  Co-primary measures of cognition are compared in different cultural settings | III | CAI  ILSI  UPSA  TABS | | 55 participants (principal investigation and research assistant in 31 sites of 8 countries) | Among all measures compared (ILSI, UPSA, TABS, CAI) only CAI required minor cultural adaptation; the other measures required greater cultural adaptation. |
| Ventura et al., 2013 [147] | Research study-  Validation and psychometric properties of the CAI | II | CAI | | 150 SCZ | The CAI had good internal consistency (Cronbach’s alpha = .92) and good test-retest reliability (*r* = .83). The CAI was moderately correlated with objective neurocognitive test scores (*r*= −.39 to −.41) and moderately correlated with social functioning (*r* = −.38), work functioning (*r* = −.48), and overall functional outcome (*r* = −.49). The correlations of CAI scores with external validity indicators did not differ significantly by source of information (patient alone ratings were valid). Overall functional outcome correlated more strongly with patient CAI scores (*r* = −.50) than with objective neurocognitive test scores (*r* = .29) or functional capacity (*r* = .29). |
| Vita et al., 2013 [148] | Cross-sectional study-  Validation of the ScoRS in different settings (stable and recently hospitalized SCZ) in an Italian population | III | ScoRS | | 59 stable SCZ and 27 recently hospitalized SCZ | ScoRS inter-rater and test-retest reliability were high. In clinically stabilized patients, ScoRS global ratings were significantly correlated with composite scores of cognitive performance (global cognitive index: r = -0.570, P<0.001), symptoms (PANSS total score: r = 0.602, P < 0.001), and psychosocial functioning (GAF): r = -0.532, P<0.001; (HoNOS): r = 0.433, P < 0.001). On the other hand, no such correlations were found in recently hospitalized patients. Correlations with neuropsychological and functional measures were less significant as the severity of the patients’ symptoms, especially positive symptoms, increased. |
| Bosgelmez et al., 2015 [115] | Research study-  Validation and psychometric properties of Turkish version of the CAI (CAI-TR) | III | CAI (CAI-TR) | | 90 SCZ | Internal consistency of CAI-TR was good, with Cronbach’s alpha value of 0.97. For patient scores, Cronbach’s alpha value was 0.91. Each item of CAI-TR was correlated with the related neurocognitive test (r=0.242-0.564; p<0.05). Moreover, overall scores of CAI-TR showed statistically significant correlations with GAF (r=-0.538, p<0.001), social functioning (r=-0.520; p<0.01), and objective neurocognitive tests. As a measure of external validity of CAI-TR, statistically significant correlations were determined between patient, informant and interviewer evaluations independent of source of information (r=0.707, r=0.830, r=0.835, respectively; p <0.001 for all). Mean duration of patient interview was 18.7 minutes (8-30 min; SD=5.4), mean duration of informant interview was 18.0 minutes (10-25 min; SD=5.0) and total mean duration of CAI administration was 36. 6 minutes (18-55 min; SD=9.7). |
| Keefe et al., 2015 [149] | Clinical Trial- Psychometric properties of the ScoRS | III | ScoRS | | 79 SCZ and 319 SCZ (+ informants) | The ScoRS interviewer ratings demonstrated excellent test-retest reliability in several different circumstances, including those that did not involve treatment (ICC> 0.90), and during treatment (ICC>0.80). ScoRS interviewer ratings were related to cognitive performance as measured by the MCCB (r= −0.35) and demonstrated significant sensitivity to treatment with encenicline compared to placebo (p<.001). ScoRS has potential as a clinically relevant measure in clinical trials aiming to improve cognition in schizophrenia and may be useful for clinical practice. The weaknesses of the ScoRS include its reliance on informant information, which is not available for some patients, and reduced validity when patient self-report is the sole information source. |
| Sànchez-Torres et al., 2016 [116] | Research study-  Validation and psychometric properties of the Spanish version of the CAI (CAI-Sp) | III | (CAI-Sp) | | 81 SCZ and 31 HC | Good internal consistency was found for the CAI-Sp patient, informant and rater scores in patients (Cronbach’s alpha 0.87, 0.94 and 0.95).  Poorer cognitive functioning as assessed with the CAI-Sp was associated to illness severity, specifically positive, negative and disorganised syndromes. Binary logistic regression showed that the CAI-Sp was able to detect cognitive impairment in patients, when considering CAI-Sp patient and informant information and CAI-Sp rater scores.  Good internal consistency was found for the CAI-Sp patient, informant and rater scores in patients (Cronbach’s alpha 0.87, 0.94 and 0.95) |
| Higuchi et al., 2017 [150] | Research study-  Validation and psychometric properties of the Japanese version of the SCoRS | III | SCoRS | | 38 FES, 135 SCZ, 102 ARMS and 19 with psychosis | SCoRS scores showed a significant relationship with SOFAS scores for the entire subjects. Also, performance on the BACS was significantly correlated with SCoRS scores. These associations were also noted within each diagnosis (FES, SCZ, ARMS). These results indicate the utility of SCoRS as a measure of functional capacity that is associated both with cognitive function and real-world functional outcome in subjects with schizophrenia-spectrum disorders. |
| Kang et al., 2017 [151] | Research study-  Validation and psychometric properties of the Korean version of SCoRS (SCoRS-K) | III | SCoRS-K | | 84 SCZ and 29 HC | SCoRS-K has high internal consistency (Cronbach’s alpha; patient 0.941, informant 0.905, interviewer 0.964); test-retest reliability [patient 0.428 (p=0.003), informant 0.502 (p<0.001), interviewer 0.602 (p<0.001); and global rating 0.642 (p<0.001)]. The mean scores of subjects were significantly higher than those of the controls (p<0.001), demonstrating SCoRS-K’s discriminant validity. |
| Mazhari et al., 2017 [152] | Research study-  Validation and psychometric properties of the Persian version of the SCoRS | III | SCoRS | | 35 SCZ and 35 HC | Persian version of the SCoRS was sensitive to cognitive impairment in the patients. The Persian SCoRS global rating was significantly associated with the composite score generated from the Persian version of the BACS and predicted functional outcomes as measured by GAF and WHO QOL. |
| Harvey et al., 2019 [111] | Narrative review-Psychometric properties of the SCoRS | III | SCoRS | | 18 publications adopting the SCoRS as a measure of cognition | SCoRS had excellent psychometric properties: practical and easy to administer for a clinician or researcher; validated in SCZ; contains the relevant areas of cognition and functioning applicable to schizophrenia; able to assess all phases and severity levels of schizophrenia; capable of monitoring disease progression; minimal burden to patients; sensitive to assess treatment effects; low burden on both patients and clinician; fits the MATRICS criteria for a co-primary measure of cognition. Versions of the SCoRS have been developed in 22 languages and only little cultural adaptation was required. |
| Palumbo et al., 2019 [114] | Research study- Validation and psychometric properties of the Italian version of the CAI | III | CAI | | 50 SCZ | For the three considered scores (patient, informant and composite scores) of each item of the CAI, as well as for GAF score, the ICCs ranged from 0.69 to 0.91. Cronbach’s alpha coefficients for the CAI patient, informant e composite scores were respectively 0.90, 0.93 and 0.93. The Italian version of the CAI revealed good to excellent reliability and excellent internal consistency. |

**ARMS:** at-risk mental state; **BACS**: Brief Assessment of Cognition in Schizophrenia; **B-CATS**: Brief Cognitive Assessment Tool for Schizophrenia; **BNA**: Brief Neurocognitive Assessment; **CAI**: Cognitive Assessment Interview; **CATIE study**: The Clinical Antipsychotic Trials of Intervention Effectiveness; **CIBIS:** Clinical Interview-Based Impression of Severity; **CIBIC:** Clinical Interview-Based Impression of Change with input from caregivers, **CGI-CogS**: Clinical Global Impression of Cognition in Schizophrenia; **CHOR study:** The clozapine, haloperidol, olanzapine, risperidone study; **CPT**: Continuous Performance Tests; **FES:** first episode of schizophrenia; **GAF**: Global Assessment of Functioning; **HC**: healthy controls; **HoNOS**: Health of the Nation Outcome Scale; **ICC’s**: Intraclass correlation coefficient; **ILSI**: Independent Living Skills Interview; **MASC**: Maryland Assessment of Social Competence; **MCCB**: MATRICS Consensus Cognitive Battery; **MSCEIT-ME:** The Mayer-Salovey-Caruso Emotional Intelligence Test-Managing Emotion task; **PANSS**: Positive and Negative Syndrome Scale; **RAVLT**: Rey Auditory–Verbal Learning Test; **RBANS**: Repeatable Battery for the Assessment of Neuropsychological Status; **SCoRS**: Schizophrenia Cognitive Rating Scale; **SD:** Standard Deviation; **SCZ**: subjects with schizophrenia; **SCWT:** The Stroop Color and Word Test; **SOFAS:** Social and Occupational Functioning Assessment Scale, **SFS**: Social Functioning Scale; **TABS**: Test for Adaptive Behaviour in Schizophrenia; **TMT:** Trail Making Test; **UPSA**: University of California Performance Skills Assessment; **WAIS**: Wechsler Adult Intelligence Scale; **WASI**: Wechsler Abbreviated Scale of Intelligence; **WMS**: Wechsler Memory Scale; **WHO-QLS**: World Health Organization-Quality of Life.

**Table e8.** Available validated instruments for social cognition in schizophrenia.

| **Instruments** | **Type of task** | **Domains** | **Administration time** | **Languages** |
| --- | --- | --- | --- | --- |
| The Hinting Task [153] | It is paper and pencils task devised to test the ability of subjects to infer the true intent of indirect speech utterances. The task comprises ten short passages read aloud presenting an interaction between two characters and involving one of the characters dropping of a hint. The participants are then asked to say what the character dropping the hint intended. If the response, at this stage, is correct, a score of two is given and the next story is read out loud. If the subject fails to give the correct response and a paraphrase of the hint is prompted, the subject is given a score of one. If the subject fails again to infer the intended meaning of both indirect speech utterances, then a score of zero is given for that item. | Theory of mind | 5-7 minutes | English [153];  Dutch [154];  Spanish [155];  Portuguese [156]  Norwegian [157];  Polish [158];  Chinese [159];  Malay [159];  Korean [160];  Japanese [161]. |
| BLERT [162] | It measures the ability of a person to identify affect cues. It is an audio-visual task designed to elicit a person's ability to properly discriminate 7 emotional states (happiness, sadness, fear, disgust, surprise, anger or no emotion) expressed by facial, voice-tonal and upper-body movement of a male actor during 21 ten-second video clips. Performance is scored as the total number of correct responses. Videos are played via computer, scoring via paper and pencil. | Emotional processing | 6-8 minutes | English [162];  Italian [163];  Chinese [159];  Malay [159]; Japanese [161]. |
| The Trustworthiness task [164] | Participants are shown 100 (or 42 in the abbreviated version of the task) faces of unfamiliar people, showing ethnically diverse males and females in natural poses, and are asked to judge how much they would trust the person by providing a rating on a seven-point scale, ranging from -3 (very untrustworthy) to +3 (very trustworthy). In rating trustworthiness, participants are asked to imagine trusting the person in the photograph with all of their money, or with their life and to indicate the degree to which they would trust that person. For approachability, participants are asked to imagine meeting the person on the street, and to indicate how much they would want to walk up to the person and start a conversation. | Attributional bias/style | 4-6 minutes | English [164] |
| The Reading the Mind in the Eyes Test (Eyes Test) [165] | It measures the capacity to discriminate the mental state of others from expressions in the eye region of the face. It includes 36 photographs of male and female eyes, rather than the whole face, depicting emotional states. For each photograph, participants are asked to choose the emotional state that best describes the eyes, choosing between one of four possible emotions. Each stimulus is presented with 4 response options. Participants are awarded one point for each correct item. Total score is calculated by adding the number of correct responses, and scores range from 0-36. | Theory of mind | 6-7 minutes | English [165],  Italian [166],  Korean [167],  Serbian [168],  French [169],  Chinese [159];  Malay [159];  Japanese [161];  Indian [170]. |
| MSCEIT [171] | It is a 141-item scale, made up of eight tasks, measuring 4 branches of emotional intelligence: perceiving emotions (PE), using emotions to facilitate thoughts (FE), understanding emotions (UE), and managing emotions (ME). Each subscale consisted of two tasks. Responses include 5-point Likert ratings with specific anchor points for some items and a 5-item multiple-choice format for others. MSCEIT scores were derived using the general consensus approach based on a large community sample rather than the expert rating approach. MSCEIT-ME represents to date the only measure that assesses emotional regulation, a subdomain of the emotional processing domain. | Emotional processing-emotional regulation | ~35 minutes | English; Traditional Chinese; Simplified Chinese; Croatian; Dutch; French; German; Hebrew; Hindi; Italian; Japanese; Kannada; Korean; Marathi; Polish; Brazilian Portuguese; Romanian; Russian; Serbian; Spanish, Central & South America; Spanish, Spain; Tamil; Telugu; Ukrainian [108]. |
| TASIT [172] | It is comprised of videotaped vignettes of everyday social interactions and includes three sections, each with alternative forms (form A and B). Part I: The Emotion Evaluation Test (EET) which evaluates the ability to recognize the basic emotions expressed by others, in 28 video sequences; the subject is asked to identify the emotion expressed by a character, choosing from 7 options (surprise, happiness, anger, sadness, anxiety, disgust, neutral); Part 2: Social Inference – Minimal (SI-M) comprised 15 vignettes that represented dialogues between two actors and assesses comprehension of sincere versus sarcastic exchanges. Part 3: Social Inference – Enriched (SI-E) comprised 16 vignettes that provided additional information before or after the dialogue of interest to ‘‘set the scene’’ and assesses lies versus sarcasm. Videos are played via computer, scoring via paper and pencil | Emotional processing and  Theory of mind | 30-45 minutes; Part III only 17–19 min | English [172];  Italian [173];  Chinese [159];  Malay [159], Japanese [161]. |
| ER-40 [174] | Computer administered task that assesses facial emotion recognition ability. It includes 40 colour photographs of faces depicting a given emotion (i.e., happiness, sadness, anger, or fear) or a neutral expression. Stimuli are balanced for poser’s gender, age, and ethnicity, and for each emotion category. Participants are instructed to examine a series of faces and identify the expressed emotion from 5 possible choices. | Emotional processing | 3-4 minutes | English [174];  Italian [175];  French [176],  Chinese [159];  Malay [159];  Japanese [161]. |
| AIHQ [177] | This paper and pencil task evaluates hostile social cognitive biases. Participants read 5 hypothetical negative social situations with ambiguous causes and record a reason why the scenario occurred. For each situation, participants rate on Likert scales the intentionality of the other's action (on a 1 to 6 scale), how angry it would make the participant feel (on a 1 to 5 scale), and how much he or she would blame the other (on a 1 to 5 scale). These three items are summed for an overall blame score with higher scores indicating greater blame, perceived intention, and anger. Additionally, participants provide two open-ended responses: an explanation of why the event occurred, and what they would do in response to the event. These open-ended questions are evaluated by two independent raters according to the extent to which the participant interpreted the situation in a hostile manner (hostility bias, rating a hostility index) and the extent to which the individual reports aggression in his or her behavioural response (aggression bias, rating an aggression index). | Attributional bias/style | 5-7 minutes | English [177];  Chinese [159];  Malay [159];  Japanese [161]. |
| RAD [178] | It is a 75-item paper-and-pencil measure of competence in relationship perception. The content and format of RAD are based on relational model theory, which proposes that individuals use their implicit knowledge of four relational models to understand social relationships and predict the behaviour of others. (1) Communal sharing, (2) authority ranking, (3) equality matching and (4) market pricing are the four relational models that govern social behaviour. RAD contains 25 vignettes (15 in the abbreviated version), each involving a differently named male–female dyad. Each vignette is followed by three statements that describe the dyad's interpersonal behaviour in domains of social life. Participants are asked to use what they learned about the dyad from the vignette to indicate whether the behaviours described in the three statements are likely or unlikely to occur by answering “yes” or “no.” | Social perception | 35 minutes (16 minutes for the abbreviated version) | English [178];  Chinese [159];  Malay [159]. |
| SAT-MC [179] | Participants viewed a short animation of geometric shapes enacting a social drama. The animation was shown twice, and participants then answered 19 multiple-choice questions about what happened. | Social perception | ~15 minutes | English [179];  Japanese [161]. |
| Mini-PONS [180] | It is a test of accuracy in decoding interpersonal cues (face, body, and voice tone). Participants were presented sixty-four two-second auditory or visual segments of a Caucasian female exhibiting facial expressions, voice intonations, and/or gestures and had to choose which of two behavioural labels best described the situation. | Social perception | ~15 minutes | English [180];  Chinese [159];  Malay [159];  Spanish [181]. |
| IBT [182] | Computer administered task that assesses the tendency to attribute intentionality to the actions of others. Participants read 24 brief descriptions of ambiguous actions and quickly categorize indicating whether that action occurred ‘on purpose’ or ‘by accident’. The IBT can be scored in various ways, but the simplest is to calculate the percentage of items designated as occurring on purpose, with higher scores indicating greater bias. The IBT includes both slow (5 s to respond) and fast (2,4 s to respond) conditions, and general population participants are significantly more likely in the fast condition to perceive prototypically accidental actions as intentional. | Attributional bias/style | 5-6 minutes | English [182];  Japanese [161]. |

**AIHQ:** The Ambiguous Intentions Hostility Questionnaire; **BLERT**: The Bell Lysaker Emotion Recognition Task; **ER-40**: The Penn Emotion Recognition Task; **IBT**: The Intentional Bias Task; **Mini-PONS**: The Mini Profile of Nonverbal Sensitivity; **MSCEIT**: The Mayer-Salovey-Caruso Emotional Intelligence Test; **RAD**: Relationships Across Domains; **SAT-MC**: The Social Attribution Task—Multiple Choice version; **TASIT**: The Awareness of Social Inference Test.

| **Table e9.** Psychometric properties of available validated instruments to assess social cognition in schizophrenia. | | | | | |
| --- | --- | --- | --- | --- | --- |
| **Article** | **Type of study** | **Level of evidence** | **Instruments** | **Sample Size** | **Results** |
| **Emotional processing** | | | | | |
| **BLERT** | | | | | |
| Bell et al., 1997 [162] | Instrument development  Test selection and psychometric properties of BLERT | III | BLERT | 50 SCZ, 25 SUD and 81 HC | SCZ had poorer performance on the BLERT than SUD or HC.  Accuracies were 92.3% for HC, 77.2 for SUD and 64.8 for SCZ.  Test-retest reliability at 5 months for this new method was r = 0.76, and stability of categorization was very high over 5 months (weighted K = 0.93).  Distributions:  Mean percentage correct were 92.3% for controls, 77.2% for substance abuse, and 64.8% for SCZ. No SDs reported. |
| Bell et al., 2001 [183] | Research study- Psychometric properties of BLERT | III | BLERT | 65 SCZ/SCZaff randomly assigned to NET+WT or WT alone | 20 patients treated with NET+WT (65%) had small or large effect-size improvements compared to 10 patients treated with WT (29%).  12 patients treated with NET+WT (39%) had large effect-size changes compared to 3 patients treated with WT (9%).  Relationship to functioning: The BLERT failed to relate to Social Skills as measured by the Work Behaviour Inventory |
| Bryson et al., 2003 [184] | Research study- Psychometric properties of BLERT | III | BLERT | 96 SCZ/SCZaff | Distributions:  Mean number correct was 12.95 (SD=3.75) out of 21. Thus approx. 61.7% correct. |
| Combs et al., 2004a [185] | Research study- Psychometric properties of BLERT | III | BLERT | 65 SCZ | Distributions:  Mean number correct was 12.1 (SD=4.4) out of 21. Thus approx. 57.6% correct. BLERT and FEIT were found to be very highly correlated r = .85, p < .0001. |
| Wexler et al., 2005 [186] | Research study- Psychometric properties of BLERT | III | BLERT | 145 SCZ/SCZaff randomly assigned to CRT+WT or WT alone | The percentage of CRT + WT subjects with normal scores on the BLERT increased from 35 to 60%.  The percentage of WT patients with normal scores declined from 47 to 42%. |
| Nienow et al., 2006 [187] | Research study- Psychometric properties of BLERT | III | **BLERT**  AIPPS | 56 SCZ | Mean correct: HC 15.00 (SD=2.83) and patients 12.04 (SD=3.71);  Relationship to functioning: r = .31 (p<.05) with AIPSS social problem solving. |
| Pinkham and Penny, 2006 [188] | Research study- Psychometric properties of BLERT | III | **BLERT**  FEIT FEDT  SCST  ToM vignettes Hinting task  Conversation Probe role-play test (CP) | 49 SCZ and 44 HC | **BLERT**  Reliability of the BLERT was good (0.73).  Convergent validity:  *SCZ*:  r=.373 with FEIT, r=.326 with FEDT, r=.418 with ToM vignettes, ns with SCST, Hinting task.  *HC*:  ns with FEIT; r=.476 with FEDT; ns with SCST; r=.387 with SCST time, ns with Hinting task and ToM vignettes.  Discriminant validity:  SCZ: Neurocognitive tasks: r=.47 with WRAT, ns with TMT-A, TMT-B. *HC*: Neurocognitive tasks: r=.49 with WRAT, ns with Immediate Memory, ns with Trails A, r=-.44 with Trails B.  Relationship to functioning r=.368 (p<.05) with interpersonal skill (Conversation probe role play) in HC, and r=.38 (p<.01) with interpersonal skill in patients. |
| Bell et al., 2009 [189] | Research study- Psychometric properties of BLERT | II | BLERT | 151 SZ | Convergent validity:  r = .17 with Hinting Task,  Discriminant validity:  r=.18 with WCST, r=.17 with digit span test. All correlations significant at p<.05.  Relationship to functioning: No significant correlations with perceived social discomfort at work, or composite rehabilitation outcomes. |
| Roberts et al., 2009 [190] | Research study- Psychometric properties of BLERT | III | **BLERT**  TASIT  AIHQ | 31 SCZ | **BLERT**  Reliability of the BLERT was good (.77).  Utility as a Repeated Measure:  SCIT effect size d = .29; TAU effect size d = -.19. |
| Bell et al., 2010 [179] | Research study- Psychometric properties of BLERT | III | **BLERT**  SAT-MC  Hinting task  BORRTI  MSCEIT | 66 patients (49 SCZ, 16 SCZaff, 1 Psychosis NOS); 85 HC | **BLERT**  Convergent validity: Correlated significantly (r=.37, p = .0002) with the Social Attribution Test-Multiple Choice (SAT-MC). |
| Fiszdon and Johannesen, 2010 [191] | Research study- Psychometric properties of BLERT | III | BLERT | 48 SCZ, 56 HC | Distributions:  HC mean: 17.18 (SD = 1.29)  SCZ mean: 11.39 (SD=3.13)  Relationship to functioning:  r = .49 (p<.01) with UPSA, r=52 (p<.01) with the medication management ability assessment or MMAA, ns correlations with SSPA, QLS, and ILSS |
| Hamm et al., 2012 [192] | Research study- Psychometric properties of BLERT | III | BLERT | 49 SCZ at baseline and 6 months | Six-month test-retest reliability was .54.  No significant change between Time 1 (M=12.57, SD=3.28) and Time 2 (M=12.35, SD=3.81). 36 participants had less than a 33% change, while 13 had a change from baseline to follow-up of 33%-67%. Baseline BLERT correlated .46 with Metacognitive Assessment Scale abbreviated (MAS-A) and .43 at 6-month MAS-A  Correlated .29 (p<.05) with Wisconsin Card Sort. |
| Fiszdon et al., 2013 [193] | Research study- Psychometric properties of BLERT | III | **BLERT**  Hinting task  BORRTI  MSCEIT | 119 SCZ | On three of the four social cognitive tasks (BLERT, BORRTI Egocentricity, MSCEIT-ME) over 50% of participants performed at least 1 S.D. below HC norms. Only 11 of 118 participants (9.32%) scored within (1S.D.) or above HC norms on all four task; 107 (90.68%) scored at least 1 S.D. worse than HC on one or more tasks; 83 (70.34%) scored worse than HC on two or more tasks; 51 (43.22%) scored worse than HC on at least three tasks, and 14 (11.86%) scored worse on all four tasks. Correlations between social cognition and functioning were significant for two social cognitive measures, where in better performance on the BLERT and BORRTI Egocentricity scales were associated with higher ratings on the QLS Common Objects and Activities subscale (r¼0.27, p¼0.003 and r¼0.27, p¼0.003, respectively). |
| **ER-40** | | | | | |
| Kohler et al., 2003 [194] | Research study- Psychometric properties of ER-40 | III | ER-40 | 28 SCZ and 61 HC | Distributions:  Mean percentage correct were 63.6% for patients and 71% for control subjects. There was a ceiling effect for happy expressions (97.1% correct for patients, 98.4% for controls). No SDs reported. |
| Silver et al., 2004 [195] | Research study- Psychometric properties of ER-40 | III | ER-40 | 20 SCZ | Distributions:  Mean % correct at pretest was 68.75% (SD= 14.25%) and 76% (SD=10.25%) at post-test.  Utility as a repeated measure: Significant change due to 3 days of brief emotion training (pre-test mean 27.5, SD=5.7; post-test mean 30.4, SD=4.1; t=-2.67, p=.02, d=.60).  Distributions:  Mean % correct at pre-test was 68.75% (SD= 14.25%) and 76% (SD=10.25%) at post-test. |
| Goodman et al., 2005 [196] | Research study- Psychometric properties of ER-40 | III | ER-40 | 35 male forensic SCZ | Distributions:  Mean % correct ranged from 65.7% (SD=12.55%) to 71% (11.55%). |
| Gur et al., 2007 [197] | Research study- Psychometric properties of ER-40 | III | ER-40 | 16 SCZ; 17 HC | Distributions:  Mean percentage correct for patients ranged from 90.66% (SD=10.72%) for happy to 76.90% (SD=13.47%) for fear. Controls ranged from 95.97% (SD=5.21%) for happy to 82.42% (SD=12.11%) for fear. |
| Weiss et al., 2007 [198] | Research study- Psychometric properties of ER-40 | III | ER-40 | 56 SCZ | Distributions:  Mean percentage correct were: happy expressions (92.6%) (95% CI 89.9-94.9%), fear (61.4%) (95% CI 56.7-65.9%), neutral (60.9%) (95% CI 56.3-65.5%), sad (59.8%) (95% CI 55.1- 64.4%), and anger (55.6%) (95% CI  50.8-60.2) |
| Pinkham et al., 2008 [199] | Research study- Psychometric properties of ER-40 | II | ER-40 | 270 SCZ; 270 HC | Distributions:  Mean percentage correct were 75.6% for patients and 82.8% for controls. No SDs reported. |
| Carter et al., 2009 [200] | Research study- Psychometric properties of ER-40 | II | ER-40 | 1023 SCZ; 424 HC | Test-retest reliability is .80 for HC and .76 in SCZ.  Practicality and tolerability: Average testing time are under 5 minutes. Computerized task administered via dedicated hardware or the internet. Automated scoring provides accuracy and median response times. |
| Grant and Beck, 2010 [201] | Research study- Psychometric properties of ER-40 | II | ER-40 | 123 SCZ/SCZaff | Relationship to functioning: Emotion perception (comprised of ER-40 and Penn emotion discrimination test) was significantly correlated with vocational functioning (r=.28, p<.01), but not social functioning (r=-.06), measured with the SFS. |
| Gur et al., 2010 [202] | Research study- Psychometric properties of ER-40 | II | ER-40 | 448 HC (226 females) | Test-retest reliability is .91  Mean percent correct: 84% (SD=7.8%). The ER-40 significantly correlated with immediate face memory (r=.31) and delayed face memory (r=.29).  Significantly correlated with a number of other neurocognitive tests in the Penn CNB at p<.01: PCET (r=.27), CPT L (r=.26), LNB (r=.29), Spatial IMM (r=.34), spatial DEL (r=.31). Significant at p<.001 with PVRT (r=.36) and CJOLO (r=.35). |
| Pinkham et al., 2011 [203] | Research study- Psychometric properties of ER-40 | III | ER-40 | 132 SCZ | Distributions:  Mean % correct ranging from 93% (SE=1.3%) for happy to 63% (SE=2.2% for anger. |
| Irani et al., 2012 [204] | Research study- Psychometric properties of ER-40 | III | ER-40 | 624 SCZ; 624 HC | Distributions:  Z-scores reported only: -.25 young HC (under age 45,) -.1 older HC (over 45), -1.0 and greater for patients across ages |
| **MSCEIT** | | | | | |
| Mayer et al., 2003 [171] | Instrument validation and Psychometric properties of MATRICS | II | MATRICS: MSCEIT | 2112 HC | Comparison of test-booklet versus on-line administration groups:  Comparison of booklet and on-line tests. For each, there were 705 responses to the test (141 items; × five responses each). The correlation between response frequencies for each alternative across the two methods was r (705) = .987.  Reliability:  The MSCEIT full-test split-half reliability is r(1985) =  .93 for general and .91 for expert consensus scoring. The four branch scores of Perceiving, Facilitating, Understanding, and Managing range between  r(2004–2028) = .76–.91 for both types of reliabilities.  Correlational and factorial structure:  all tasks were positively intercorrelated using both general (reported below the diagonal) and expert consensus scoring (above the diagonal). The intercorrelations among tasks ranged from r (1995–2111) = .17 to .59, ps < .01, but with many correlations in the mid .30s.  There was a progressively better fit of models from  the one- to the four-factor model, but all fit fairly well  (four vs. two factors, *x2* (4) = 253, p < .001; two vs. one factors, *x2* (1) = 279, p < .001). |
| Extremera et al., 2006 [205] | Research study-  Psychometric properties and Co-normalization of MATRICS in Spain | III | MATRICS: MSCEIT | 946 HC (426 males, 520 females) | The results here are consistent with and support recent findings with the English version of the MSCEIT.  These analyses revealed good reliability and internal consistency for the Spanish version of the MSCEIT; a high level of convergence between scoring methods; and higher scores obtained by women on overall scale and branches scores than scores obtained by men. A positive correlation between the scores on MSCEIT and age was found. |
| Nuechterlein et al., 2008 [107] | Instrument validation and Psychometric properties of MATRICS | II | MATRICS: MSCEIT: managing emotions branch; perceiving emotions branch | 176 SCZ and readministered to 167 of them 4 weeks later so that the 20 tests could be compared directly. | Test-retest reliability:  ICC coefficient was .80 and .73 respectively for managing emotions branch and perceiving emotions branch.  Utility as a repeated measure:  *Managing emotions branch*: T1: 85.6 (11.0); T2: 85.0 (10.6); T1-T2 difference: -.6 (8.0); t= .95; p= .34; effect size=.06; *perceiving emotions branch*: T1: 94.7 (16.6); T2: 93.9 (18.3); T1-T2 difference: -.8 (11.0); t= .97; p= .33; effect size=.04; Relationship to functional outcome:  *Managing emotions branch*: Global: .40; Work functioning: .17; Social functioning: .11; Independent living: .20; *perceiving emotions branch*: Global: .00 Work functioning: .05 Social functioning: -.12; Independent living: .10. Practicability:  *managing emotions branch*: 4.9 (1.8);  *perceiving emotions branch*: 5.4 (2.0) Tolerability:  *Managing emotions branch*: Participant’s ratings: 5.2 (1.2); administration time:  12.0 (3.1);  *Perceiving emotions branch*:  Participant’s ratings: 4.9 (1.2); administration time: 7.4 (3.1) |
| Kee et al., 2009 [206] | Research study-  Psychometric properties of MSCEIT | III | MSCEIT branches: 1 Emotion perception;  2 Emotion facilitation;  3 Emotion understanding;  4 Emotion management. | 50 SCZ and 39 HC | The internal consistency:  Cronbach's alphas revealed generally high reliability coefficients for most MSCEIT branches (Identifying Emotions, α = 0.86; Using Emotions, α = 0.83; Understanding Emotions, α = 0.82; and Managing Emotions, α = 0.72) and the total score (α = 0.93) in the schizophrenia sample. Although reliability estimates for control subjects were somewhat lower, they were within adequate range (Identifying Emotions, α = 0.89; Using Emotions, α = 0.78; Understanding Emotions, α = 0.68; and Managing Emotions, α = 0.67), with the total score showing the highest level (α = 0.90). Also, the test for the distribution of the MSCEIT total score (skewness = − 0.10, standard error = 0.34) in the patient group was fairly comparable to that of the control group score (skewness = − 0.40, standard error = 0.39), indicating relatively normal distributions for both groups.  Relationship to functioning:  There were several statistically significant associations: Identifying Emotions with work productivity; all of the components of the MSCEIT with independent living/self-care; Identifying Emotions with relationships with family and spouse; and Identifying Emotions and Understanding Emotions with psychosocial adjustment. The magnitudes for the above correlations were small to medium, and in these correlations lower levels of emotional intelligence processes were associated with poorer psychosocial outcome in schizophrenia. |
| Eack et al., 2010 [207] | Research study-  Psychometric properties of MSCEIT | III | MSCEIT branches: 1 Emotion perception;  2 Emotion facilitation;  3 Emotion understanding;  4 Emotion management. | 64 SCZ/SCZaff | Reliability:  all the MSCEIT branches displayed adequate levels of internal consistency, with the emotion perception branch displaying the highest level of internal consistency and the emotion understanding branch displaying the lowest but still acceptable level of internal consistency. Reliability estimates were somewhat lower for individual tasks, with the blends and emotion management tasks displaying suboptimal levels of internal consistency.  Convergent Validity:  MSCEIT demonstrated little convergence with the Social Cognition Profile. No areas of MSCEIT performance were significantly related to any of the Social Cognition Profile subscales. Discriminant Validity:  all MSCEIT scale scores were significantly positively related to neurocognitive composite scores, although these relationships were at most moderate in size, suggesting that while related the MSCEIT was sufficiently independent of measures of neurocognitive function. Relationship to functioning:  of the 7 measures of functional outcome provided by the Social Adjustment Scale-II, MSCEIT performance was related to 2: major role functioning and interpersonal anguish. Only overall emotional intelligence demonstrated a statistically significant relationship with better role functioning; however, trends were also observed between role functioning and emotion understanding. In addition, nonsignificant trends were observed pointing to a relationship between better emotion facilitation abilities, less interpersonal anguish, and better role functioning. Social functioning was significantly related to overall emotional intelligence and emotion perception ability, and trends were observed between better social functioning and emotion understanding and management abilities. In addition, global work potential was also related to overall emotional intelligence and emotion facilitation and management abilities. |
| Karim and Weisz, 2011 [208] | Research study-  Psychometric properties of MSCEIT in France and Pakistan | III | MSCEIT | 192 students from two non-native English  speaking national cultures: 111 from France  (49 males, 62 females), and 81 from Pakistan (52 males and 29 females). | French participants had higher scores than their Pakistani counterparts on branch, area, and total MSCEIT scores. This accords well with findings showing that people from individualistic societies are better at perceiving, understanding, expressing, and regulating emotions. The MSCEIT has the same theoretical latent structure, the same strength of the relationships among factors and tasks, and the same reliability of tasks regardless of the country. Therefore, the MSCEIT across both cultures can be interpreted in the same way. Furthermore, in both cultures, the MSCEIT scores failed to demonstrate incremental validity against well-being measures, after controlling for cognitive intelligence and the Big Five personality dimensions. Finally, within each sample, females significantly scored higher than males on the MSCEIT total scores. |
| Curci et al., 2013 [209] | Research study-  Psychometric properties of the Italian version of MSCEIT | III | MSCEIT | 183 HC | Two studies were conducted with the aim of providing a contribution in the evaluation of the validity of the Italian version of the MSCEIT version 2.0. The MSCEIT was administered in two batteries, the first including cognitive, personality, and affect measures (Study 1), the second made up of indexes of psychological well-being (Study 2). The results confirm that the MSCEIT Italian version satisfactorily discriminates emotional intelligence ability from crystallized and fluid intelligence, personality, and affect, and exhibits significant correlations with various psychological well-being criteria. Furthermore, data from both studies confirm that the factorial structure of MSCEIT is consistent with the theory on which it is based, although it was difficult to rule out alternative structures. |
| Fiszdon et al., 2013 [193] | Research study-  Psychometric properties of BLERT | II | **BLERT**  Hinting task  BORRTI  MSCEIT | 119 SCZ | On three of the four social cognitive tasks (BLERT, BORRTI Egocentricity, MSCEIT-ME) over 50% of participants performed at least 1 S.D. below HC norms. Only 11 of 118 participants (9.32%) scored within (1S.D.) or above HC norms on all four task; 107 (90.68%) scored at least 1S.D. worse than HC on one or more tasks; 83 (70.34%) scored worse than HC on two or more tasks; 51 (43.22%) scored worse than HC on at least three tasks, and 14 (11.86%) scored worse on all four tasks. The MSCEIT-ME correlated positively with QLS Common Objects and Activities and GAF ratings, and negatively with QLS Instrumental Role Function, but these associations were observed only at trend-level. |
| Lindenmayer et al., 2013 [210] | Research study-  Psychometric properties of MSCEIT | III | MSCEIT | 59 stable schizophrenia or schizoaffective predominantly inpatients were randomized to either CRT (N = 27) alone or CR + MRIGE (N = 32) for 12 weeks. | A significant group by time effect for social cognition, measured by the MSCEIT was found (F = 5.473, P = .050): CRT + MRIGE demonstrated significantly greater improvement than CRT alone (CRT + MRIGE, Z = 1.98, P = .02; CR alone, Z = 1.00, P = .05). |
| Mao et al., 2016 [211] | Research study-  Psychometric properties of the Chinese version of MSCEIT (MSCEIT-TC) | III | MSCEIT-TC (Traditional Chinese)  Four branches: 1. Perceiving;  2. Facilitating;  3. Understanding  4. Managing; | 76 SCZ and 728 HC | The results suggest that the MSCEIT-TC is reliable and valid when assessing emotional intelligence. The results showed good discrimination and validity when comparing the two study groups. Impairment was the greatest for two branches Understanding and Managing Emotions, which implies that the deficits of schizophrenia individuals involve ToM tasks. Deficits involving the negative scale of schizophrenia was related to impaired performance when the MSCEIT-TC was used (in branch 2, 3, 4, and the area Strategic). |
| McClery et al., 2016 [212] | Research study-  Psychometric properties of MSCEIT and RAD | III | **MSCEIT**  RAD | 41 SCZ | Performance on the two social cognition tasks were stable over follow-up. There were no significant mean differences between assessment points [p's ≥ 0.20, Cohen'sd' s≤\|0.20\|], and baseline performance was highly correlated with performance at follow-up [ρ's ≥ 0.70, ICC ≥ 0.83, p's b 0.001].  Relationship to functioning:  The contemporaneous association between social cognition and community functioning was moderately large at follow-up [ρ = 0.49,  p = 0.002]. However, baseline social cognition did not show a significant longitudinal influence on follow-up community functioning [z=0.31, p=0.76]. |
| DeTore et al., 2018 [213] | Psychometric properties of MSCEIT-ME | II | MSCEIT-ME | 107 SCZ | Discriminant validity:  Performance on the MSCEIT-ME was significantly correlated with processing speed, attention/working memory, learning within schizophrenia-schizoaffective disorder, bipolar disorder, and other mixed diagnoses groups. Relationship to functioning:  Better performance on MSCEIT-ME was associated with better psychosocial functioning on the Quality-of-Life Scale (QLS) in the schizophrenia-schizoaffective disorder group, but not in the bipolar or other mixed diagnoses groups. |
| **Theory of mind** | | | | | |
| **Hinting task** | | | | | |
| Corcoran, Mercer, and Frith, 1995 [153] | Research study-  Validation and Psychometric properties of Hinting task | III | Hinting task | 55 SCZ and 30 HC | Distributions: SCZ: 78% correct (SD=19.5%); HC: 91.5% correct (SD=8%). Discriminant validity: SCZ: IQ (r=.49*); HC: IQ (r=.08, ns). |
| Corcoran., 2003 [214] | Research study-  Psychometric properties of Hinting task | III | Hinting task | 39 SCZ 44 HC | Distributions:  SCZ = 82.5% (20%); HC = 92.5% (6%).  Discriminant validity:  SCZ: Means-ends problem solving (r=.38*); IQ (.29, ns); HC: Hinting uncorrelated with IQ and means ends social problem solving. |
| Corcoran and Frith, 2003 [215] | Research study-  Psychometric properties of Hinting task | III | Hinting task | 59 SCZ 44 HC | Distributions:  SCZ = 74.3% (26.6%); HC = 94.5% (5.1%). Discriminant validity:  Hinting task and ToM stories (.63, p < .05). |
| Janssen et al., 2003 [154] | Research study-  Psychometric properties of Hinting task | III | Hinting task | 43 SCZ/SCZaff 41 first degree relatives 43 HC | Convergent validity:  Hinting task was associated with the false belief task (OR = 1.43, 95% CI 0.45–4.56). |
| Greig et al., 2004 [216] | Research study-  Psychometric properties of Hinting task | II | Hinting task | 128 SCZ/SCZaff | Distributions:  SCZ/SCZaff = 80.4% (19.15%).  Discriminant validity:  Pearson correlations: Verbal memory (.42*); Figure memory (.28*), executive functioning (.34*), Global IQ (.31*). |
| Marjoram et al., 2005 [217] | Research study-  Psychometric properties of Hinting task | III | Hinting task | 15 SCZ 15 affective disorder 15 HC | SCZ performed less well than either the controls or patients with affective. Distributions:  The mean was 15.5 (2.2) in SCZ; 18.2 (1.7) in Affective disorder and 19.2 (1.1) in HC. |
| Penn et al., 2005 [218] | Research study-  Psychometric properties of Hinting task | III | Hinting task | 7 SCZ treated with SCIT | Utility as a repeated measure:  Baseline = 12.1 (4.8) Post-test = 15.3 (3.4) |
| Silverstein et al., 2005 [219] | Research study-  Psychometric properties of Hinting task | III | Hinting task | 26 SCZ | Relationship to functioning: Micro-module learning test (MMLT) (r=.77*). |
| Schenkel et al., 2005 [220] | Research study-  Psychometric properties of Hinting task | III | Hinting task | 42 SCZ/SCZaff | Discriminant validity:  Pearson correlations: Goodness of fit context task (.47*); Contour task (.43*). Executive functioning, verbal fluency, IQ (ns). Relationship to functioning:  Poorer performance on the Hinting Task was associated with poor premorbid social functioning rated from medical charts (social history interview) (t (40) = 3.86, p < 0.0001). |
| Bora et al., 2006 [221] | Research study-  Psychometric properties of Hinting task | III | Hinting task  Eyes task | 50 SCZ | **Hinting task**  Convergent validity:  Pearson correlations: Eyes (.51*) (note: correlation attenuated when accounting for neurocognitive index). Discriminant validity:  Auditory consonant trigrams (.61*), IQ (.54*); trails B (-.44*). Relationship to functioning:  Social functioning Scale total (r=.43*); after controlling for cognitive functioning: (r=.24, ns). |
| Uhlhaas et al., 2006 [222] | Research study-  Psychometric properties of Hinting task | III | Hinting task  Eyes-task | 48 SCZ/SCZaff 26 HC | **Hinting task**  Discriminant validity:  SCZ/SCZaff: (r = -.33*); HC: visual size performance task (r = -.35*). |
| Bertrand et al., 2007 [223] | Research study-  Psychometric properties of Hinting task | III | Hinting task | 36 SCZ and 25 HC | Distributions:  SCZ: 76.55% (15.55%); HC: 90.35% (7.35%). Discriminant validity:  SCZ: IQ (r = .46*); HC: IQ (r = .18, ns). |
| Bora et al., 2007 [224] | Research study-  Psychometric properties of Eyes-task | III | Hinting task  Eyes-task | 58 SCZ | Distributions:  The mean hinting task score was 18.16 (4.68) (out of 36) in SCZ.  Discriminant validity:  Pearson correlations First order ToM (.24, ns); Second order ToM (.40*) Digit span forward (.41*), Digit span backward (.44*), letter to number (.44*); WCST and verbal fluency (ns). |
| Mizrahi et al., 2007 [225] | Research study-  Psychometric properties of Hinting task | III | Hinting task | 71 SCZ | Distributions:  The mean hinting task score was 16.82 (S.D.=2.8). Utility as a repeated measure:  Scores on the hinting task were significantly different from baseline at 2 weeks (t=–2.30, df=17, p=0.034), and continued to improve thereafter. |
| Bora et al., 2008 [226] | Research study-  Psychometric properties of Hinting task | III | Hinting task | 91 SCZ 55 HC | Distributions:  SCZ = 74% (22.5%); HC = 80% (17%). Discriminant validity:  Hinting was significantly correlated with neurocognition (r=.516, p<.01). |
| van Hooren et al., 2008 [227] | Research study-  Psychometric properties of Hinting task | III | Hinting task | 44 SCZ 47 familial risk 41 psychometric risk 54 HC | Convergent validity:  Hinting uncorrelated with speech attribution task, beads task and internal, personal, situational attributions task (IPSAQ). Discriminant validity:  Hinting significantly correlated with the following neurocognitive measures: SCWT-speed (.35), SCWT (.31), TMT-speed (.33), TMT (.30), semantic fluency (.44) (all significant to p<.01). |
| Bell et al., 2009 [189] | Research study-  Psychometric properties of Hinting task | II | Hinting task | 151 SCZ/SCZaff | Distributions:  The mean hinting task score was 16.02 (S.D.=3.68). Convergent validity: BLERT total (r = .17*); Discriminant validity:  Proverbs total (r = .45*); Verbal learning (r=.25*), Wisconsin card sorting test (WCST) (.26*), digit span (r=.23*).  Relationship to functioning: Work behaviour inventory (r = .355*). |
| Roberts and Penn, 2009 [190] | Research study-  Psychometric properties of Hinting task | III | Hinting task | 31 SCZ | Reliability:  Test-retest reliability is .65. In the TAU group, hinting task demonstrated good test-retest reliability (effect size of d=-.06, pre-test mean 15.45 (SD=2.94), post-test mean 15.27 (SD=3.38) (over 6 months).  Utility as a repeated measure:  SCIT: Pre-test = 16.14 (2.66) Post-test = 15.92 (2.59)  TAU: Pre-test = 15.45 (2.94) Post- test = 15.27 (3.38).  No significant treatment effect. |
| Bell et al., 2011 [228] | Research study-  Psychometric properties of Hinting task | III | Hinting task | 77 SCZ/SCZaff | Distributions:  All subjects had a mean of 16.7 (2.2) in hinting task score; high negative symptoms 15.7 (2.4); higher social cognition 17.3 (2.0); lower social cognition 16.9 (2.0).  Convergent validity:  MSCEIT managing emotions branch (r=.22*); social attribution task (r=.14, ns). |
| Couture et al., 2011 [229] | Research study-  Psychometric properties of Hinting task | II | Hinting task | 178 SCZ/SCZaff | Distributions:  The mean of Hinting task score was 13.2 (4.5) in SCZ/SCZaff.  Discriminant validity:  Hinting was significantly correlated with neurocognition (r=.516, p<.01).  Relationship to functioning:  Social competence (role play) (r=.41*); self-reported functioning (r=.17*). |
| Liu et al., 2011 [230] | Research study-  Psychometric properties of Hinting task | II | Hinting task | 180 SCZ | Test-retest reliability is .59. Utility as a Repeated Measure:  Baseline: 14.6 (3.3); 6 months: 15.19 (3.3); 12 months: 15.44 (4.1). Distributions:  Ranges from 73% (16.5%) at intake to 77.2% (20.5%) 12 months later. Relationship to functioning:  Hinting task not correlated with social functioning on the unit as measured by the NOSIE. |
| Lysaker et al., 2011 [231] | Research study-  Psychometric properties of Hinting task | III | Hinting task | 36 SCZ/SCZaff at baseline and after 6 months | M=11.89 (4.80) baseline; M=12.89 (4.40) retest. Utility as a Repeated Measure:  Baseline = 11.89 (4.80); 6 months = 12.89 (4.40). Distributions:  Mean 11.17 (4.80).  Convergent validity:  Hinting and Bell Lysaker Emotion Recognition Test (BLERT) (r=.52*) Hinting and Eyes test (r=.47*). |
| Menon, et al., 2013 [232] | Research study-  Psychometric properties of Hinting task | III | Hinting task | 18 SCZ or SCZaff 17 HC | Distributions:  SCZ/SCZaff = 16.67 (3.6); HC = 19.18 (1.1).  Convergent validity:  Hinting and social variant of probabilistic reasoning (-.35*). Hinting uncorrelated with IPSAQ. |
| Rubio et al., 2011 [233] | Research study-  Psychometric properties of Hinting task | III | Hinting task | 42 SSI 21 siblings 77 HC | Convergent validity:  Hinting and Draws to Decisions (jumping to conclusions task) (r=-.389*). |
| Choi et al., 2012 [234] | Research study-  Psychometric properties of Hinting task | III | Hinting task | 36 SCZ | Convergent validity:  Hinting uncorrelated with emotion context processing scale. |
| Fanning et al., 2012 [235] | Research study-  Psychometric properties of Hinting task | II | Hinting task | 119 SCZ/SCZaff | Discriminant validity:  MATRICS battery correlations: processing speed (r=.39, p<.001), working memory (r=.24, p<.01), verbal learning (r=.23, p<.05), and composite neurocognition (r=.28, p<.01). |
| Gil et al., 2012 [155] | Research study-  Validation and Psychometric properties of the Spanish version of the Hinting task | III | Hinting task | 40 SCZ and 39 HC | Reliability:  Good reliability data was obtained in the inter-observer and test---retest in the two samples. On the other hand, the internal consistency was somewhat low for all of the 10 histories. For this reason, and starting from a previous study, a reduced version of 5 histories was prepared, which showed good internal consistency. SCZ obtained a significantly lower score than HC in 8 out of the 10 histories. The reduced Spanish version of the Hinting Task demonstrated good psychometric properties. When compared to HC, SCZ had a deficit in theory of mind. |
| Meijer et al., 2012 [236] | Research study-  Psychometric properties of Hinting task | II | Hinting task Trustworthiness task | 1093 SCZ 1044 unaffected siblings 911 Parents 587 HC | Patients performed significantly worse than controls in Hinting task (< 0.001).  Distributions:  SCZ = 17.54 (2.78); Siblings = 18.84 (1.66); Parents = 18.79 (1.62); HC = 19.08 (1.31). |
| Tas et al., 2012 [237] | Research study-  Psychometric properties of Hinting task | III | Hinting task  Eyes task  FEIT  FEDT  IPSA-Q  UOT | 52 SCZ | Utility as repeated measure:  SCIT: Pre-test: 13.16 (2.41); Post-test: 15.63 (1.80);  Social stimulation: Pre-test: 12.96 (4.28); Post-test: 11.92 (4.28). |
| Fiszdon et al., 2013 [193] | Research study-  Psychometric properties of Hinting task | II | Hinting task  BLERT  BORRTI  MSCEIT | 119 SCZ | On three of the four social cognitive tasks (BLERT, BORRTI Egocentricity, MSCEIT-ME) over 50% of participants performed at least 1 S.D. below HC norms. On the Hinting Task, the majority of participants scored within 1 S.D. of HC norms. Only 11 of 118 participants (9.32%) scored within (1 S.D.) or above HC norms on all four task; 107 (90.68%) scored at least 1S.D. worse than HC on one or more tasks; 83 (70.34%) scored worse than HC on two or more tasks; 51 (43.22%) scored worse than HC on at least three tasks, and 14 (11.86%) scored worse on all four tasks. |
| Frøyhaug et al., 2019 [157] | Research study- Validation and Psychometric properties of Hinting task in Norway | III | Hinting task Trustworthiness task | 30 SCZ/SCZaff, 31 BD-I, 29 BD-II and 183 HC | The Hinting Task displayed adequate levels of internal consistency for schizophrenia and bipolar I disorder.  Ceiling effects emerged in all groups except the schizophrenia group. Schizophrenia patients scored significantly lower than all other groups, but no other significant group differences were detected. In the schizophrenia group, the Hinting Task’s concurrent validity was substantiated by significant correlations with measures of neurocognition, symptoms and functional capacity. In the bipolar disorder groups, however, only a few significant relationships were found. Correlations between the Hinting Task and a measure of emotion recognition indicated that construct validity was higher for schizophrenia than bipolar disorder. The results suggest that the Norwegian Hinting Task is suited for use in schizophrenia research and assessment, but caution is warranted when using the test for other populations. |
| Krawczyk et al., 2020 [158] | Research study-Validation and  Psychometric properties of the Polish version of Hinting task | III | Hinting task Trustworthiness task | 50 SCZ and 50 HC | Results indicated satisfactory psychometric properties (internal consistency, inter-rater reliability and external validity) sufficient for between group comparisons for research purposes. The presented test highly distinguished two assessed groups: individuals with schizophrenia and healthy controls. Contrary to the expectations, in the clinical group scores in the Hinting Task did not correlate with the severity of psychopathological symptoms. Polish translation of the Hinting Task has psychometric properties appropriate for the research setting. Usefulness of the Hinting Task in other clinical groups should be addressed in future research. |
| **Eyes Task** | | | | | |
| Russell et al.; 2000 [238] | Research study-Psychometric properties of Eyes task | III | Eyes task | 5 SCZ 7 HC | Distributions:  SCZ = 12.6 (5.03); HC = 6.14 (3.84). |
| Baron-Cohen et al., 2001 [165] | Research study-  Psychometric properties of Eyes task | III | Eyes task | 103 SCZ; 15 AS and 122 HC | Distributions:  SCZ = 28.0 (3.5); AS = 21.9 (6.6); HC = 26.2 (3.6). Discriminant validity:  Eyes and autism quotient (r = -.53, p = .004); Eyes and IQ (r = .09, ns). |
| Craig et al., 2004 [239] | Research study-Psychometric properties of Eyes task | III | Eyes task | 16 SCZ, 17 AS and 16 HC | Distributions:  SCZ: 18.19 (6.65); Aspergers: 19.88 (6.10); HC: 27.63 (4.33); Convergent validity:  Eyes and Hinting task (r = .54, p < .01). |
| Kelemen et al., 2005 [240] | Research study-Psychometric properties of Eyes task | III | Eyes task | 52 SCZ (split into remitted and non-remitted) 30 HC | Distributions:  Mean of Eyes task score in: SCZ (remitted) = 19.6 (4.5); SCZ (non-remitted) = 17.9 (5.4); HC = 22.5 (2.9). Discriminant validity:  Eyes uncorrelated with IQ. |
| Bora et al., 2007 [224] | Research study-Psychometric properties of Eyes test | III | **Eyes test**  Hinting task | 50 SCZ | **Eyes task**  Distributions:  Mean of eyes task in SCZ (good outcome) = 16.2(0.6); Mean of eyes task in SCZ (poor outcome) = 12.7(0.6). Convergent validity:  Pearson correlations (* < .05): -Hinting task (.51*). Discriminant validity:  Pearson correlations (* < .05): -Auditory Consonant Trigrams (.47*); -WAIS information (.25) (ns). Relationship to functioning:  Beta = .34, p = .03 with Social Functioning Scale (SFS) total; beta = .36 p = .01 for SFS interpersonal, beta = .40 (p = .001) for SFS social activities, and beta = .30 p = .05. Practicability and tolerability: 36 items, multiple choice, requires no informant rating. |
| Uhlhaas et al., 2006 [222] | Research study-Psychometric properties of Eyes-task | III | Eyes-task  Hinting task | 48 SCZ/SCZaff 26 HC | **Eyes task**  Discriminant validity:  Eyes and visual size perception task (r=.10, ns). |
| Couture et al., 2008 [241] | Research study-Psychometric properties of Eyes task | III | Eyes task Trustworthiness task | 26 young SCZ 88 CHR 41 HC | **Eyes task**  Distributions:  SCZ = 62.7% (13.7);  CHR = 69.6% (13.8);  HC = 68.8% (14.0). Convergent validity:  ns with Trustworthiness task. |
| McGlade et al., 2008 [242] | Research study-Psychometric properties of Eyes task | III | Eyes task | 73 SCZ/SCZaff 78 HC | Convergent validity:  Eyes task (r=.38*). Discriminant validity:  Working memory (p < .05); IQ and spatial memory (ns). Relationship to functioning:  Independent Living Scale (ILS) was not significantly associated with Hinting task performance. |
| Hallerback et al., 2009 [243] | Research study-Psychometric properties of Eyes task | II | Eyes task | 158 SCZ | Reliability:  Pearson ‘s r between initial and follow up testing (3 weeks) = .60, p < .01. Distributions:  Scores were not normally distributed. For all participants (out of 28); Including 4 questionable items = 20.5 (2.4); W/o questionable items (out of 24) = 18.9 (2.1); 58 participants who took it twice: 1st test (with questionable items)- 20.7 (2.5), 2nd test- 19.2 (2.2); 1st test (with questionable items- 20.9 (2.8), 2nd test- 18.9 (2.6). |
| Tas et al., 2012 [237] | Research study-Psychometric properties of Eyes task | III | **Eyes task**  Hinting task  FEIT  FEDT | 52 SCZ | Utility as repeated measure:  **Eyes task**  SCIT: Pre-test: 20.26 (4.12); Post-test: 20.16 (4.32)  Social stimulation: Pre-test: 18.27 (4.85); Post-test: 17.45 (5.40). |
| Vellante et al., 2013 [166] | Research study-Co-normalization of Eyes task in Italy | III | Eyes task | 200 HC | Reliability:  Internal consistency (Cronbach’s alpha) was .605. Confirmatory factor analysis provided evidence for a unidimensional model, with maximal weighted internal consistency reliability =.719. Test–retest reliability for the Eyes test, as measured by intraclass correlation coefficient, was .833 (95% confidence interval = .745 to .902). Females scored significantly higher than males on both the Eyes test and the empathy quotient, replicating earlier work. Those participants who scored lower than 30 on the empathy quotient (n = 10) also scored lower on the Eyes test than those who did not (p <.05). Eyes test scores were not related to social desirability. This study confirms the validity of the Eyes test. Both internal consistency and test–retest stability was good for the Italian version of the Eyes test. |
| Prevost et al., 2014 [169] | Research study-Co-normalization of Eyes task in France | III | Eyes task | 109 HC for English version and 97 HC for French version | Distributions:  Similar overall distribution of scores in both versions and no differences in the mean scores between them. However, 2 items in the French version did not collect a majority of responses, which differed from the results of the English version. Test-retest showed good stability of the French version. As expected, participants who do not speak French or English at home, and those born in Asia, performed worse than North American participants, and those who speak English or French at home. French version had acceptable validity and good stability. The cultural differences observed support the idea that Asian culture does not use theory of mind to explain people’s behaviours as much as North American people do. |
| Đorđević et al., 2017 [168] | Research study-Co-normalization of Eyes task in Serbia | III | Eyes task | The sample consisted of  both unimpaired controls (76.5%), and a clinical group of participants (SCZ 49,8 % and BD 50,8%) | Eyes task has demonstrated fair psychometric properties (KMO = .723; α = .747; H1 = .076; H5 = .465), successfully differentiating between clinical group and control [F (1,254) = 26.175, p <.001, η2 p = .093], while typical gender differences in performance were found only in control group. Tests of several models based on the previous literature revealed that the affect-specific factors underlying performance on Eyes task demonstrate poor fit. The best fitting model obtained included reduced scale with a single-factor underlying the test’s performance (TLI = .953, CFI = .958, RMSEA = 020). Based on the fit parameters we propose 18-item short-form of the Serbian version of Eyes task (KMO = .797; α = .728; H1 = .129; H5 = .677) for economic, reliable and valid measurement of ToM abilities. |
| Lee et al., 2020 [167] | Research study-Co-normalization of the Eyes Task in Korea | III | Eyes task | 200 HC | The Korean version of the Eyes task also showed good test-retest reliability over a 4-week time interval. Convergent validity was also supported by significant correlations with subscales of the TAS-20K, and discriminant validity was identified by non-significant associations with IRI-C scores. In addition, no difference was found in Eyes task performance according to the sex of the photographed individuals or the sex or educational attainment of the participants. |
| Chakrabarty et al., 2021 [170] | Research study-Psychometric properties of the Indian version of the Eyes Task | III | Eyes task | 23 SCZ, 22 BD and 104 HC | Reliability:  Moderate internal consistency (Cronbach’s alpha = 0.6) and test-retest reliability (ICC’s = 0.64, P < 0.001). Positive correlations were found between Eyes task and Wechsler picture arrangement (r = 0.60, P < 0.001), picture completion (r = 0.54, P < 0.001), and comprehension subtests (r = 0.48, P < 0.001). Patients with SCZ (M = 49.7, standard deviation [SD] = 16.5) scored significantly lower than HC (M = 68.9, SD = 13.8) (P = 0.008; Cohen’s d = 1.3) on Eyes task. Thus, this tool could discriminate patients who are reported to have Theory of Mind deficits from healthy controls. The Bengali version of the Eyes task is a reliable and valid tool for assessing first‑order ToM insofar as the original Eyes task measures this construct. |
| **TASIT** | | | | | |
| Roberts et al., 2009 [190] | Research study-Psychometric properties of BLERT, TASIT and AIHQ | III | BLERT  TASIT  AIHQ | 31 SCZ | **TASIT**  Utility as a repeated measure:  Social cognition and interaction training:  Baseline: 26.30 (6.90); Post-test: 29.50 (5.72); TAU: Baseline: 27.38 (5.42); Post-test: 27.50 (5.73). |
| Sparks et al., 2010 [244] | Research study-Psychometric properties of TASIT | III | TASIT | 30 SCZ/SCZaff 25 HC | Relationship to functioning: TASIT part 1 total: Life satisfaction (ß = −.43*); TASIT part 3 sarcasm: Life recreational engagement ((ß = −.53*). |
| Wynn et al., 2010 [245] | Research study-Psychometric properties of TASIT | III | TASIT  PONS | 33 SCZ 42 HC | **TASIT**  Distributions:  Part III only (out of 64) The mean TASIT score was 47.2 (8.2) in SCZ and 50.5 (6.2) in HC.  Distributions:  A between-group t test  on the PONS revealed a marginal group difference, t (68) 1.83,  p .08, with schizophrenia patients showing a mean (SD) score  of 80.1 (5.0) compared with the healthy control subjects’ score of  82.5 (5.7), for an effect size of .45. |
| Galderisi et al., 2014 [62] | Research study | II | 921 SCZ | TASIT  MSCEIT  FEIT  Structural equation model | Social cognition, whose assessment included the TASIT, as well as MSCEIT and FEIT, was correlated with the real-life functioning and functional capacity at baseline. |
| Rocca et al., 2016 [173] | Research study | II | 809 SCZ | TASIT  MSCEIT  FEIT  Cluster analytic approach | Social cognition, whose assessment included the TASIT, as well as MSCEIT and FEIT, was correlated with the real-life functioning and functional capacity at baseline. |
| Galderisi et al., 2018 [67] | Research study | II | 921 SCZ | TASIT  MSCEIT  FEIT  Network analysis | TASIT-1 was connected to all the other social cognition nodes and bridged the social cognition domain with the functional capacity node and, through the functional capacity node, with the real-life functioning nodes. |
| **Social perception** | | | | | |
| **RAD** | | | | | |
| Sergi et al., 2009 [178] | Research study-Psychometric properties of RAD | III | RAD | 48 SCZ; 34 HC; 140 undergraduates | Reliability:  SCZ, α=.85; HC, α=86; Undergrads, α= .68. Distribution:  Mean percent’s correct were 68.3% (SD = 9.3%) for SCZ, 77.6 % (SD = 7.3%) for HC, and 74.7% (SD = 7.7%) for undergrads. Relationship to functioning: For SCZ, r=.34 with independent living/self-care, r=.36 with relationships with family and spouse, and r=.31 with relationships with friends (all p<.05) r=.12 with work productivity (p= 0.44). Practicability and tolerability:  Administration typically required about 35 min for participants in all groups. Required reading level is estimated at 9th grade. |
| Green et al., 2012 [58] | Research study- Psychometric properties of RAD | III | RAD | 50 prodromal SCZ matched to 34 controls; 81 first-episode SCZ matched to 46 controls; 53 chronic SCZ matched to 47 controls | Distributions:  Prodromal mean: 55.49 (SD=7.85); control mean: 58.70 (SD=4.84); First-episode correct: 48.61 (SD=9.11); control mean: 56.88 (SD=5.72); Chronic correct: 51.40 (SD=6.89); control mean: 57.09 (SD=8.00); In percent correct: Prodromal mean = 74%, control mean = 78%; First-episode correct: 65%, control mean 76%; Chronic mean: 69%, control mean 76%.  Convergent validity:  In combined clinical samples, r=.621 with MSCEIT total, r=.635 with TASIT total In combined control samples, r=.322 with MSCEIT total, r=.520 with TASIT total; All correlations significant at p<.001. |
| Horan et al., 2012 [59] | Research study-Psychometric properties of RAD | III | RAD  TASIT  MSCEIT | 55 first episode SCZ | Distributions:  12-month test-retest reliability was .74. Utility as a repeated measure: Time 1 mean: 48.67 (SD=8.7); Time 2 mean: 51.09 (SD=9.64) d = .35 for time 1 vs. time 2.  Relationship to functioning: A baseline composite score of RAD, TASIT, and MSCEIT correlated .51 with 12-month work functioning, .38 with 12-month independent living, .34 with 12-month social functioning. The 12-month follow-up composite score correlated .59 with 12-month work functioning, .44 with 12-month independent living, and .48 with 12-month social functioning. All correlations significant at p<.01. |
| McClery et al., 2016 [212] | Psychometric properties of MSCEIT and RAD | III | MSCEIT  RAD | 41 SCZ | Performance on the two social cognition tasks were stable over follow-up. There were no significant mean differences between assessment points [p's ≥ 0.20, Cohen'sd' s≤\|0.20\|], and baseline performance was highly correlated with performance at follow-up [ρ's ≥ 0.70, ICC ≥ 0.83, p's b 0.001]. The contemporaneous  association between social cognition and community functioning was moderately large at follow-up [ρ = 0.49,  p = 0.002]. However, baseline social cognition did not show a significant longitudinal influence on follow-up community functioning [z=0.31, p=0.76]. |
| **SAT-MC** | | | | | |
| Bell et al., 2010 [179] | Research study- Psychometric properties of SAT-MC | III | SAT-MC  BLERT  Hinting task  BORRTI  MSCEIT | 66 patients (49 SCZ, 16 SCZaff, 1 Psychosis NOS); 85 HC | Internal consistency:  Cronbach’s Alpha for item to scale consistency was .83. Split-half reliability using Spearman-Brown coefficient was .75.  Convergent validity:  Significant bivariate correlations were found between SAT-MC correct scores for the combined samples with BLERT (r = 0.47, p < 0.001) and with Hinting Task (r = 0.37, p < 0.001). SAT-MC scores were not significantly correlated with the BORRTI subscales Alienation (r = −0.143, p = 0.08), Insecure Attachment (r = −0.05, p = ns) or Social Incompetence (r = −0.07, p = ns). However, they were significantly correlated with Egocentricity (r = −0.37, p < 0.001); that is, better performance on SAT-MC was associated with more pathology on Egocentricity.  For the schizophrenia sample alone, SAT-MC scores had a weaker but similar pattern of correlations to that of the combined samples. They were significantly correlated with BLERT (r = 0.37, p = 0.002), but did not reach significance with Hinting task (r = 0.23, p = 0.07) or with Egocentricity (r = −0.21, p = 0.10). SAT-MC scores were significantly correlated with the Social Cognition index score on the MATRICS (r = 0.29, p = 0.02).  Divergent Validity:  The strongest correlation is with Matrix Reasoning, a non-verbal problem-solving task; there is also a modest relationship to WCST, which also involves non-verbal problem-solving. SAT-MC scores are about equally correlated with Vocabulary and Block Design, which suggests that IQ (not specifically measured here) probably affects performance. Logical Memory I is significantly correlated, but Logical Memory II, which involves delayed recall, is not. SAT-MC scores have modest significant correlations with MATRICS indices of Working Memory and Reasoning and Problem Solving (based on Mazes, also a non-verbal task) and with the Neurocognitive Composite score. SAT-MC was not significantly correlated with any symptom measures (PANSS Positive, r = 0.08; Negative, r = 0.08; Cognitive, r = 0.07; Hostility, r = 0.16; Emotional Discomfort, r = −0.05; SANS, r = 0.09; SAPS, r = −0.07). SAT-MC was also not significantly correlated with reality testing impairments as measured by the BORRTI (Reality Distortion, r = 0.04; Uncertainty of Perception, r = −0.10; Hallucinations and Delusions, r = −0.10). |
| Johannesen et al., 2013 [246] | Research study- Psychometric properties of SAT-MC | III | SAT-MC and SAT-MC-II compared to BLERT | 92 HC (51 performed SAT-MC I and 41 SAT-MC II) | Reliability:  The SAT-MC and SAT-MC-II differed in internal consistency, with alpha = 0.56 and 0.81, respectively. Split-half reliability coefficients were comparable to full-scale coefficients at 0.56 for SAT-MC and 0.83 for SAT-MC-II. For both versions, item analysis indicated that these reliability coefficients would not be substantially improved by removal of items.  Convergent validity:  Consistent with prior results obtained in a clinical sample, both versions of the SAT-MC correlated significantly with BLERT performance in this university sample. The SAT-MC-II yielded a relatively, but not significantly, higher strength of association with the BLERT than the original SAT-MC. BLERT score distributions were essentially identical between the SAT-MC ( = 17.43, SD = 2.24) and SAT-MC-II ( = 17.63, SD = 2.50) samples, with generally high performance in both groups. The correlation between SAT-MC and BLERT remained significant ((92) = .34, p< 0.001), and no change was observed in relationship to other variables of interest  Discriminant validity:  To test discriminant validity with respect to basic cognitive function, correlations between both versions of SAT-MC and Picture Completion were examined. Small, nonsignificant, correlations indicate that SAT-MC and SAT-MC-II performance was not dependent on basic visual attention and problem solving abilities enlisted by this task. Picture Completion performance was also nearly identical between participants completing the SAT-MC ( = 15.22, SD = 2.19) and SAT-MC-II ( = 15.38, SD = 2.28).  Relationship to functioning:  SAT-MC and SAT-MC-II samples did not differ significantly with respect to SAS, SPQ, or BRIA scores. No significant associations were observed between SAT-MC-II performance and SAS or SPQ total self-report ratings |
| Johannesen et al., 2018 [247] | Research study- Psychometric properties of SAT-MC | III | SAT-MC and SAT-MC-II compared to BLERT | 32 SCZ and 30 SUD | In SCZ SAT-MC and SAT-MC-II showed similar patterns of association with SCOPE tests, with medium correlations with BLERT and TASIT and no appreciable relationship to the Hinting Task or AIHQ.  Internal consistency:  Alpha values reflected good inter-item consistency (alpha 0.83–0.89) with only marginal, statistically non-significant, improvement by select item removal.  Floor and ceiling effect:  Evaluation of floor and ceiling performance frequency was comparable between forms in each case, with 25% and 22% of SZ participant performing at floor on first administration of SAT-MC and SAT-MC-II, respectively, and 3.10% performing at ceiling on 2nd administration of both forms. A maximum of 13.30% performed at floor and ceiling across forms and administrations in SUD.  Test-retest reliability:  Test-retest reliability of SAT-MC forms was above acceptable levels in SZ (r = 0.74–0.86) and higher than obtained for SCOPE comparison measures. Scores across test sessions suggested good stability, however, the magnitude of difference was slightly larger for the SAT-MC-II, due both to higher differences in mean score and less variance. SAT-MC and SAT-MC-II reliability estimates were lower in SUD (r = 0.49–0.57), however, mean scores were highly stable across sessions.  Relationship to functioning:  Bivariate relationships between social cognitive tests and measures of interpersonal function were generally small and not statistically significant. The two SAT-MC forms were similar in strength of association across groups. |
| **MiniPONS** | | | | | |
| Banziger et al., 2011 (Bänziger et al., 2011) | Research study-Psychometric properties of MiniPONS | III | PONS and MiniPONS | 74 SCZ | Test-retest reliability:  The scores of the 65 participants who completed both tests were correlated (r = .70) when correlating Full-PONS scores with MiniPONS scores. The test– retest correlation was comparable when computing the two scores on the basis of the 64 items in the full PONS that were selected for inclusion in the MiniPONS (r = .64). The last column of Table 1 shows the test–retest correlations for the single-channel scores computed on the basis of the selected items only.  Convergent validity:  given the respectable correlation of r = .70 with the full version, the MiniPONS short form seems to capture an essential portion of the variance.  Practicality and tolerability:  The reduction of the length of the test (64-items against 220-items for the full version) necessarily results in lower reliability of the scores. |
| **Attributional bias/style** | | | | | |
| **AIHQ** | | | | | |
| Penn et al., 2005 [218] | Research study-  Psychometric properties of AIHQ | III | AIHQ | 50 SCZ | Utility as a repeated measure:  No significant improvement from SCIT in an uncontrolled open trial. |
| Waldheter et al., 2005 [248] | Research study-  Psychometric properties of AIHQ | III | AIHQ | 29 SCZ | Reliability:  ICCs for the hostility bias ranged from .87 to 1.00. Relationship to functioning: AIHQ predicted severity of violence in an inpatient unit (beta = .20, p < .05). |
| Combs et al., 2007 [177] | Research study-  Psychometric properties of AIHQ | II | AIHQ | 322 SCZ | Reliability:  Across intentional, ambiguous, and accidental situations, the average ICCs were high for both the hostility bias (range .91-99) and aggression bias ratings (range .93 - .99). Internal consistency: intentional (α = .85), ambiguous (α =.86), and accidental situations (α =.84).  Distribution:  Range of scores: hostility accidental item mean = 1.1 (SD = .22), and Aggression accidental = 1.3 (SD = .33), to Blame intentional mean = 4.3 (SD = .55). |
| Horan et al., 2009 [249] | Research study-  Psychometric properties of AIHQ | III | AIHQ | 31 SCZ | Distribution: Range of scores 1.5 (SD=.6) (hostility score) to 3.0 (SD=.8) (blame score); Utility as a repeated measure:  No impact of social cognition training or TAU on AIHQ. |
| Combs et al., 2009 [250] | Research study-  Psychometric properties of AIHQ | III | AIHQ | 50 HC, 32 SCZ with persecutory delusions, 28 SCZ without persecutory delusions | Reliability:  Raters trained to ICCs > .80; Agreement on hostility and aggression scores ranged from .80-.86; internal consistency of blame score was .74 for SCZ and .78 for HC (short version). Distribution:  HC: hostility score = 1.5 (SD=.31), blame score = 2.5 (SD=.61), aggression score = 1.4 (SD=.14) SSI with persecutory delusions: hostility bias = 2.5 (SD=.52), blame score = 3.1 (SD=.60), aggression score = 1.7 (SD=.47). Convergent validity: AIHQ hostility bias correlated with IPSAQ personalizing bias (r=.35); AIHQ aggression bias correlated with IPSAQ externalizing bias (r=.20). |
| Roberts et al., 2009 [190] | Research study-  Psychometric properties of AIHQ | III | AIHQ | 31 SCZ | Reliability:  ICC was 0.85. Cronbach’s alpha of the Likert-rated Blame scores was 0.92 (short version). Distribution:  Range of scores were from 1.51 (SD=.60) (hostility score) to 2.93 (SD=.95) (blame score).  Utility as a repeated measure:  SCIT (social cognition and interaction training) + TAU versus TAU alone: no treatment effects and no changes resulting from TAU. |
| Roberts et al., 2010 [251] | Research study-  Psychometric properties of AIHQ | III | AIHQ | 50 SCZ | Reliability:  ICCs > .75 for hostility and aggression bias ratings (short version). |
| Elnakeeb et al., 2010 [252] | Research study-  Psychometric properties of AIHQ | II | AIHQ | 150 SCZ | Reliability:  Internal consistency: Blame scores (intentional, accidental, and ambiguous situations) .81 or higher; hostility bias (.52-.63), aggression bias (.63-.70). Test-retest reliability: Blame scores (.66-.87), hostile bias (.52-64), and aggression bias (.22-.70). |
| Horan et al., 2011 [253] | Research study-  Psychometric properties of AIHQ | III | AIHQ | 111 SSI; 67 HC | Utility as a repeated measure:  Social cognition training + neurocognitive remediation = reduction in blame scores and a trend level reduction in aggression biases. |
| Mancuso et al., 2011 [43] | Research study-  Psychometric properties of AIHQ | III | AIHQ | 85 SCZ | Reliability:  ICC's for two blinded raters was > .85 (aggression and hostile biases); short version. Distribution: Range of scores were from 1.80 (SD=.53) (hostility score) to 2.97 (SD=.96) (blame score). Convergent validity: No significant correlations with emotion perception, ToM, and social perception. Discriminant validity: AIHQ factor correlated .22 (p < .05) with MATRICs cognitive batter. |
| **Trustworthiness task** | | | | | |
| Baas et al., 2008a [254] | Research study-  Psychometric properties of Trustworthiness task | III | Trustworthiness task | 18 SCZ, 24 unaffected siblings of SCZ, 28 HC | Distribution:  Overall mean: SCZ 4.4 (SD=.49); Siblings 4.3 (SD=.57); HC 4.0 (SD=.31). Untrustworthy faces mean: SCZ 4.0 (SD=.52); Siblings 3.8 (SD=.73); HC 3.5 (SD=.42). Trustworthy faces mean: SCZ 4.8 (SD=.57); Siblings 4.7 (SD=.63); HC 4.5 (SD=.34). |
| Baas et al., 2008b [255] | Research study-  Psychometric properties of Trustworthiness task | III | Trustworthiness task | 12 SCZ, 21 HC | Distributions:  Overall mean: SCZ 3.88 (SD=.17); HC 3.94 (SD=.13). |
| Couture et al., 2008 [241] | Research study-  Psychometric properties of Trustworthiness task | III | Trustworthiness task  Eyes task | 26 young SCZ 88 CHR 41 HC | **Trustworthiness task** Distributions: Untrustworthy faces mean: CHR -.70 (SD=.84), SSI -.86 (SD=.87), HC -1.21 (SD=.54); Trustworthy faces mean: CHR 1.28 (SD=.77), SSI 1.46 (SD=.82), HC 1.34 (SD=.66). Convergent validity: ns with Eyes Task. |
| Pinkham et al., 2008 [256] | Research study-  Psychometric properties of Trustworthiness task | III | Trustworthiness task | 12 P-SCZ, 12 NP-SCZ and 12 HC | Reliability:  ICC across groups = .924; Distributions: Mean % rated as trustworthy (binary yes or no response): ASD 54.4% (SD=15.3%), P-SCZ 46.3% (SD=14.3%), NP-SCZ 60.3% (SD=13.3%), HC 60.7% (SD=11.9%). |
| Couture et al., 2010 [257] | Research study-  Psychometric properties of Trustworthiness task | III | Trustworthiness task | 44 SCZ, 36 HFA, 41 HC | Distributions: Untrustworthy faces mean: HC -1.22 (SD=.52); HFA -.71 (SD=.98); SCZ -.82 (SD=1.10); SCZ-Negative (n=13) -.64 (SD=1.3), SCZ-paranoid (n=8) -1.41 (SD=1.15). Trustworthy faces mean: HC 1.31 (SD=.65); HFA 1.37 (SD=.82); SCZ 1.41 (SD=.93); SCZ-Negative (n=13) 1.55 (SD=.68), SCZ-Paranoid (n=8) .63 (SD=1.04). |
| Pedersen et al., 2011 [258] | Research study-  Psychometric properties of Trustworthiness task | III | Trustworthiness task | 20 SCZ (11 in oxytocin treatment, 9 in placebo) | Distributions: Untrustworthy faces mean: Placebo group Time 1 -.06 (SD=.24); Time 2 -.12 (SD=.24); Treatment group Time 1 -.31 (SD=.30); Time 2 -.18 (SD=.24).  Utility as a repeated measure:  Untrustworthy faces mean: Placebo group Time 1 -.06 (SD=.24); Time 2 -.12 (SD=.24); Treatment group Time 1 -.31 (SD=.30); Time 2 -.18 (SD=.24); Placebo group difference not significant, treatment group trends toward significant difference (p≤.08). |
| **IBT** | | | | | |
| Buck et al., 2018 [259] | Research study-  Psychometric properties of IBT | II | IBT | 213 SCZ and 151 HC | SCZ were more likely to attribute intentional motives to others’ actions relative to controls. This intentionality bias was related to hostility, role functioning, and independent living skills.  While HC estimates were positively correlated with all five SCOPE measures of social cognitive skill, automatic bias estimates and total scores were significantly negatively related with one measure of emotion perception (ER-40).  Relationship to functioning:  an increased ability to control responses on the IBT is related to social cognitive skill, and an overall tendency to see more intentionality may be related to emotion perception. The IBT total score was related to a number of the criterion functional outcomes; higher levels of intentionality bias correlated only with increased trait hostility. The IBT was related to a number of general social and role functioning outcomes, as total scores correlated with role functioning, social functioning, and functional capacity; automatic estimates negatively correlated with functional capacity, while the control estimate was positively related to social skills and independent living skills. |
| **All domains** | | | | | |
| Pinkham et al., 2014 [260] | SCOPE Initiative | II | BLERT  ER-40  Hinting task  TASIT  RAD  AIHQ  Trustworthiness Task | Two surveys and RAND Panel | Expert surveys identified 4 core domains of social cognition: emotion processing, social perception, theory of mind/mental state attribution, and attributional style/bias. Using RAND panel consensus ratings, the following measures were selected for further evaluation: BLERT, ER-40, Hinting task, TASIT, RAD, AIHQ and Trustworthiness Task. |
| Pinkham et al., 2016 [261] | Psychometric properties of SCOPE Initiative measures | II | BLERT  ER-40  Hinting task  TASIT  RAD  AIHQ | 179 SCZ and 104 HC | **BLERT** showed good reliability, utility as a repeated measure, relationship to functioning, social competence and functional capacity, practicality and tolerability and good capacity to difference patients with control;  **Hinting task** showed good reliability, utility as a repeated measure, relationship to functioning, social competence and functional capacity, practicality and tolerability and good capacity to difference patients with control;  **ER-40** showed good reliability, utility as a repeated measure, relationship to social competence and functional capacity, practicality and tolerability and good capacity to difference patients with control;  **Eyes task** showed good reliability, utility as a repeated measure, relationship to social competence and functional capacity, practicality and tolerability and good capacity to difference patients with control;  **TASIT** showed good reliability, relationship to functioning, social competence and functional capacity, good capacity to difference patients with control but is too long;  **RAD** showed good reliability, relationship to functioning, social competence and functional capacity, good capacity to difference patients with control but is too long;  **AIHQ** showed low reliability, low utility as a repeated measure, no relationship to functioning, social competence and functional capacity, good practicality and tolerability and capacity to difference patients with control;  **Trustworthiness task** showed good reliability, no relationship to functioning, social competence and functional capacity, good practicality and tolerability and capacity to difference patients with control. |
| Pinkham et al., 2018 [262] | Psychometric properties of SCOPE Initiative measures | II | BLERT  ER-40  Hinting task  TASIT  RAD  Mini-PONS  SAT-MC  IBT  BLERT and ER-40 modifications included response time and confidence ratings. The Eyes task was modified to include definitions of terms and TASIT to include response time. Hinting was scored with more stringent criteria. Mini-PONS, SAT-MC, and IBT were new to this phase. | 218 SCZ and 154 HC | **BLERT** showed good reliability, utility as a repeated measure, relationship to functioning, social competence and functional capacity, practicality and tolerability and good capacity to difference patients with control;  **Hinting task** showed good reliability, utility as a repeated measure, relationship to functioning, social competence and functional capacity, practicality and tolerability and good capacity to difference patients with control;  **ER-40** showed good reliability, utility as a repeated measure, relationship to social competence and functional capacity, practicality and tolerability and good capacity to difference patients with control;  **Eyes task** showed good reliability, utility as a repeated measure, relationship to social competence and functional capacity, practicality and tolerability and good capacity to difference patients with control;  **TASIT** showed good reliability, utility as a repeated measure, relationship to social competence and functional capacity, good capacity to difference patients with control but is too long;  **IBT** showed low reliability, utility as a repeated measure, relationship to functioning and functional capacity, good practicality and tolerability and good capacity to difference patients with control;  **MiniPONS** showed good reliability, utility as a repeated measure, relationship to social competence and functional capacity, good practicality and tolerability and good capacity to difference patients with control;  **SAT-MC** showed good internal consistency but low test-retest reliability, good utility as a repeated measure, relationship to social competence and functional capacity, good practicality and tolerability and good capacity to difference patients with control. |
| Lim et al., 2020 [159] | Psychometric properties of SCOPE Initiative measures | II | BLERT  Hinting task  ER-40  TASIT  RAD  AIHQ  MSCEIT  Mini-PONS  IPSAQ | 116 SCZ and 73 HC | BLERT, ER40 and TASIT Part III showed strongest psychometrics.  AIHQ-HB showed slightly weaker properties, requiring further evaluation. The Hinting task, MiniPONS, RAD, IPSAQ, and MSCEIT showed poorer psychometrics. PCA revealed a two-factor solution comprising social cognition skills and attributional style/bias. |

**AIPSS:** Assessment of Interpersonal Problem Solving Skills; **AIHQ:** The Ambiguous Intentions Hostility Questionnaire; **AIPPS**: The Assessment of Interpersonal Problem-Solving Skills**; AS**: Aspergers Syndrome; **BD**: Bipolar disorder; **BLERT**: The Bell Lysaker Emotion Recognition Task; **BORRTI**: Bell Object Relations Reality Testing Inventory; **CJOLO**: Computerized judgment of line orientation; **CHR**: Clinical High Risk; **CNB:** Penn's Computerized Neurocognitive Battery; **CRT**: cognitive remediation training; **DANVA**: Diagnostic Analysis of Nonverbal Accuracy; **EQ-i:** Bar-On Emotional Intelligence Inventory; **ER-40**: The Penn Emotion Recognition Task; **ERI**: Emotion Recognition Index; **FEDT**: Face Emotion Discrimination Test; **FEIT**: Facial Emotion Identification Test; **HB**: Hostility bias; **HC**: healthy controls; **HFA**: High functioning autism; **JACFEE**: Japanese and Caucasian facial expressions of emotion; **IBT**: The Intentional Bias Task; **ICC**: Intraclass correlation; **ILSS**: Independent Living Skills Survey; **IPSA-Q**: Internal, Personal and Situational Attributions Questionnaire; **MERT**: The Multimodal Emotion Recognition Test; **Mini-PONS**: The Mini Profile of Nonverbal Sensitivity; **MMAA:** Medication Management Ability Assessment; **MMLT**: Micro-Module Learning Tests; **MRIGE**: Mind Reading: Interactive Guide to Emotions; **MSCEIT**: The Mayer-Salovey-Caruso Emotional Intelligence Test; **NET**: neurocognitive enhancement training; **NOS**: not otherwise specified; **NOSIE**: Nurse's Observation Scale for Inpatient Evaluation; **ns**: not significant; **P-SCZ**: subjects with paranoid schizophrenia; **NP-SCZ**: subjects with non-paranoid schizophrenia; **PCET**: Penn Conditional Exclusion Test; **PVRT**: Penn Verbal Reasoning Test (PVRT); **QLS**: Quality of Life Scale; **RAD**: Relationships Across Domains; **SAT-MC**: The Social Attribution Task—Multiple Choice version; **SCIT**: Social cognition and interaction training; **SCOPE:** Social Cognition Psychometric Evaluation; **SCST**: The Schema Component Sequencing Task; **SCZ**: subjects with chronic schizophrenia; **SCZaff**: subjects with schizoaffective disorder; **SD**: standard deviation; **SREIT**: Self-Report Emotional Intelligence Test; **SSPA:** Social Skill Performance Assessment; **SUD**: substance use disorder; **TASIT**: The Awareness of Social Inference Test; **TAS-20K:** Toronto Alexithymia Scale; **TAU**: treatment as usual; **UOT**: Unexpected Outcomes Test; **UPSA**: UCSD Performance-Based Skill Assessment; **WRAT**: Wide Range Achievement Test; **WT**: work therapy.

**References**

[1] Fioravanti M, Carlone O, Vitale B, Cinti ME, Clare L. A meta-analysis of cognitive deficits in adults with a diagnosis of schizophrenia. Neuropsychol Rev 2005;15:73–95. https://doi.org/10.1007/s11065-005-6254-9.

[2] Dickinson D, Ramsey ME, Gold JM. Overlooking the obvious: a meta-analytic comparison of digit symbol coding tasks and other cognitive measures in schizophrenia. Arch Gen Psychiatry 2007;64:532–42. https://doi.org/10.1001/archpsyc.64.5.532.

[3] Sprong M, Schothorst P, Vos E, Hox J, van Engeland H. Theory of mind in schizophrenia: meta-analysis. Br J Psychiatry J Ment Sci 2007;191:5–13. https://doi.org/10.1192/bjp.bp.107.035899.

[4] Hoekert M, Kahn RS, Pijnenborg M, Aleman A. Impaired recognition and expression of emotional prosody in schizophrenia: review and meta-analysis. Schizophr Res 2007;96:135–45. https://doi.org/10.1016/j.schres.2007.07.023.

[5] Bora E, Yucel M, Pantelis C. Theory of mind impairment in schizophrenia: meta-analysis. Schizophr Res 2009;109:1–9. https://doi.org/10.1016/j.schres.2008.12.020.

[6] Forbes NF, Carrick LA, McIntosh AM, Lawrie SM. Working memory in schizophrenia: a meta-analysis. Psychol Med 2009;39:889–905. https://doi.org/10.1017/S0033291708004558.

[7] Doughty OJ, Done DJ. Is semantic memory impaired in schizophrenia? A systematic review and meta-analysis of 91 studies. Cognit Neuropsychiatry 2009;14:473–509. https://doi.org/10.1080/13546800903073291.

[8] Chan RCK, Li H, Cheung EFC, Gong Q-Y. Impaired facial emotion perception in schizophrenia: a meta-analysis. Psychiatry Res 2010;178:381–90. https://doi.org/10.1016/j.psychres.2009.03.035.

[9] Knowles EEM, David AS, Reichenberg A. Processing speed deficits in schizophrenia: reexamining the evidence. Am J Psychiatry 2010;167:828–35. https://doi.org/10.1176/appi.ajp.2010.09070937.

[10] Kohler CG, Walker JB, Martin EA, Healey KM, Moberg PJ. Facial emotion perception in schizophrenia: a meta-analytic review. Schizophr Bull 2010;36:1009–19. https://doi.org/10.1093/schbul/sbn192.

[11] Westerhausen R, Kompus K, Hugdahl K. Impaired cognitive inhibition in schizophrenia: a meta-analysis of the Stroop interference effect. Schizophr Res 2011;133:172–81. https://doi.org/10.1016/j.schres.2011.08.025.

[12] Fioravanti M, Bianchi V, Cinti ME. Cognitive deficits in schizophrenia: an updated metanalysis of the scientific evidence. BMC Psychiatry 2012;12:64. https://doi.org/10.1186/1471-244X-12-64.

[13] Savla GN, Vella L, Armstrong CC, Penn DL, Twamley EW. Deficits in domains of social cognition in schizophrenia: a meta-analysis of the empirical evidence. Schizophr Bull 2013;39:979–92. https://doi.org/10.1093/schbul/sbs080.

[14] Bora E, Pantelis C. Theory of mind impairments in first-episode psychosis, individuals at ultra-high risk for psychosis and in first-degree relatives of schizophrenia: systematic review and meta-analysis. Schizophr Res 2013;144:31–6. https://doi.org/10.1016/j.schres.2012.12.013.

[15] Fatouros-Bergman H, Cervenka S, Flyckt L, Edman G, Farde L. Meta-analysis of cognitive performance in drug-naïve patients with schizophrenia. Schizophr Res 2014;158:156–62. https://doi.org/10.1016/j.schres.2014.06.034.

[16] Lee TY, Hong SB, Shin NY, Kwon JS. Social cognitive functioning in prodromal psychosis: A meta-analysis. Schizophr Res 2015;164:28–34. https://doi.org/10.1016/j.schres.2015.02.008.

[17] Berna F, Potheegadoo J, Aouadi I, Ricarte JJ, Allé MC, Coutelle R, et al. A Meta-Analysis of Autobiographical Memory Studies in Schizophrenia Spectrum Disorder. Schizophr Bull 2016;42:56–66. https://doi.org/10.1093/schbul/sbv099.

[18] Grimes KM, Zanjani A, Zakzanis KK. Memory impairment and the mediating role of task difficulty in patients with schizophrenia. Psychiatry Clin Neurosci 2017;71:600–11. https://doi.org/10.1111/pcn.12520.

[19] Knapp F, Viechtbauer W, Leonhart R, Nitschke K, Kaller CP. Planning performance in schizophrenia patients: a meta-analysis of the influence of task difficulty and clinical and sociodemographic variables. Psychol Med 2017;47:2002–16. https://doi.org/10.1017/S0033291717000459.

[20] Watson AJ, Joyce EM, Fugard AJB, Leeson VC, Barnes TRE, Huddy V. More haste less speed: A meta-analysis of thinking latencies during planning in people with psychosis. Psychiatry Res 2017;258:576–82. https://doi.org/10.1016/j.psychres.2017.09.003.

[21] Thai ML, Andreassen AK, Bliksted V. A meta-analysis of executive dysfunction in patients with schizophrenia: Different degree of impairment in the ecological subdomains of the Behavioural Assessment of the Dysexecutive Syndrome. Psychiatry Res 2019;272:230–6. https://doi.org/10.1016/j.psychres.2018.12.088.

[22] Zhang H, Wang Y, Hu Y, Zhu Y, Zhang T, Wang J, et al. Meta-analysis of cognitive function in Chinese first-episode schizophrenia: MATRICS Consensus Cognitive Battery (MCCB) profile of impairment. Gen Psychiatry 2019;32:e100043. https://doi.org/10.1136/gpsych-2018-100043.

[23] Mirsky AF. Behavioral and psychophysiological markers of disordered attention. Environ Health Perspect 1987;74:191–9. https://doi.org/10.1289/ehp.8774191.

[24] Kremen WS, Seidman LJ, Faraone SV, Pepple JR, Tsuang MT. Attention/information-processing factors in psychotic disorders. Replication and extension of recent neuropsychological findings. J Nerv Ment Dis 1992;180:89–93. https://doi.org/10.1097/00005053-199202000-00004.

[25] Allen DN, Huegel SG, Seaton BE, Goldstein G, Gurklis JA, van Kammen DP. Confirmatory factor analysis of the WAIS-R in patients with schizophrenia. Schizophr Res 1998;34:87–94. https://doi.org/10.1016/s0920-9964(98)00090-5.

[26] Hobart MP, Goldberg R, Bartko JJ, Gold JM. Repeatable battery for the assessment of neuropsychological status as a screening test in schizophrenia, II: convergent/discriminant validity and diagnostic group comparisons. Am J Psychiatry 1999;156:1951–7. https://doi.org/10.1176/ajp.156.12.1951.

[27] Kurtz MM, Ragland JD, Bilker W, Gur RC, Gur RE. Comparison of the continuous performance test with and without working memory demands in healthy controls and patients with schizophrenia. Schizophr Res 2001;48:307–16. https://doi.org/10.1016/s0920-9964(00)00060-8.

[28] Green MF, Marder SR, Glynn SM, McGurk SR, Wirshing WC, Wirshing DA, et al. The neurocognitive effects of low-dose haloperidol: a two-year comparison with risperidone. Biol Psychiatry 2002;51:972–8. https://doi.org/10.1016/s0006-3223(02)01370-7.

[29] Friis S, Sundet K, Rund BR, Vaglum P, McGlashan TH. Neurocognitive dimensions characterising patients with first-episode psychosis. Br J Psychiatry Suppl 2002;43:s85-90. https://doi.org/10.1192/bjp.181.43.s85.

[30] Dickinson D, Iannone VN, Wilk CM, Gold JM. General and specific cognitive deficits in schizophrenia. Biol Psychiatry 2004;55:826–33. https://doi.org/10.1016/j.biopsych.2003.12.010.

[31] Gladsjo JA, McAdams LA, Palmer BW, Moore DJ, Jeste DV, Heaton RK. A six-factor model of cognition in schizophrenia and related psychotic disorders: relationships with clinical symptoms and functional capacity. Schizophr Bull 2004;30:739–54. https://doi.org/10.1093/oxfordjournals.schbul.a007127.

[32] Keefe RSE, Goldberg TE, Harvey PD, Gold JM, Poe MP, Coughenour L. The Brief Assessment of Cognition in Schizophrenia: reliability, sensitivity, and comparison with a standard neurocognitive battery. Schizophr Res 2004;68:283–97. https://doi.org/10.1016/j.schres.2003.09.011.

[33] Dickinson D, Ragland JD, Calkins ME, Gold JM, Gur RC. A comparison of cognitive structure in schizophrenia patients and healthy controls using confirmatory factor analysis. Schizophr Res 2006;85:20–9. https://doi.org/10.1016/j.schres.2006.03.003.

[34] Keefe RSE, Bilder RM, Harvey PD, Davis SM, Palmer BW, Gold JM, et al. Baseline neurocognitive deficits in the CATIE schizophrenia trial. Neuropsychopharmacol Off Publ Am Coll Neuropsychopharmacol 2006;31:2033–46. https://doi.org/10.1038/sj.npp.1301072.

[35] Genderson MR, Dickinson D, Diaz-Asper CM, Egan MF, Weinberger DR, Goldberg TE. Factor analysis of neurocognitive tests in a large sample of schizophrenic probands, their siblings, and healthy controls. Schizophr Res 2007;94:231–9. https://doi.org/10.1016/j.schres.2006.12.031.

[36] Noh J, Kim J-H, Hong KS, Kim N, Nam HJ, Lee D, et al. Factor structure of the neurocognitive tests: an application of the confirmative factor analysis in stabilized schizophrenia patients. J Korean Med Sci 2010;25:276–82. https://doi.org/10.3346/jkms.2010.25.2.276.

[37] Dickinson D, Goldberg TE, Gold JM, Elvevåg B, Weinberger DR. Cognitive factor structure and invariance in people with schizophrenia, their unaffected siblings, and controls. Schizophr Bull 2011;37:1157–67. https://doi.org/10.1093/schbul/sbq018.

[38] Ojeda N, Peña J, Schretlen DJ, Sánchez P, Aretouli E, Elizagárate E, et al. Hierarchical structure of the cognitive processes in schizophrenia: the fundamental role of processing speed. Schizophr Res 2012;135:72–8. https://doi.org/10.1016/j.schres.2011.12.004.

[39] Burton CZ, Vella L, Harvey PD, Patterson TL, Heaton RK, Twamley EW. Factor structure of the MATRICS Consensus Cognitive Battery (MCCB) in schizophrenia. Schizophr Res 2013;146:244–8. https://doi.org/10.1016/j.schres.2013.02.026.

[40] Lam M, Collinson SL, Eng GK, Rapisarda A, Kraus M, Lee J, et al. Refining the latent structure of neuropsychological performance in schizophrenia. Psychol Med 2014;44:3557–70. https://doi.org/10.1017/S0033291714001020.

[41] McCleery A, Green MF, Hellemann GS, Baade LE, Gold JM, Keefe RSE, et al. Latent structure of cognition in schizophrenia: a confirmatory factor analysis of the MATRICS Consensus Cognitive Battery (MCCB). Psychol Med 2015;45:2657–66. https://doi.org/10.1017/S0033291715000641.

[42] Lo SB, Szuhany KL, Kredlow MA, Wolfe R, Mueser KT, McGurk SR. A confirmatory factor analysis of the MATRICS consensus cognitive battery in severe mental illness. Schizophr Res 2016;175:79–84. https://doi.org/10.1016/j.schres.2016.03.013.

[43] Mancuso F, Horan WP, Kern RS, Green MF. Social cognition in psychosis: multidimensional structure, clinical correlates, and relationship with functional outcome. Schizophr Res 2011;125:143–51. https://doi.org/10.1016/j.schres.2010.11.007.

[44] Mehta UM, Thirthalli J, Subbakrishna DK, Gangadhar BN, Eack SM, Keshavan MS. Social and neuro-cognition as distinct cognitive factors in schizophrenia: a systematic review. Schizophr Res 2013;148:3–11. https://doi.org/10.1016/j.schres.2013.05.009.

[45] Corbera S, Wexler BE, Ikezawa S, Bell MD. Factor structure of social cognition in schizophrenia: is empathy preserved? Schizophr Res Treat 2013;2013:409205. https://doi.org/10.1155/2013/409205.

[46] Buck BE, Healey KM, Gagen EC, Roberts DL, Penn DL. Social cognition in schizophrenia: factor structure, clinical and functional correlates. J Ment Health Abingdon Engl 2016;25:330–7. https://doi.org/10.3109/09638237.2015.1124397.

[47] Browne J, Penn DL, Raykov T, Pinkham AE, Kelsven S, Buck B, et al. Social cognition in schizophrenia: Factor structure of emotion processing and theory of mind. Psychiatry Res 2016;242:150–6. https://doi.org/10.1016/j.psychres.2016.05.034.

[48] Mike L, Guimond S, Kelly S, Thermenos H, Mesholam-Gately R, Eack S, et al. Social cognition in early course of schizophrenia: Exploratory factor analysis. Psychiatry Res 2019;272:737–43. https://doi.org/10.1016/j.psychres.2018.12.152.

[49] Green MF, Kern RS, Braff DL, Mintz J. Neurocognitive deficits and functional outcome in schizophrenia: are we measuring the “right stuff”? Schizophr Bull 2000;26:119–36. https://doi.org/10.1093/oxfordjournals.schbul.a033430.

[50] Green MF, Kern RS, Heaton RK. Longitudinal studies of cognition and functional outcome in schizophrenia: implications for MATRICS. Schizophr Res 2004;72:41–51. https://doi.org/10.1016/j.schres.2004.09.009.

[51] Sergi MJ, Rassovsky Y, Nuechterlein KH, Green MF. Social perception as a mediator of the influence of early visual processing on functional status in schizophrenia. Am J Psychiatry 2006;163:448–54. https://doi.org/10.1176/appi.ajp.163.3.448.

[52] Bowie CR, Leung WW, Reichenberg A, McClure MM, Patterson TL, Heaton RK, et al. Predicting schizophrenia patients’ real-world behavior with specific neuropsychological and functional capacity measures. Biol Psychiatry 2008;63:505–11. https://doi.org/10.1016/j.biopsych.2007.05.022.

[53] Ventura J, Hellemann GS, Thames AD, Koellner V, Nuechterlein KH. Symptoms as mediators of the relationship between neurocognition and functional outcome in schizophrenia: a meta-analysis. Schizophr Res 2009;113:189–99. https://doi.org/10.1016/j.schres.2009.03.035.

[54] Addington J, Girard TA, Christensen BK, Addington D. Social cognition mediates illness-related and cognitive influences on social function in patients with schizophrenia-spectrum disorders. J Psychiatry Neurosci JPN 2010;35:49–54. https://doi.org/10.1503/jpn.080039.

[55] Fett A-KJ, Viechtbauer W, Dominguez M-G, Penn DL, van Os J, Krabbendam L. The relationship between neurocognition and social cognition with functional outcomes in schizophrenia: a meta-analysis. Neurosci Biobehav Rev 2011;35:573–88. https://doi.org/10.1016/j.neubiorev.2010.07.001.

[56] Nuechterlein KH, Subotnik KL, Green MF, Ventura J, Asarnow RF, Gitlin MJ, et al. Neurocognitive predictors of work outcome in recent-onset schizophrenia. Schizophr Bull 2011;37 Suppl 2:S33-40. https://doi.org/10.1093/schbul/sbr084.

[57] Schmidt SJ, Mueller DR, Roder V. Social cognition as a mediator variable between neurocognition and functional outcome in schizophrenia: empirical review and new results by structural equation modeling. Schizophr Bull 2011;37 Suppl 2:S41-54. https://doi.org/10.1093/schbul/sbr079.

[58] Green MF, Bearden CE, Cannon TD, Fiske AP, Hellemann GS, Horan WP, et al. Social cognition in schizophrenia, Part 1: performance across phase of illness. Schizophr Bull 2012;38:854–64. https://doi.org/10.1093/schbul/sbq171.

[59] Horan WP, Green MF, DeGroot M, Fiske A, Hellemann G, Kee K, et al. Social cognition in schizophrenia, Part 2: 12-month stability and prediction of functional outcome in first-episode patients. Schizophr Bull 2012;38:865–72. https://doi.org/10.1093/schbul/sbr001.

[60] Irani F, Seligman S, Kamath V, Kohler C, Gur RC. A meta-analysis of emotion perception and functional outcomes in schizophrenia. Schizophr Res 2012;137:203–11. https://doi.org/10.1016/j.schres.2012.01.023.

[61] Ho JS, Moore RC, Davine T, Cardenas V, Bowie CR, Patterson TL, et al. Direct and mediated effects of cognitive function with multidimensional outcome measures in schizophrenia: the role of functional capacity. J Clin Exp Neuropsychol 2013;35:882–95. https://doi.org/10.1080/13803395.2013.828021.

[62] Galderisi S, Rossi A, Rocca P, Bertolino A, Mucci A, Bucci P, et al. The influence of illness-related variables, personal resources and context-related factors on real-life functioning of people with schizophrenia. World Psychiatry Off J World Psychiatr Assoc WPA 2014;13:275–87. https://doi.org/10.1002/wps.20167.

[63] Lepage M, Bodnar M, Bowie CR. Neurocognition: clinical and functional outcomes in schizophrenia. Can J Psychiatry Rev Can Psychiatr 2014;59:5–12. https://doi.org/10.1177/070674371405900103.

[64] Moore RC, Harmell AL, Harvey PD, Bowie CR, Depp CA, Pulver AE, et al. Improving the understanding of the link between cognition and functional capacity in schizophrenia and bipolar disorder. Schizophr Res 2015;169:121–7. https://doi.org/10.1016/j.schres.2015.09.017.

[65] Strassnig MT, Raykov T, O’Gorman C, Bowie CR, Sabbag S, Durand D, et al. Determinants of different aspects of everyday outcome in schizophrenia: The roles of negative symptoms, cognition, and functional capacity. Schizophr Res 2015;165:76–82. https://doi.org/10.1016/j.schres.2015.03.033.

[66] Bechi M, Bosia M, Spangaro M, Buonocore M, Cavedoni S, Agostoni G, et al. Exploring functioning in schizophrenia: Predictors of functional capacity and real-world behaviour. Psychiatry Res 2017;251:118–24. https://doi.org/10.1016/j.psychres.2017.02.019.

[67] Galderisi S, Rucci P, Kirkpatrick B, Mucci A, Gibertoni D, Rocca P, et al. Interplay Among Psychopathologic Variables, Personal Resources, Context-Related Factors, and Real-life Functioning in Individuals With Schizophrenia: A Network Analysis. JAMA Psychiatry 2018;75:396–404. https://doi.org/10.1001/jamapsychiatry.2017.4607.

[68] Strassnig M, Bowie C, Pinkham AE, Penn D, Twamley EW, Patterson TL, et al. Which levels of cognitive impairments and negative symptoms are related to functional deficits in schizophrenia? J Psychiatr Res 2018;104:124–9. https://doi.org/10.1016/j.jpsychires.2018.06.018.

[69] Bosia M, Bechi M, Bosinelli F, Politi E, Buonocore M, Spangaro M, et al. From cognitive and clinical substrates to functional profiles: Disentangling heterogeneity in schizophrenia. Psychiatry Res 2019;271:446–53. https://doi.org/10.1016/j.psychres.2018.12.026.

[70] Halverson TF, Orleans-Pobee M, Merritt C, Sheeran P, Fett A-K, Penn DL. Pathways to functional outcomes in schizophrenia spectrum disorders: Meta-analysis of social cognitive and neurocognitive predictors. Neurosci Biobehav Rev 2019;105:212–9. https://doi.org/10.1016/j.neubiorev.2019.07.020.

[71] Ojeda N, Sánchez P, Gómez-Gastiasoro A, Peña J, Elizagárate E, Ezcurra J, et al. An outcome prediction model for schizophrenia: A structural equation modelling approach. Rev Psiquiatr Salud Ment 2019;12:232–41. https://doi.org/10.1016/j.rpsm.2019.01.007.

[72] Amoretti S, Rosa AR, Mezquida G, Cabrera B, Ribeiro M, Molina M, et al. The impact of cognitive reserve, cognition and clinical symptoms on psychosocial functioning in first-episode psychoses. Psychol Med 2020:1–12. https://doi.org/10.1017/S0033291720002226.

[73] González-Ortega I, González-Pinto A, Alberich S, Echeburúa E, Bernardo M, Cabrera B, et al. Influence of social cognition as a mediator between cognitive reserve and psychosocial functioning in patients with first episode psychosis. Psychol Med 2020;50:2702–10. https://doi.org/10.1017/S0033291719002794.

[74] Hochberger WC, Thomas ML, Joshi YB, Swerdlow NR, Braff DL, Gur RE, et al. Deviation from expected cognitive ability is a core cognitive feature of schizophrenia related to neurophysiologic, clinical and psychosocial functioning. Schizophr Res 2020;215:300–7. https://doi.org/10.1016/j.schres.2019.10.011.

[75] Lewandowski KE, Cohen TR, Ongur D. Cognitive and clinical predictors of community functioning across the psychoses. PsyCh J 2020;9:163–73. https://doi.org/10.1002/pchj.356.

[76] Modinos G, Kempton MJ, Tognin S, Calem M, Porffy L, Antoniades M, et al. Association of Adverse Outcomes With Emotion Processing and Its Neural Substrate in Individuals at Clinical High Risk for Psychosis. JAMA Psychiatry 2020;77:190–200. https://doi.org/10.1001/jamapsychiatry.2019.3501.

[77] Rojnic Kuzman M, Makaric P, Bosnjak Kuharic D, Kekin I, Madzarac Z, Koricancic Makar A, et al. General Functioning in Patients With First-Episode Psychosis After the First 18 Months of Treatment. J Clin Psychopharmacol 2020;40:366–72. https://doi.org/10.1097/JCP.0000000000001224.

[78] Salagre E, Grande I, Solé B, Mezquida G, Cuesta MJ, Díaz-Caneja CM, et al. Exploring Risk and Resilient Profiles for Functional Impairment and Baseline Predictors in a 2-Year Follow-Up First-Episode Psychosis Cohort Using Latent Class Growth Analysis. J Clin Med 2020;10:E73. https://doi.org/10.3390/jcm10010073.

[79] Vita A, Barlati S, Deste G, Rocca P, Rossi A, Bertolino A, et al. The influence of autistic symptoms on social and non-social cognition and on real-life functioning in people with schizophrenia: Evidence from the Italian Network for Research on Psychoses multicenter study. Eur Psychiatry J Assoc Eur Psychiatr 2020;63:e98. https://doi.org/10.1192/j.eurpsy.2020.99.

[80] Ritsner MS. Predicting quality of life impairment in chronic schizophrenia from cognitive variables. Qual Life Res Int J Qual Life Asp Treat Care Rehabil 2007;16:929–37. https://doi.org/10.1007/s11136-007-9195-3.

[81] Lipkovich IA, Deberdt W, Csernansky JG, Sabbe B, Keefe RS, Kollack-Walker S. Relationships among neurocognition, symptoms and functioning in patients with schizophrenia: a path-analytic approach for associations at baseline and following 24 weeks of antipsychotic drug therapy. BMC Psychiatry 2009;9:44. https://doi.org/10.1186/1471-244X-9-44.

[82] Tomida K, Takahashi N, Saito S, Maeno N, Iwamoto K, Yoshida K, et al. Relationship of psychopathological symptoms and cognitive function to subjective quality of life in patients with chronic schizophrenia. Psychiatry Clin Neurosci 2010;64:62–9. https://doi.org/10.1111/j.1440-1819.2009.02033.x.

[83] Woon PS, Chia MY, Chan WY, Sim K. Neurocognitive, clinical and functional correlates of subjective quality of life in Asian outpatients with schizophrenia. Prog Neuropsychopharmacol Biol Psychiatry 2010;34:463–8. https://doi.org/10.1016/j.pnpbp.2010.01.014.

[84] Kurtz MM, Tolman A. Neurocognition, insight into illness and subjective quality-of-life in schizophrenia: what is their relationship? Schizophr Res 2011;127:157–62. https://doi.org/10.1016/j.schres.2010.12.004.

[85] Maat A, Fett A-K, Derks E, GROUP Investigators. Social cognition and quality of life in schizophrenia. Schizophr Res 2012;137:212–8. https://doi.org/10.1016/j.schres.2012.02.017.

[86] Tolman AW, Kurtz MM. Neurocognitive predictors of objective and subjective quality of life in individuals with schizophrenia: a meta-analytic investigation. Schizophr Bull 2012;38:304–15. https://doi.org/10.1093/schbul/sbq077.

[87] Cruz BF, Resende CB de, Carvalhaes CF, Cardoso CS, Teixeira AL, Keefe RS, et al. Interview-based assessment of cognition is a strong predictor of quality of life in patients with schizophrenia and severe negative symptoms. Rev Bras Psiquiatr Sao Paulo Braz 1999 2016;38:216–21. https://doi.org/10.1590/1516-4446-2015-1776.

[88] Shin Y-J, Joo Y-H, Kim J-H. Self-perceived cognitive deficits and their relationship with internalized stigma and quality of life in patients with schizophrenia. Neuropsychiatr Dis Treat 2016;12:1411–7. https://doi.org/10.2147/NDT.S108537.

[89] Takahashi T, Higuchi Y, Komori Y, Nishiyama S, Nakamura M, Sasabayashi D, et al. Quality of life in individuals with attenuated psychotic symptoms: Possible role of anxiety, depressive symptoms, and socio-cognitive impairments. Psychiatry Res 2017;257:431–7. https://doi.org/10.1016/j.psychres.2017.08.024.

[90] Lu L, Zeng L-N, Zong Q-Q, Rao W-W, Ng CH, Ungvari GS, et al. Quality of life in Chinese patients with schizophrenia: A meta-analysis. Psychiatry Res 2018;268:392–9. https://doi.org/10.1016/j.psychres.2018.07.003.

[91] Kurtz MM, Gopal S, John S, Thara R. Objective psychosocial function vs. subjective quality-of-life in schizophrenia within 5-years after diagnosis: A study from southern India. Psychiatry Res 2019;272:419–24. https://doi.org/10.1016/j.psychres.2018.12.149.

[92] Ehrminger M, Roux P, Urbach M, André M, Aouizerate B, Berna F, et al. The puzzle of quality of life in schizophrenia: putting the pieces together with the FACE-SZ cohort. Psychol Med 2020:1–8. https://doi.org/10.1017/S0033291720003311.

[93] Hoertel N, Rotenberg L, Blanco C, Camus V, Dubertret C, Charlot V, et al. A comprehensive model of predictors of quality of life in older adults with schizophrenia: results from the CSA study. Soc Psychiatry Psychiatr Epidemiol 2021;56:1411–25. https://doi.org/10.1007/s00127-020-01880-2.

[94] Kurtz MM, Bronfeld M, Rose J. Cognitive and social cognitive predictors of change in objective versus subjective quality-of-life in rehabilitation for schizophrenia. Psychiatry Res 2012;200:102–7. https://doi.org/10.1016/j.psychres.2012.06.025.

[95] Tas C, Brown E, Cubukcuoglu Z, Aydemir O, Danaci AE, Brüne M. Towards an integrative approach to understanding quality of life in schizophrenia: the role of neurocognition, social cognition, and psychopathology. Compr Psychiatry 2013;54:262–8. https://doi.org/10.1016/j.comppsych.2012.08.001.

[96] Hasson-Ohayon I, Avidan-Msika M, Mashiach-Eizenberg M, Kravetz S, Rozencwaig S, Shalev H, et al. Metacognitive and social cognition approaches to understanding the impact of schizophrenia on social quality of life. Schizophr Res 2015;161:386–91. https://doi.org/10.1016/j.schres.2014.11.008.

[97] Martín Contero MC, Secades Villa R, López Goñi JJ, Tirapu Ustarroz J. [Empathy, social cognition and subjective quality of life in schizophrenia]. An Sist Sanit Navar 2017;40:211–9. https://doi.org/10.23938/ASSN.0025.

[98] Wang L-J, Lin P-Y, Lee Y, Huang Y-C, Hsu S-T, Hung C-F, et al. Validation of the Chinese version of Brief Assessment of Cognition in Schizophrenia. Neuropsychiatr Dis Treat 2016;12:2819–26. https://doi.org/10.2147/NDT.S118110.

[99] Sachs G, Winklbaur B, Jagsch R, Keefe RSE. Validation of the German version of the brief assessment of cognition in Schizophrenia (BACS) - preliminary results. Eur Psychiatry J Assoc Eur Psychiatr 2011;26:74–7. https://doi.org/10.1016/j.eurpsy.2009.10.006.

[100] Bralet M-C, Falissard B, Neveu X, Lucas-Ross M, Eskenazi A-M, Keefe RSE. Validation of the French version of the BACS (the brief assessment of cognition in schizophrenia) among 50 French schizophrenic patients. Eur Psychiatry J Assoc Eur Psychiatr 2007;22:365–70. https://doi.org/10.1016/j.eurpsy.2007.02.001.

[101] Segarra N, Bernardo M, Gutierrez F, Justicia A, Fernadez-Egea E, Allas M, et al. Spanish validation of the Brief Assessment in Cognition in Schizophrenia (BACS) in patients with schizophrenia and healthy controls. Eur Psychiatry J Assoc Eur Psychiatr 2011;26:69–73. https://doi.org/10.1016/j.eurpsy.2009.11.001.

[102] Anselmetti S, Poletti S, Ermoli E, Bechi M, Cappa S, Venneri A, et al. The Brief Assessment of Cognition in Schizophrenia. Normative data for the Italian population. Neurol Sci Off J Ital Neurol Soc Ital Soc Clin Neurophysiol 2008;29:85–92. https://doi.org/10.1007/s10072-008-0866-9.

[103] Kaneda Y, Sumiyoshi T, Keefe R, Ishimoto Y, Numata S, Ohmori T. Brief assessment of cognition in schizophrenia: validation of the Japanese version. Psychiatry Clin Neurosci 2007;61:602–9. https://doi.org/10.1111/j.1440-1819.2007.01725.x.

[104] Mazhari S, Parvaresh N, Eslami Shahrbabaki M, Sadeghi MM, Nakhaee N, Keefe RSE. Validation of the Persian version of the brief assessment of cognition in schizophrenia in patients with schizophrenia and healthy controls. Psychiatry Clin Neurosci 2014;68:160–6. https://doi.org/10.1111/pcn.12107.

[105] Araújo GE, Resende CB de, Cardoso ACA, Teixeira AL, Keefe RSE, Salgado JV. Validity and reliability of the Brazilian Portuguese version of the BACS (Brief Assessment of Cognition in Schizophrenia). Clin Sao Paulo Braz 2015;70:278–82. https://doi.org/10.6061/clinics/2015(04)10.

[106] Purdon S. Purdon (2005) SCIP Manual. 2005.

[107] Nuechterlein KH, Green MF, Kern RS, Baade LE, Barch DM, Cohen JD, et al. The MATRICS Consensus Cognitive Battery, part 1: test selection, reliability, and validity. Am J Psychiatry 2008;165:203–13. https://doi.org/10.1176/appi.ajp.2007.07010042.

[108] Fervaha G, Agid O, Foussias G, Remington G. Toward a more parsimonious assessment of neurocognition in schizophrenia: a 10-minute assessment tool. J Psychiatr Res 2014;52:50–6. https://doi.org/10.1016/j.jpsychires.2014.01.009.

[109] Hurford IM, Ventura J, Marder SR, Reise SP, Bilder RM. A 10-minute measure of global cognition: Validation of the Brief Cognitive Assessment Tool for Schizophrenia (B-CATS). Schizophr Res 2018;195:327–33. https://doi.org/10.1016/j.schres.2017.08.033.

[110] Keefe RSE, Poe M, Walker TM, Kang JW, Harvey PD. The Schizophrenia Cognition Rating Scale: an interview-based assessment and its relationship to cognition, real-world functioning, and functional capacity. Am J Psychiatry 2006;163:426–32. https://doi.org/10.1176/appi.ajp.163.3.426.

[111] Harvey PD, Khan A, Atkins A, Walker TM, Keefe RSE. Comprehensive review of the research employing the schizophrenia cognition rating scale (SCoRS). Schizophr Res 2019;210:30–8. https://doi.org/10.1016/j.schres.2019.05.040.

[112] Ventura J, Cienfuegos A, Boxer O, Bilder R. Clinical global impression of cognition in schizophrenia (CGI-CogS): reliability and validity of a co-primary measure of cognition. Schizophr Res 2008;106:59–69. https://doi.org/10.1016/j.schres.2007.07.025.

[113] Ventura J, Reise SP, Keefe RSE, Baade LE, Gold JM, Green MF, et al. The Cognitive Assessment Interview (CAI): development and validation of an empirically derived, brief interview-based measure of cognition. Schizophr Res 2010;121:24–31. https://doi.org/10.1016/j.schres.2010.04.016.

[114] Palumbo D. Inter-rater reliability and psychometric characteristics of the Italian version of the Cognitive Assessment Interview (CAI). Off J Ital Soc Psychopathol 2019;25:85–114.

[115] Bosgelmez S, Yildiz M, Yazici E, Inan E, Turgut C, Karabulut U, et al. Reliability and validity of the Turkish version of cognitive assessment interview (CAI-TR). Klin Psikofarmakol Bül Bull Clin Psychopharmacol 2015;25:365–80. https://doi.org/10.5455/bcp.20150502064017.

[116] Sánchez-Torres AM, Elosúa MR, Lorente-Omeñaca R, Moreno-Izco L, Peralta V, Ventura J, et al. Using the cognitive assessment interview to screen cognitive impairment in psychosis. Eur Arch Psychiatry Clin Neurosci 2016;266:629–37. https://doi.org/10.1007/s00406-016-0700-y.

[117] Salgado JV, Carvalhaes CFR, Pires A de M, Neves M de CL das, Cruz BF, Cardoso CS, et al. Sensitivity and applicability of the Brazilian version of the Brief Assessment of Cognition in Schizophrenia (BACS). Dement Neuropsychol 2007;1:260–5. https://doi.org/10.1590/S1980-57642008DN10300007.

[118] Chianetta J-M, Lefebvre M, LeBlanc R, Grignon S. Comparative psychometric properties of the BACS and RBANS in patients with schizophrenia and schizoaffective disorder. Schizophr Res 2008;105:86–94. https://doi.org/10.1016/j.schres.2008.05.024.

[119] Keefe RSE, Harvey PD, Goldberg TE, Gold JM, Walker TM, Kennel C, et al. Norms and standardization of the Brief Assessment of Cognition in Schizophrenia (BACS). Schizophr Res 2008;102:108–15. https://doi.org/10.1016/j.schres.2008.03.024.

[120] Kern RS, Nuechterlein KH, Green MF, Baade LE, Fenton WS, Gold JM, et al. The MATRICS Consensus Cognitive Battery, part 2: co-norming and standardization. Am J Psychiatry 2008;165:214–20. https://doi.org/10.1176/appi.ajp.2007.07010043.

[121] Pino O, Guilera G, Rojo JE, Gómez-Benito J, Bernardo M, Crespo-Facorro B, et al. Spanish version of the Screen for Cognitive Impairment in Psychiatry (SCIP-S): psychometric properties of a brief scale for cognitive evaluation in schizophrenia. Schizophr Res 2008;99:139–48. https://doi.org/10.1016/j.schres.2007.09.012.

[122] Hurford IM, Marder SR, Keefe RSE, Reise SP, Bilder RM. A brief cognitive assessment tool for schizophrenia: construction of a tool for clinicians. Schizophr Bull 2011;37:538–45. https://doi.org/10.1093/schbul/sbp095.

[123] Keefe RSE, Fox KH, Harvey PD, Cucchiaro J, Siu C, Loebel A. Characteristics of the MATRICS Consensus Cognitive Battery in a 29-site antipsychotic schizophrenia clinical trial. Schizophr Res 2011;125:161–8. https://doi.org/10.1016/j.schres.2010.09.015.

[124] August SM, Kiwanuka JN, McMahon RP, Gold JM. The MATRICS Consensus Cognitive Battery (MCCB): clinical and cognitive correlates. Schizophr Res 2012;134:76–82. https://doi.org/10.1016/j.schres.2011.10.015.

[125] Mohn C, Sundet K, Rund BR. The Norwegian standardization of the MATRICS (Measurement and Treatment Research to Improve Cognition in Schizophrenia) Consensus Cognitive Battery. J Clin Exp Neuropsychol 2012;34:667–77. https://doi.org/10.1080/13803395.2012.667792.

[126] Rodriguez-Jimenez R, Bagney A, Garcia-Navarro C, Aparicio AI, Lopez-Anton R, Moreno-Ortega M, et al. The MATRICS consensus cognitive battery (MCCB): co-norming and standardization in Spain. Schizophr Res 2012;134:279–84. https://doi.org/10.1016/j.schres.2011.11.026.

[127] Eng GK, Lam M, Bong YL, Subramaniam M, Bautista D, Rapisarda A, et al. Brief assessment of cognition in schizophrenia: normative data in an English-speaking ethnic Chinese sample. Arch Clin Neuropsychol Off J Natl Acad Neuropsychol 2013;28:845–58. https://doi.org/10.1093/arclin/act060.

[128] Kaneda Y, Ohmori T, Okahisa Y, Sumiyoshi T, Pu S, Ueoka Y, et al. Measurement and Treatment Research toImprove Cognition in Schizophrenia Consensus Cognitive Battery: validation of the Japanese version. Psychiatry Clin Neurosci 2013;67:182–8. https://doi.org/10.1111/pcn.12029.

[129] Rapisarda A, Lim TF, Lim M, Collinson SL, Kraus MS, Keefe RSE. Applicability of the MATRICS Consensus Cognitive Battery in Singapore. Clin Neuropsychol 2013;27:455–69. https://doi.org/10.1080/13854046.2012.762120.

[130] Roseberry JE, Kristian Hill S. Limited practice effects and evaluation of expectation for change: MATRICS Consensus Cognitive Battery. Schizophr Res 2014;159:188–92. https://doi.org/10.1016/j.schres.2014.08.004.

[131] Fervaha G, Hill C, Agid O, Takeuchi H, Foussias G, Siddiqui I, et al. Examination of the validity of the Brief Neurocognitive Assessment (BNA) for schizophrenia. Schizophr Res 2015;166:304–9. https://doi.org/10.1016/j.schres.2015.05.015.

[132] Jędrasik-Styła M, Ciołkiewicz A, Styła R, Linke M, Parnowska D, Gruszka A, et al. The Polish Academic Version of the MATRICS Consensus Cognitive Battery (MCCB): Evaluation of Psychometric Properties. Psychiatr Q 2015;86:435–47. https://doi.org/10.1007/s11126-015-9343-9.

[133] Lees J, Applegate E, Emsley R, Lewis S, Michalopoulou P, Collier T, et al. Calibration and cross-validation of MCCB and CogState in schizophrenia. Psychopharmacology (Berl) 2015;232:3873–82. https://doi.org/10.1007/s00213-015-3960-8.

[134] Shi C, Kang L, Yao S, Ma Y, Li T, Liang Y, et al. The MATRICS Consensus Cognitive Battery (MCCB): Co-norming and standardization in China. Schizophr Res 2015;169:109–15. https://doi.org/10.1016/j.schres.2015.09.003.

[135] John AP, Yeak K, Ayres H, Sevastos M, Moore E. Successful evaluation of cognitive function and the nature of cognitive deficits among people with schizophrenia in clinical rehabilitation settings. Australas Psychiatry Bull R Aust N Z Coll Psychiatr 2016;24:342–6. https://doi.org/10.1177/1039856216656537.

[136] Kumar S, Mulsant BH, Tsoutsoulas C, Ghazala Z, Voineskos AN, Bowie CR, et al. An optimal combination of MCCB and CANTAB to assess functional capacity in older individuals with schizophrenia. Int J Geriatr Psychiatry 2016;31:1116–23. https://doi.org/10.1002/gps.4547.

[137] Fonseca AO, Berberian AA, de Meneses-Gaya C, Gadelha A, Vicente M de O, Nuechterlein KH, et al. The Brazilian standardization of the MATRICS consensus cognitive battery (MCCB): Psychometric study. Schizophr Res 2017;185:148–53. https://doi.org/10.1016/j.schres.2017.01.006.

[138] Georgiades A, Davis VG, Atkins AS, Khan A, Walker TW, Loebel A, et al. Psychometric characteristics of the MATRICS Consensus Cognitive Battery in a large pooled cohort of stable schizophrenia patients. Schizophr Res 2017;190:172–9. https://doi.org/10.1016/j.schres.2017.03.040.

[139] Wang L-J, Huang Y-C, Hung C-F, Chen C-K, Chen Y-C, Lee P-Y, et al. The Chinese Version of the Brief Assessment of Cognition in Schizophrenia: Data of a Large-Scale Mandarin-Speaking Population. Arch Clin Neuropsychol Off J Natl Acad Neuropsychol 2017;32:289–96. https://doi.org/10.1093/arclin/acw100.

[140] Gómez-Benito J, Berrío ÁI, Guilera G, Rojo E, Purdon S, Pino O. The Screen for Cognitive Impairment in Psychiatry: Proposal for a polytomous scoring system. Int J Methods Psychiatr Res 2018;27:e1598. https://doi.org/10.1002/mpr.1598.

[141] Mucci A, Galderisi S, Green MF, Nuechterlein K, Rucci P, Gibertoni D, et al. Familial aggregation of MATRICS Consensus Cognitive Battery scores in a large sample of outpatients with schizophrenia and their unaffected relatives. Psychol Med 2018;48:1359–66. https://doi.org/10.1017/S0033291717002902.

[142] Belvederi Murri M, Folesani F, Costa S, Biancosino B, Colla C, Zerbinati L, et al. Screening for cognitive impairment in non-affective psychoses: A comparison between the SCIP and the MoCA. Schizophr Res 2020;218:188–94. https://doi.org/10.1016/j.schres.2020.01.005.

[143] Bezdicek O, Michalec J, Kališová L, Kufa T, Děchtěrenko F, Chlebovcová M, et al. Profile of cognitive deficits in schizophrenia and factor structure of the Czech MATRICS Consensus Cognitive Battery. Schizophr Res 2020;218:85–92. https://doi.org/10.1016/j.schres.2020.02.004.

[144] Green MF, Nuechterlein KH, Kern RS, Baade LE, Fenton WS, Gold JM, et al. Functional co-primary measures for clinical trials in schizophrenia: results from the MATRICS Psychometric and Standardization Study. Am J Psychiatry 2008;165:221–8. https://doi.org/10.1176/appi.ajp.2007.07010089.

[145] Harvey PD, Ogasa M, Cucchiaro J, Loebel A, Keefe RSE. Performance and interview-based assessments of cognitive change in a randomized, double-blind comparison of lurasidone vs. ziprasidone. Schizophr Res 2011;127:188–94. https://doi.org/10.1016/j.schres.2011.01.004.

[146] Gonzalez JM, Rubin M, Fredrick MM, Velligan DI. A qualitative assessment of cross-cultural adaptation of intermediate measures for schizophrenia in multisite international studies. Psychiatry Res 2013;206:166–72. https://doi.org/10.1016/j.psychres.2012.10.015.

[147] Ventura J, Reise SP, Keefe RSE, Hurford IM, Wood RC, Bilder RM. The Cognitive Assessment Interview (CAI): reliability and validity of a brief interview-based measure of cognition. Schizophr Bull 2013;39:583–91. https://doi.org/10.1093/schbul/sbs001.

[148] Vita A, Deste G, Barlati S, De Peri L, Giambra A, Poli R, et al. Interview-based assessment of cognition in schizophrenia: applicability of the Schizophrenia Cognition Rating Scale (SCoRS) in different phases of illness and settings of care. Schizophr Res 2013;146:217–23. https://doi.org/10.1016/j.schres.2013.02.035.

[149] Keefe RSE, Davis VG, Spagnola NB, Hilt D, Dgetluck N, Ruse S, et al. Reliability, validity and treatment sensitivity of the Schizophrenia Cognition Rating Scale. Eur Neuropsychopharmacol J Eur Coll Neuropsychopharmacol 2015;25:176–84. https://doi.org/10.1016/j.euroneuro.2014.06.009.

[150] Higuchi Y, Sumiyoshi T, Seo T, Suga M, Takahashi T, Nishiyama S, et al. Associations between daily living skills, cognition, and real-world functioning across stages of schizophrenia; a study with the Schizophrenia Cognition Rating Scale Japanese version. Schizophr Res Cogn 2017;7:13–8. https://doi.org/10.1016/j.scog.2017.01.001.

[151] Kang E-C, Kim S-J, Seo Y-S, Jung S-S, Seo B-J, Ryu J-W, et al. The Korean Version of the Schizophrenia Cognition Rating Scale: Reliability and Validity. Psychiatry Investig 2017;14:141–9. https://doi.org/10.4306/pi.2017.14.2.141.

[152] Mazhari S, Ghafaree-Nejad AR, Soleymani-Zade S, Keefe RSE. Validation of the Persian version of the Schizophrenia Cognition Rating Scale (SCoRS) in patients with schizophrenia. Asian J Psychiatry 2017;27:12–5. https://doi.org/10.1016/j.ajp.2017.02.007.

[153] Corcoran R, Mercer G, Frith CD. Schizophrenia, symptomatology and social inference: investigating “theory of mind” in people with schizophrenia. Schizophr Res 1995;17:5–13. https://doi.org/10.1016/0920-9964(95)00024-g.

[154] Janssen I, Krabbendam L, Jolles J, van Os J. Alterations in theory of mind in patients with schizophrenia and non-psychotic relatives. Acta Psychiatr Scand 2003;108:110–7. https://doi.org/10.1034/j.1600-0447.2003.00092.x.

[155] Gil D, Fernández-Modamio M, Bengochea R, Arrieta M. [Adaptation of the Hinting Task theory of the mind test to Spanish]. Rev Psiquiatr Salud Ment 2012;5:79–88. https://doi.org/10.1016/j.rpsm.2011.11.004.

[156] Sanvicente-Vieira B, Brietzke E, Grassi-Oliveira R. Translation and adaptation of Theory of Mind tasks into Brazilian portuguese. Trends Psychiatry Psychother 2012;34:178–85. https://doi.org/10.1590/s2237-60892012000400003.

[157] Frøyhaug M, Andersson S, Andreassen OA, Ueland T, Vaskinn A. Theory of mind in schizophrenia and bipolar disorder: psychometric properties of the Norwegian version of the Hinting Task. Cognit Neuropsychiatry 2019;24:454–69. https://doi.org/10.1080/13546805.2019.1674645.

[158] Krawczyk MM, Schudy AM, Jarkiewicz M, Okruszek Ł. Polish version of the Hinting Task - pilot study with patients with schizophrenia. Psychiatr Pol 2020;54:727–39. https://doi.org/10.12740/PP/112265.

[159] Lim K, Lee S-A, Pinkham AE, Lam M, Lee J. Evaluation of social cognitive measures in an Asian schizophrenia sample. Schizophr Res Cogn 2020;20:100169. https://doi.org/10.1016/j.scog.2019.100169.

[160] Hur J-W, Byun MS, Shin NY, Shin YS, Kim SN, Jang JH, et al. General intellectual functioning as a buffer against theory-of-mind deficits in individuals at ultra-high risk for psychosis. Schizophr Res 2013;149:83–7. https://doi.org/10.1016/j.schres.2013.06.019.

[161] Okano H, Kubota R, Okubo R, Hashimoto N, Ikezawa S, Toyomaki A, et al. Evaluation of Social Cognition Measures for Japanese Patients with Schizophrenia Using an Expert Panel and Modified Delphi Method. J Pers Med 2021;11:275. https://doi.org/10.3390/jpm11040275.

[162] Bell M, Bryson G, Lysaker P. Positive and negative affect recognition in schizophrenia: a comparison with substance abuse and normal control subjects. Psychiatry Res 1997;73:73–82. https://doi.org/10.1016/s0165-1781(97)00111-x.

[163] Velotti P, Garofalo C, Petrocchi C, Parisi M, Santini ML, Zavattini GC. Bell Lysaker Emotion Recognition Test: a Contribution for the Italian Validation. Eur Psychiatry 2015;30:1–1. https://doi.org/10.1016/S0924-9338(15)30640-4.

[164] Adolphs R, Tranel D, Damasio AR. The human amygdala in social judgment. Nature 1998;393:470–4. https://doi.org/10.1038/30982.

[165] Baron-Cohen S, Wheelwright S, Hill J, Raste Y, Plumb I. The “Reading the Mind in the Eyes” Test revised version: a study with normal adults, and adults with Asperger syndrome or high-functioning autism. J Child Psychol Psychiatry 2001;42:241–51.

[166] Vellante M, Baron-Cohen S, Melis M, Marrone M, Petretto DR, Masala C, et al. The “Reading the Mind in the Eyes” test: systematic review of psychometric properties and a validation study in Italy. Cognit Neuropsychiatry 2013;18:326–54. https://doi.org/10.1080/13546805.2012.721728.

[167] Lee H-R, Nam G, Hur J-W. Development and validation of the Korean version of the Reading the Mind in the Eyes Test. PloS One 2020;15:e0238309. https://doi.org/10.1371/journal.pone.0238309.

[168] Djordjevic. Psychometric evaluation and validation of the Serbian version of “Reading the Mind in the Eyes” test. Psihologija 2017;50:483–502.

[169] Prevost M, Carrier M-E, Chowne G, Zelkowitz P, Joseph L, Gold I. The Reading the Mind in the Eyes test: validation of a French version and exploration of cultural variations in a multi-ethnic city. Cognit Neuropsychiatry 2014;19:189–204. https://doi.org/10.1080/13546805.2013.823859.

[170] Chakrabarty M, Dasgupta G, Acharya R, Chatterjee SS, Guha P, Belmonte MK, et al. Validation of revised reading the mind in the eyes test in the Indian (Bengali) population: A preliminary study. Indian J Psychiatry 2021;63:74–9. https://doi.org/10.4103/psychiatry.IndianJPsychiatry_967_20.

[171] Mayer JD, Salovey P, Caruso DR, Sitarenios G. Measuring emotional intelligence with the MSCEIT V2.0. Emot Wash DC 2003;3:97–105. https://doi.org/10.1037/1528-3542.3.1.97.

[172] McDonald S, Flanagan S, Rollins J, Kinch J. TASIT: A new clinical tool for assessing social perception after traumatic brain injury. J Head Trauma Rehabil 2003;18:219–38. https://doi.org/10.1097/00001199-200305000-00001.

[173] Rocca P, Galderisi S, Rossi A, Bertolino A, Rucci P, Gibertoni D, et al. Social cognition in people with schizophrenia: a cluster-analytic approach. Psychol Med 2016;46:2717–29. https://doi.org/10.1017/S0033291716001100.

[174] Kohler CG, Turner TH, Gur RE, Gur RC. Recognition of facial emotions in neuropsychiatric disorders. CNS Spectr 2004;9:267–74. https://doi.org/10.1017/s1092852900009202.

[175] Assogna F, Pontieri FE, Cravello L, Peppe A, Pierantozzi M, Stefani A, et al. Intensity-dependent facial emotion recognition and cognitive functions in Parkinson’s disease. J Int Neuropsychol Soc JINS 2010;16:867–76. https://doi.org/10.1017/S1355617710000755.

[176] French n.d.

[177] Combs DR, Penn DL, Wicher M, Waldheter E. The Ambiguous Intentions Hostility Questionnaire (AIHQ): a new measure for evaluating hostile social-cognitive biases in paranoia. Cognit Neuropsychiatry 2007;12:128–43. https://doi.org/10.1080/13546800600787854.

[178] Sergi MJ, Fiske AP, Horan WP, Kern RS, Kee KS, Subotnik KL, et al. Development of a measure of relationship perception in schizophrenia. Psychiatry Res 2009;166:54–62. https://doi.org/10.1016/j.psychres.2008.03.010.

[179] Bell MD, Fiszdon JM, Greig TC, Wexler BE. Social attribution test--multiple choice (SAT-MC) in schizophrenia: comparison with community sample and relationship to neurocognitive, social cognitive and symptom measures. Schizophr Res 2010;122:164–71. https://doi.org/10.1016/j.schres.2010.03.024.

[180] Bänziger T, Scherer KR, Hall JA, Rosenthal R. Introducing the MiniPONS: A short multichannel version of the Profile of Nonverbal Sensitivity (PONS). J Nonverbal Behav 2011;35:189–204. https://doi.org/10.1007/s10919-011-0108-3.

[181] Martínez-Sánchez F, G. Fernández-Abascal E, Martínez-Modia JC. Adaptación española de la versión reducida multicanal del Perfil de Sensibilidad No Verbal (MiniPONS). An Psicol 2013;29:604–13. https://doi.org/10.6018/analesps.29.2.161851.

[182] Rosset E. It’s no accident: Our bias for intentional explanations. Cognition 2008;108:771–80. https://doi.org/10.1016/j.cognition.2008.07.001.

[183] Bell M, Bryson G, Greig T, Corcoran C, Wexler BE. Neurocognitive enhancement therapy with work therapy: effects on neuropsychological test performance. Arch Gen Psychiatry 2001;58:763–8. https://doi.org/10.1001/archpsyc.58.8.763.

[184] Bryson G, Bell MD. Initial and final work performance in schizophrenia: cognitive and symptom predictors. J Nerv Ment Dis 2003;191:87–92. https://doi.org/10.1097/01.NMD.0000050937.06332.3C.

[185] Combs DR, Gouvier WD. The role of attention in affect perception: an examination of Mirsky’s four factor model of attention in chronic schizophrenia. Schizophr Bull 2004;30:727–38. https://doi.org/10.1093/oxfordjournals.schbul.a007126.

[186] Wexler BE, Bell MD. Cognitive remediation and vocational rehabilitation for schizophrenia. Schizophr Bull 2005;31:931–41. https://doi.org/10.1093/schbul/sbi038.

[187] Nienow TM, Docherty NM, Cohen AS, Dinzeo TJ. Attentional dysfunction, social perception, and social competence: what is the nature of the relationship? J Abnorm Psychol 2006;115:408–17. https://doi.org/10.1037/0021-843X.115.3.408.

[188] Pinkham AE, Penn DL. Neurocognitive and social cognitive predictors of interpersonal skill in schizophrenia. Psychiatry Res 2006;143:167–78. https://doi.org/10.1016/j.psychres.2005.09.005.

[189] Bell M, Tsang HWH, Greig TC, Bryson GJ. Neurocognition, social cognition, perceived social discomfort, and vocational outcomes in schizophrenia. Schizophr Bull 2009;35:738–47. https://doi.org/10.1093/schbul/sbm169.

[190] Roberts DL, Penn DL. Social cognition and interaction training (SCIT) for outpatients with schizophrenia: a preliminary study. Psychiatry Res 2009;166:141–7. https://doi.org/10.1016/j.psychres.2008.02.007.

[191] Fiszdon JM, Johannesen JK. Functional significance of preserved affect recognition in schizophrenia. Psychiatry Res 2010;176:120–5. https://doi.org/10.1016/j.psychres.2009.08.006.

[192] Hamm JA, Renard SB, Fogley RL, Leonhardt BL, Dimaggio G, Buck KD, et al. Metacognition and social cognition in schizophrenia: stability and relationship to concurrent and prospective symptom assessments. J Clin Psychol 2012;68:1303–12. https://doi.org/10.1002/jclp.21906.

[193] Fiszdon JM, Fanning JR, Johannesen JK, Bell MD. Social cognitive deficits in schizophrenia and their relationship to clinical and functional status. Psychiatry Res 2013;205:25–9. https://doi.org/10.1016/j.psychres.2012.08.041.

[194] Kohler CG, Turner TH, Bilker WB, Brensinger CM, Siegel SJ, Kanes SJ, et al. Facial emotion recognition in schizophrenia: intensity effects and error pattern. Am J Psychiatry 2003;160:1768–74. https://doi.org/10.1176/appi.ajp.160.10.1768.

[195] Silver H, Goodman C, Knoll G, Isakov V. Brief emotion training improves recognition of facial emotions in chronic schizophrenia. A pilot study. Psychiatry Res 2004;128:147–54. https://doi.org/10.1016/j.psychres.2004.06.002.

[196] Goodman C, Knoll G, Isakov V, Silver H. Insight into illness in schizophrenia. Compr Psychiatry 2005;46:284–90. https://doi.org/10.1016/j.comppsych.2004.11.002.

[197] Gur RE, Loughead J, Kohler CG, Elliott MA, Lesko K, Ruparel K, et al. Limbic activation associated with misidentification of fearful faces and flat affect in schizophrenia. Arch Gen Psychiatry 2007;64:1356–66. https://doi.org/10.1001/archpsyc.64.12.1356.

[198] Weiss EM, Stadelmann E, Kohler CG, Brensinger CM, Nolan KA, Oberacher H, et al. Differential effect of catechol-O-methyltransferase Val158Met genotype on emotional recognition abilities in healthy men and women. J Int Neuropsychol Soc JINS 2007;13:881–7. https://doi.org/10.1017/S1355617707070932.

[199] Pinkham AE, Sasson NJ, Calkins ME, Richard J, Hughett P, Gur RE, et al. The other-race effect in face processing among African American and Caucasian individuals with schizophrenia. Am J Psychiatry 2008;165:639–45. https://doi.org/10.1176/appi.ajp.2007.07101604.

[200] Carter CS, Barch DM, Gur R, Gur R, Pinkham A, Ochsner K. CNTRICS final task selection: social cognitive and affective neuroscience-based measures. Schizophr Bull 2009;35:153–62. https://doi.org/10.1093/schbul/sbn157.

[201] Grant PM, Beck AT. Asocial beliefs as predictors of asocial behavior in schizophrenia. Psychiatry Res 2010;177:65–70. https://doi.org/10.1016/j.psychres.2010.01.005.

[202] Gur RC, Richard J, Hughett P, Calkins ME, Macy L, Bilker WB, et al. A cognitive neuroscience-based computerized battery for efficient measurement of individual differences: standardization and initial construct validation. J Neurosci Methods 2010;187:254–62. https://doi.org/10.1016/j.jneumeth.2009.11.017.

[203] Pinkham AE, Brensinger C, Kohler C, Gur RE, Gur RC. Actively paranoid patients with schizophrenia over attribute anger to neutral faces. Schizophr Res 2011;125:174–8. https://doi.org/10.1016/j.schres.2010.11.006.

[204] Irani F, Brensinger CM, Richard J, Calkins ME, Moberg PJ, Bilker W, et al. Computerized neurocognitive test performance in schizophrenia: a lifespan analysis. Am J Geriatr Psychiatry Off J Am Assoc Geriatr Psychiatry 2012;20:41–52. https://doi.org/10.1097/JGP.0b013e3182051a7d.

[205] Extremera N, Fernández-Berrocal P, Salovey P. Spanish version of the Mayer-Salovey-Caruso Emotional Intelligence Test (MSCEIT). Version 2.0: reliabilities, age and gender differences. Psicothema 2006;18 Suppl:42–8.

[206] Kee KS, Horan WP, Salovey P, Kern RS, Sergi MJ, Fiske AP, et al. Emotional intelligence in schizophrenia. Schizophr Res 2009;107:61–8. https://doi.org/10.1016/j.schres.2008.08.016.

[207] Eack SM, Mermon DE, Montrose DM, Miewald J, Gur RE, Gur RC, et al. Social cognition deficits among individuals at familial high risk for schizophrenia. Schizophr Bull 2010;36:1081–8. https://doi.org/10.1093/schbul/sbp026.

[208] Karim S, Minhas HM, Bhattacharya S, Sein K, Nayar B, Morris J, et al. The symptomatology of Alzheimer’s disease: a cross-cultural study. Int J Geriatr Psychiatry 2011;26:415–22. https://doi.org/10.1002/gps.2544.

[209] Curci A, Lanciano T, Soleti E, Zammuner VL, Salovey P. Construct validity of the Italian version of the Mayer-Salovey-Caruso Emotional Intelligence Test (MSCEIT) v2.0. J Pers Assess 2013;95:486–94. https://doi.org/10.1080/00223891.2013.778272.

[210] Lindenmayer J-P, McGurk SR, Khan A, Kaushik S, Thanju A, Hoffman L, et al. Improving social cognition in schizophrenia: a pilot intervention combining computerized social cognition training with cognitive remediation. Schizophr Bull 2013;39:507–17. https://doi.org/10.1093/schbul/sbs120.

[211] Mao W-C, Chen L-F, Chi C-H, Lin C-H, Kao Y-C, Hsu W-Y, et al. Traditional Chinese version of the Mayer Salovey Caruso Emotional Intelligence Test (MSCEIT-TC): Its validation and application to schizophrenic individuals. Psychiatry Res 2016;243:61–70. https://doi.org/10.1016/j.psychres.2016.04.107.

[212] McCleery A, Lee J, Fiske AP, Ghermezi L, Hayata JN, Hellemann GS, et al. Longitudinal stability of social cognition in schizophrenia: A 5-year follow-up of social perception and emotion processing. Schizophr Res 2016;176:467–72. https://doi.org/10.1016/j.schres.2016.07.008.

[213] DeTore NR, Mueser KT, McGurk SR. What does the Managing Emotions branch of the MSCEIT add to the MATRICS consensus cognitive battery? Schizophr Res 2018;197:414–20. https://doi.org/10.1016/j.schres.2018.02.018.

[214] Corcoran R. Inductive reasoning and the understanding of intention in schizophrenia. Cognit Neuropsychiatry 2003;8:223–35. https://doi.org/10.1080/13546800244000319.

[215] Corcoran R, Frith CD. Autobiographical memory and theory of mind: evidence of a relationship in schizophrenia. Psychol Med 2003;33:897–905. https://doi.org/10.1017/s0033291703007529.

[216] Greig TC, Bryson GJ, Bell MD. Theory of mind performance in schizophrenia: diagnostic, symptom, and neuropsychological correlates. J Nerv Ment Dis 2004;192:12–8. https://doi.org/10.1097/01.nmd.0000105995.67947.fc.

[217] Marjoram D, Gardner C, Burns J, Miller P, Lawrie SM, Johnstone EC. Symptomatology and social inference: a theory of mind study of schizophrenia and psychotic affective disorder. Cognit Neuropsychiatry 2005;10:347–59. https://doi.org/10.1080/13546800444000092.

[218] Penn D, Roberts DL, Munt ED, Silverstein E, Jones N, Sheitman B. A pilot study of social cognition and interaction training (SCIT) for schizophrenia. Schizophr Res 2005;80:357–9. https://doi.org/10.1016/j.schres.2005.07.011.

[219] Silverstein SM, Wallace CJ, Schenkel LS. The micro-module learning tests: work-sample assessments of responsiveness to skills training. Schizophr Bull 2005;31:73–83. https://doi.org/10.1093/schbul/sbi008.

[220] Schenkel LS, Spaulding WD, Silverstein SM. Poor premorbid social functioning and theory of mind deficit in schizophrenia: evidence of reduced context processing? J Psychiatr Res 2005;39:499–508. https://doi.org/10.1016/j.jpsychires.2005.01.001.

[221] Bora E, Eryavuz A, Kayahan B, Sungu G, Veznedaroglu B. Social functioning, theory of mind and neurocognition in outpatients with schizophrenia; mental state decoding may be a better predictor of social functioning than mental state reasoning. Psychiatry Res 2006;145:95–103. https://doi.org/10.1016/j.psychres.2005.11.003.

[222] Uhlhaas PJ, Phillips WA, Schenkel LS, Silverstein SM. Theory of mind and perceptual context-processing in schizophrenia. Cognit Neuropsychiatry 2006;11:416–36. https://doi.org/10.1080/13546800444000272.

[223] Bertrand M-C, Sutton H, Achim AM, Malla AK, Lepage M. Social cognitive impairments in first episode psychosis. Schizophr Res 2007;95:124–33. https://doi.org/10.1016/j.schres.2007.05.033.

[224] Bora E, Sehitoglu G, Aslier M, Atabay I, Veznedaroglu B. Theory of mind and unawareness of illness in schizophrenia: is poor insight a mentalizing deficit? Eur Arch Psychiatry Clin Neurosci 2007;257:104–11. https://doi.org/10.1007/s00406-006-0681-3.

[225] Mizrahi R, Korostil M, Starkstein SE, Zipursky RB, Kapur S. The effect of antipsychotic treatment on Theory of Mind. Psychol Med 2007;37:595–601. https://doi.org/10.1017/S0033291706009342.

[226] Bora E, Gökçen S, Kayahan B, Veznedaroglu B. Deficits of social-cognitive and social-perceptual aspects of theory of mind in remitted patients with schizophrenia: effect of residual symptoms. J Nerv Ment Dis 2008;196:95–9. https://doi.org/10.1097/NMD.0b013e318162a9e1.

[227] van Hooren S, Versmissen D, Janssen I, Myin-Germeys I, à Campo J, Mengelers R, et al. Social cognition and neurocognition as independent domains in psychosis. Schizophr Res 2008;103:257–65. https://doi.org/10.1016/j.schres.2008.02.022.

[228] Bell MD, Corbera S, Johannesen JK, Fiszdon JM, Wexler BE. Social cognitive impairments and negative symptoms in schizophrenia: are there subtypes with distinct functional correlates? Schizophr Bull 2013;39:186–96. https://doi.org/10.1093/schbul/sbr125.

[229] Couture SM, Granholm EL, Fish SC. A path model investigation of neurocognition, theory of mind, social competence, negative symptoms and real-world functioning in schizophrenia. Schizophr Res 2011;125:152–60. https://doi.org/10.1016/j.schres.2010.09.020.

[230] Liu NH, Choi K-H, Reddy F, Spaulding WD. Heterogeneity and the longitudinal recovery of functioning during inpatient psychiatric rehabilitation for treatment-refractory severe mental illness. Am J Psychiatr Rehabil 2011;14:55–75. https://doi.org/10.1080/15487768.2011.546293.

[231] Lysaker PH, Dimaggio G, Buck KD, Callaway SS, Salvatore G, Carcione A, et al. Poor insight in schizophrenia: links between different forms of metacognition with awareness of symptoms, treatment need, and consequences of illness. Compr Psychiatry 2011;52:253–60. https://doi.org/10.1016/j.comppsych.2010.07.007.

[232] Menon M, Addington J, Remington G. Examining cognitive biases in patients with delusions of reference. Eur Psychiatry J Assoc Eur Psychiatr 2013;28:71–3. https://doi.org/10.1016/j.eurpsy.2011.03.005.

[233] Rubio JL, Ruiz-Veguilla M, Hernández L, Barrigón ML, Salcedo MD, Moreno JM, et al. Jumping to conclusions in psychosis: a faulty appraisal. Schizophr Res 2011;133:199–204. https://doi.org/10.1016/j.schres.2011.08.008.

[234] Choi K-H, Liu N, Spaulding W. Emotional context processing in severe mental illness: scale development and preliminary construct validity. Psychiatry Res 2012;199:84–91. https://doi.org/10.1016/j.psychres.2012.04.014.

[235] Fanning JR, Bell MD, Fiszdon JM. Is it possible to have impaired neurocognition but good social cognition in schizophrenia? Schizophr Res 2012;135:68–71. https://doi.org/10.1016/j.schres.2011.12.009.

[236] Meijer J, Simons CJP, Quee PJ, Verweij K, GROUP Investigators. Cognitive alterations in patients with non-affective psychotic disorder and their unaffected siblings and parents. Acta Psychiatr Scand 2012;125:66–76. https://doi.org/10.1111/j.1600-0447.2011.01777.x.

[237] Tas C, Danaci AE, Cubukcuoglu Z, Brüne M. Impact of family involvement on social cognition training in clinically stable outpatients with schizophrenia -- a randomized pilot study. Psychiatry Res 2012;195:32–8. https://doi.org/10.1016/j.psychres.2011.07.031.

[238] Russell TA, Rubia K, Bullmore ET, Soni W, Suckling J, Brammer MJ, et al. Exploring the social brain in schizophrenia: left prefrontal underactivation during mental state attribution. Am J Psychiatry 2000;157:2040–2. https://doi.org/10.1176/appi.ajp.157.12.2040.

[239] Craig JS, Hatton C, Craig FB, Bentall RP. Persecutory beliefs, attributions and theory of mind: comparison of patients with paranoid delusions, Asperger’s syndrome and healthy controls. Schizophr Res 2004;69:29–33. https://doi.org/10.1016/S0920-9964(03)00154-3.

[240] Kelemen O, Erdélyi R, Pataki I, Benedek G, Janka Z, Kéri S. Theory of mind and motion perception in schizophrenia. Neuropsychology 2005;19:494–500. https://doi.org/10.1037/0894-4105.19.4.494.

[241] Couture SM, Penn DL, Addington J, Woods SW, Perkins DO. Assessment of social judgments and complex mental states in the early phases of psychosis. Schizophr Res 2008;100:237–41. https://doi.org/10.1016/j.schres.2007.12.484.

[242] McGlade N, Behan C, Hayden J, O’Donoghue T, Peel R, Haq F, et al. Mental state decoding v. mental state reasoning as a mediator between cognitive and social function in psychosis. Br J Psychiatry J Ment Sci 2008;193:77–8. https://doi.org/10.1192/bjp.bp.107.044198.

[243] Hallerbäck MU, Lugnegård T, Hjärthag F, Gillberg C. The Reading the Mind in the Eyes Test: test-retest reliability of a Swedish version. Cognit Neuropsychiatry 2009;14:127–43. https://doi.org/10.1080/13546800902901518.

[244] Sparks A, McDonald S, Lino B, O’Donnell M, Green MJ. Social cognition, empathy and functional outcome in schizophrenia. Schizophr Res 2010;122:172–8. https://doi.org/10.1016/j.schres.2010.06.011.

[245] Wynn JK, Sugar C, Horan WP, Kern R, Green MF. Mismatch negativity, social cognition, and functioning in schizophrenia patients. Biol Psychiatry 2010;67:940–7. https://doi.org/10.1016/j.biopsych.2009.11.024.

[246] Johannesen JK, Lurie JB, Fiszdon JM, Bell MD. The Social Attribution Task-Multiple Choice (SAT-MC): A Psychometric and Equivalence Study of an Alternate Form. ISRN Psychiatry 2013;2013:830825. https://doi.org/10.1155/2013/830825.

[247] Johannesen JK, Fiszdon JM, Weinstein A, Ciosek D, Bell MD. The Social Attribution Task - Multiple Choice (SAT-MC): Psychometric comparison with social cognitive measures for schizophrenia research. Psychiatry Res 2018;262:154–61. https://doi.org/10.1016/j.psychres.2018.02.011.

[248] Waldheter EJ, Jones NT, Johnson ER, Penn DL. Utility of social cognition and insight in the prediction of inpatient violence among individuals with a severe mental illness. J Nerv Ment Dis 2005;193:609–18. https://doi.org/10.1097/01.nmd.0000177788.25357.de.

[249] Horan WP, Kern RS, Shokat-Fadai K, Sergi MJ, Wynn JK, Green MF. Social cognitive skills training in schizophrenia: an initial efficacy study of stabilized outpatients. Schizophr Res 2009;107:47–54. https://doi.org/10.1016/j.schres.2008.09.006.

[250] Combs DR, Penn DL, Michael CO, Basso MR, Wiedeman R, Siebenmorgan M, et al. Perceptions of hostility by persons with and without persecutory delusions. Cognit Neuropsychiatry 2009;14:30–52. https://doi.org/10.1080/13546800902732970.

[251] Roberts DL, Penn DL, Labate D, Margolis SA, Sterne A. Transportability and feasibility of Social Cognition And Interaction Training (SCIT) in community settings. Behav Cogn Psychother 2010;38:35–47. https://doi.org/10.1017/S1352465809990464.

[252] Elnakeeb M, Abdel-Dayem S, Gaafar M, Mavundla TR. Attributional style of Egyptians with schizophrenia. Int J Ment Health Nurs 2010;19:445–56. https://doi.org/10.1111/j.1447-0349.2010.00707.x.

[253] Horan WP, Kern RS, Tripp C, Hellemann G, Wynn JK, Bell M, et al. Efficacy and specificity of social cognitive skills training for outpatients with psychotic disorders. J Psychiatr Res 2011;45:1113–22. https://doi.org/10.1016/j.jpsychires.2011.01.015.

[254] Baas D, van’t Wout M, Aleman A, Kahn RS. Social judgement in clinically stable patients with schizophrenia and healthy relatives: behavioural evidence of social brain dysfunction. Psychol Med 2008;38:747–54. https://doi.org/10.1017/S0033291707001729.

[255] Baas D, Aleman A, Vink M, Ramsey NF, de Haan EHF, Kahn RS. Evidence of altered cortical and amygdala activation during social decision-making in schizophrenia. NeuroImage 2008;40:719–27. https://doi.org/10.1016/j.neuroimage.2007.12.039.

[256] Pinkham AE, Hopfinger JB, Pelphrey KA, Piven J, Penn DL. Neural bases for impaired social cognition in schizophrenia and autism spectrum disorders. Schizophr Res 2008;99:164–75. https://doi.org/10.1016/j.schres.2007.10.024.

[257] Couture SM, Penn DL, Losh M, Adolphs R, Hurley R, Piven J. Comparison of social cognitive functioning in schizophrenia and high functioning autism: more convergence than divergence. Psychol Med 2010;40:569–79. https://doi.org/10.1017/S003329170999078X.

[258] Pedersen CA, Gibson CM, Rau SW, Salimi K, Smedley KL, Casey RL, et al. Intranasal oxytocin reduces psychotic symptoms and improves Theory of Mind and social perception in schizophrenia. Schizophr Res 2011;132:50–3. https://doi.org/10.1016/j.schres.2011.07.027.

[259] Buck B, Hester NR, Pinkham A, Harvey PD, Jarskog LF, Penn DL. The bias toward intentionality in schizophrenia: Automaticity, context, and relationships to symptoms and functioning. J Abnorm Psychol 2018;127:503–12. https://doi.org/10.1037/abn0000360.

[260] Pinkham AE, Penn DL, Green MF, Buck B, Healey K, Harvey PD. The social cognition psychometric evaluation study: results of the expert survey and RAND panel. Schizophr Bull 2014;40:813–23. https://doi.org/10.1093/schbul/sbt081.

[261] Pinkham AE, Penn DL, Green MF, Harvey PD. Social Cognition Psychometric Evaluation: Results of the Initial Psychometric Study. Schizophr Bull 2016;42:494–504. https://doi.org/10.1093/schbul/sbv056.

[262] Pinkham AE, Harvey PD, Penn DL. Social Cognition Psychometric Evaluation: Results of the Final Validation Study. Schizophr Bull 2018;44:737–48. https://doi.org/10.1093/schbul/sbx117.
